# A Novel Approach to Obtain Vaccine Effectiveness Continuous Profiles. Example Case: COVID-19 in Elderly Mexicans

**DOI:** 10.3390/vaccines11040719

**Published:** 2023-03-23

**Authors:** Óscar A. González-Sánchez, Luis J. González-Ortiz, M. Judith Sánchez-Peña, Humberto Gutiérrez-Pulido, Oscar Cervantes, Fabiola Márquez-Sandoval, Jorge Hernández-Bello, Norberto Casillas, José Francisco Muñoz-Valle

**Affiliations:** 1 Division of Technologies for the Cyber-Human Integration, University Center of Exact Sciences and Engineering (CUCEI), University of Guadalajara, Marcelino García Barragán 1421, Col. Olímpica, Guadalajara C.P. 44430, Jalisco, Mexico; 2 Department of Chemistry, University Center of Exact Sciences and Engineering (CUCEI), University of Guadalajara, Marcelino García Barragán 1421, Col. Olímpica, Guadalajara C.P. 44430, Jalisco, Mexico; 3 Department of Mathematics, University Center of Exact Sciences and Engineering (CUCEI), University of Guadalajara, Marcelino García Barragán 1421, Col. Olímpica, Guadalajara C.P. 44430, Jalisco, Mexico; 4 Department of Human Reproduction, Child Growth and Development, University Center of Health Sciences (CUCS), University of Guadalajara, Sierra Mojada 950, Col. Independencia, Guadalajara C.P. 44340, Jalisco, Mexico; 5 Institute of Research in Biomedical Sciences, University Center of Health Sciences (CUCS), University of Guadalajara, Sierra Mojada 950, Col. Independencia, Guadalajara C.P. 44340, Jalisco, Mexico

**Keywords:** vaccine effectiveness profile, COVID-19 vaccines, symptomatic disease, time-dependent effectiveness, waning vaccine effectiveness, beneficial effect profile

## Abstract

Population-wide vaccination is the most promising long-term COVID-19 disease management strategy. However, the protection offered by the currently available COVID-19 vaccines wanes over time, requiring boosters to be periodically given, which represents an unattainable challenge, especially if it is necessary to apply several doses per year. Therefore, it is essential to design strategies that contribute to maximizing the control of the pandemic with the available vaccines. Achieving this objective requires knowing, as precisely and accurately as possible, the changes in vaccine effectiveness over time in each population group, considering the eventual dependence on age, sex, etc. Thus, the present work proposes a novel approach to calculating realistic effectiveness profiles against symptomatic disease. In addition, this strategy can be adapted to estimate realistic effectiveness profiles against hospitalizations or deaths. All such time-dependent profiles allow the design of improved vaccination schedules, where each dose can be administrated to the population groups so that the fulfillment of the containment objectives is maximized. As a practical example for this analysis, vaccination against COVID-19 in Mexico was considered. However, this methodology can be applied to other countries’ data or to characterize future vaccines with time-dependent effectiveness values. Since this strategy uses aggregated observational data collected from massive databases, assumptions about the data validity and the course of the studied epidemic could eventually be necessary.

## 1. Introduction

The COVID-19 pandemic is a global public health quandary whose negative consequences have reached magnitudes never seen before [1,2,3,4]. Since its emergence at the end of 2019 [5], universal access to vaccines against this disease has been sought as an effective strategy to reduce mortality rates and mitigate the pandemic [6,7]. Unfortunately, up to now, the administration of the available vaccines has not achieved a total contention of the global pandemic, with achievements among the different countries varying considerably.

Through results obtained in phase 3 clinical trials, several vaccines against COVID-19 have demonstrated their safety and efficacy to international organizations [8,9,10,11,12,13,14,15]. In such studies, the efficacy values for the respective global groups (primary groups) and some subgroups (secondary subgroups) have been reported [9,10,11,12,13,14,15]. However, regardless of the tens of thousands of individuals recruited for each of these such studies, in several of their subgroups, the low number of individuals considered weakened the statistical robustness of the results. The last circumstance prevented the proposal of quantitative conclusions about the dependence of the efficacy on age, sex, race, or some comorbidity. Nevertheless, qualitatively speaking, such a value could be expected to depend on one or several of those parameters.

An even more relevant limitation of such studies is that they do not calculate the vaccine efficacy considering that the efficacy varies with time. Nonetheless, several studies have demonstrated, in real-world conditions, how the vaccine’s effectiveness against symptomatic disease wanes over time [16,17,18,19,20]. Complementarily, in individuals with complete vaccination schemes, a waning in humoral immune responses has been reported [21,22,23,24], which is expected to indirectly indicate the decrement in the effectiveness of the vaccines used. Finally, although the impact of the appearance of variants on vaccine effectiveness has been studied, conclusive results have not yet been obtained [16,17,18,19,21,22,23]. Thus, regardless of the advances in this topic, in quantitative terms, the duration of protection afforded by such vaccines remains quantitatively unknown.

This dependence over time determines that, in order to achieve sustainable pandemic control, it would be necessary to produce, distribute, and apply vaccines several times per year to almost 8 billion human beings. Such a challenge is unfulfillable, at least for now [25,26]. In addition, since this is a global problem, issues that include the concerns of the World Health Organization (WHO) and others about vaccine equity [27,28,29], as well as the non-generalized acceptance of COVID-19 vaccines, at least in certain countries [26,30,31], must be considered. Thus, as long as it is impossible to meet the global need for vaccines, it will be necessary to improve vaccination schedules to maximize, with the local and globally available vaccines, the containment of the pandemic at both levels.

This necessity has motivated several research groups to develop models to simulate different hypothetical scenarios in order to understand the transmission dynamics of the virus [32,33], or propose vaccine prioritization strategies to reduce deaths, hospitalizations, or confirmed cases of COVID-19, among other objectives [34,35,36,37,38]. In such studies, some hypothetical conditions were considered, such as non-time-dependent effectiveness values (E values). Unfortunately, current evidence demonstrates that COVID-19 vaccines do not fulfill this requirement [16,17,18,19,20,21,22,23,24]. Therefore, the real-world applicability of the results obtained in such studies is limited.

Thus, the availability of a procedure to estimate, unbiasedly and with high precision, a continuous E profile (curve describing the evolution over time of the vaccine’s effectiveness since it is administered to each individual) is a prerequisite in order to propose improved vaccination schedules that increase the pandemic containment level. The currently available studies [16,17,18] have only provided a few discrete E values, which were measured for specific groups in different periods during the COVID-19 pandemic. Unfortunately, such values were calculated using the traditional methodology (Equation (1)), which implicitly assumes that vaccine effectiveness is not time-dependent. Consequently, the resulting E values are inevitably biased. Therefore, these values cannot be used to build continuous E profiles that characterize, unbiasedly and precisely, the behavior of the vaccines utilized, as is required when the information is to be used to support decisions with global significance.

In ideal phase 3 clinical trials, where all the enrolled individuals are monitored during the same number of days, the E value is calculated considering the ratio between the relative incidence of the confirmed cases in the vaccinated group (${RI}^{V}$) and the equivalent relative incidence in the unvaccinated group (${RI}^{U}$). This relation is described in Equation (1), where each relative incidence (${RI}^{V}$ or ${RI}^{U}$) is expressed as the number of confirmed cases (${CC}^{V}$ or ${CC}^{U}$) counted during the studied period for every 100,000 individuals in the respective group ($P^{V}$ or $P^{U}$ denote the total number of individuals in the respective group):

(1) E=1−RIVRIU=1−CCVPVCCUPU

In order to promote the obtainment of E values that show enough statistical robustness, it has been traditionally recommended that a total number of confirmed cases higher than a certain minimum value (${CC}^{T}={CC}^{V}+{CC}^{U}$) is considered [39]. In the case of COVID-19 vaccines, ${CC}^{T}$ values between 100 and 200 cases were usually considered [9,10,14,15]. However, some studies break this scheme by considering as few as 41 cases [13] or as many as around 800 cases [12]. This requirement is a factor that usually determines the duration of the study period to be considered in order to characterize such vaccines (period to count cases). In such studies, the period to count cases was usually around 100 days [9,10,11,13,14,15], but in one case, around twice this time was considered [12]. In addition, such a requirement demands the recruitment of many volunteers [39]. In fact, to study these vaccines, pharmaceutical companies recruited more than 10,000 individuals [9,10,11,12,13,14,15], although some even recruited around 40,000 [9,11].

During phase 3 clinical trials, it is usually assumed that the efficacy of any vaccine starts increasing shortly after it is administered, reaching a maximum E value, which is assumed to remain practically constant for a long time. Therefore, to avoid counting cases when the vaccine’s efficacy is not yet stable (condition required to use Equation (1)), the confirmed cases are counted after a settling period until the vaccines are considered to have already taken effect and reached a stable E value. For single-dose COVID-19 vaccines, the period to count cases started two or four weeks after vaccine administration [11]. However, for two-dose vaccines, the counting period started one [9,15] or two [10,12,13,14] weeks after the administration of the second-dose.

Unfortunately, in vaccines that have effectiveness values that wane over time (like the COVID-19 vaccines), a unique E value is not enough to describe the vaccine behavior; thus, a curve to describe the evolution of the vaccine’s effectiveness with the time from vaccination is required (continuous E profile). Additionally, since the vaccine effectiveness wanes over time (it does not have a stable period), it is incorrect to assume that the confirmed cases with different times from vaccination are equivalent. Unfortunately, as mentioned, in the estimations of the E value performed during the respective phase 3 clinical trials for COVID-19 vaccines, periods of around 100 days were usually used to count cases. Such a grouping implies that confirmed cases with different levels of effectiveness are considered to be equivalent, which is conceptually wrong. Thus, the global effectiveness values generated in this way are biased.

Since COVID-19 confirmed cases are usually reported every day on databases, it is possible to group individuals daily, highly reducing the bias mentioned above. Accordingly, to allow the estimation of the continuous E profile that describes the behavior of COVID-19 vaccines, it is required to modify the procedure traditionally used to apply Equation (1), avoiding using periods to count cases that are larger than one day. However, this requirement demands a significant increase in the number of individuals studied.

Considering this, this study aims to propose a generic data processing strategy that can easily be used to estimate unbiasedly and with high precision the continuous E profile that characterizes the set of vaccines administered to each studied population group. Since it is desirable that a broad sector of health professionals can easily use this strategy, the calculation procedure was designed to be implemented in a widely known software. In addition, a suitably preconfigured worksheet is provided as supplementary material, as well as a user’s guide describing, step by step, the use of such a worksheet.

The proposed strategy requires information that is usually available in massive COVID-19 databases (e.g., the symptoms onset date of each confirmed case registered), as well as the number of individuals vaccinated each day in the interest group (the individual vaccination status is not required, which for privacy reasons, may not be available).

The proposed methodology was designed to provide a continuous profile (a curve on which the effectiveness changes its value daily) instead of a single value (as performed by the traditional clinical trials that assume that the value remains constant for a given time). In addition, since this work’s methodology does not need to group the confirmed cases with very different times since vaccination as equivalents, as usually has been required in the previous studies, the involved bias is reduced in this work. Additionally, due to the proposed methodology requiring a high number of confirmed cases (usually around 1000 times higher than in traditional studies) compared to the previous methodologies, it provides increased statistical robustness to the results, shortens the confidence intervals, and facilitates statistical comparison among the different population groups.

Regarding the practical utility, the availability of continuous E profiles would now allow the establishment of the date on which the daily value of the respective E profile decreases to an unacceptably low value for each considered population group, making the administration of boosters necessary for the respective group(s). Additionally, since the proposed methodology allows the obtainment of one E profile for each of the different population groups, the authorities could now optimize resources, and select the population group(s) for whom the available vaccines would provide more benefits when aiming to control the pandemic; this takes into account the fact that the benefits induced by a vaccine expectably depend on the characteristics of the population group (e.g., age, sex, race, or comorbidities).

## 2. Methods

### 2.1. Relative Incidence of Confirmed Total Cases for the Interest Population Groups

As the first step in implementing the data processing strategy that is proposed in this work, knowing the relative incidence of the total confirmed cases for the interest population groups (${RI}^{T}$) is required, that is, the respective daily values for the following quotient: (CCV+CCU)(PV+PU)=RIT. Such values must have been evaluated for a period as long as possible, including a time interval in which the whole group remains unvaccinated and a period in which the group is partially vaccinated. In the case of Mexicans, a period that started on 2 July 2020 and concluded on 23 September 2021 was selected; note that the vaccination process for the Mexicans grouped by age started on 15 February 2021.

**Figure 1:**
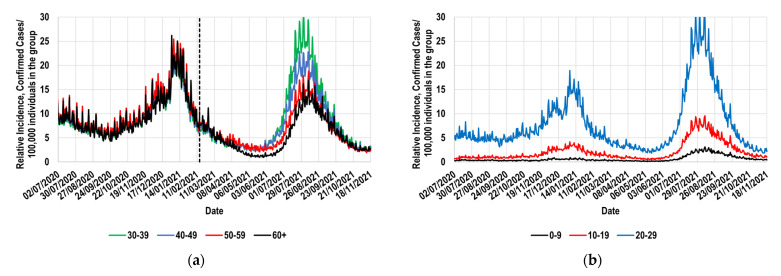
Relative incidence of confirmed cases by 100,000 individuals in the indicated group [41]. (**a**) Mexicans aged 30 years or older; (**b**) Mexicans aged 29 years or younger. Reference marker: Date at which the vaccination process of the 60+ group started (- - -) [44].

Regarding the total cases confirmed daily, this information can usually be obtained from the massive databases available in many countries (mDBs). In the case of Mexicans, this information was obtained from Mexico’s government COVID-19 database [40]. The information of such mDB was saved daily. Thus, to prevent the effect of some possible delay in the information transference during the register of confirmed cases, the file corresponding to 25 December 2021 was used [41]; note that the continuous *E* profiles calculated here only required information before 24 September 2021. The symptoms onset date of the first individual registered in such an mDB was 19 February 2020. This mDB contains information about 3,680,674 confirmed cases (cases declared by Mexico’s national government health system as positive COVID-19 cases) that showed onset symptoms before 24 September 2021. In such mDB, among other information, the age, sex, and some comorbidities of the confirmed cases were recorded, as well as their symptoms onset date and, when it applied, their hospitalization and mortality status. Thus, a Python script was developed in this work to collect the symptoms onset date of each confirmed case registered, classifying the individuals by their age and respective symptoms onset date (such a Python script, the public link to the used mDB, and the result of such a filtration process are included in Supplementary Material S1). The age categories considered are shown in Figure 1, which were selected to coincide with the vaccination groups considered in Mexico. In such a country, the vaccination process that was implemented was mainly regulated by age, starting with the 60+ group (60 or more years old), followed by the 50–59, 40–49, and 30–39 groups [42].

In most countries, the total populations of each age group are public. Therefore, the respective $P^{T}$ values ($P^{T}=P^{V}+P^{U}$) are usually available. For the example case, such values represent the number of Mexicans with their respective ages within the age categories considered in this work during 2020 [43]. Although a slight imprecision is involved, it was assumed that such a value remained constant during the studied period.

Thus, the ${RI}^{T}$ values that characterized the Mexicans during several months of the COVID-19 pandemic are presented in Figure 1 [41,43]. Such values are expressed as the number of confirmed cases showing the onset of symptoms on each specific date, measured by every 100,000 individuals in the specific population group. In Figure 1a, 63 million Mexicans aged 30 years or older are considered [43]. Complementarily, Figure 1b shows similar information for the younger 63 million Mexicans (0–29 years old [43]).

### 2.2. Identification of a Suitable Reference Group and Its Behavior during the Study Period

As a second step in the proposed procedure, it is required to identify a reference group (R group) classified by age as the 60+ group, that during a comparatively long period (e.g., some months), had remained unvaccinated like the studied group, showing both groups during such period, practically the same daily values for their respective ${RI}^{T}$ parameters (${RI}_{R}^{T}≅{RI}_{60+}^{T}$). In addition, the reference group must remain unvaccinated during the whole study period.

For this work’s example, such an identification must be made by analyzing the information presented in Figure 1. As a visual reference, Figure 1a includes a vertical line marking the date at which the vaccination process of the 60+ group started (15 February 2021 [44]). The 60+ group was the first set of Mexicans grouped by age that was vaccinated [42], followed by the 50–59 group, the 40–49 group, and then the others.

Figure 1a shows that all the curves overlap to the left of the vertical line, regardless of age. This behavior shows that, in the absence of vaccinations, all these population groups had practically the same daily values for their respective ${RI}^{T}$ profiles. This implies that, at least at this age range (30 or more years old), the probability of contagion was not perceptibly determined by age. Considering this, the 30–39, 40–49, and 50–59 groups could have been selected as the required reference group since their respective ${RI}^{T}$ values were practically indistinguishable. However, only the 30–39 group remained practically unvaccinated until the end of the study period (23 September 2021 [45]). Therefore, among the groups considered in Figure 1a, the 30–39 group was the best option to be assigned as the R group.

Figure 1b shows that among the younger Mexicans, as age increased, the respective ${RI}^{T}$ values became notably higher throughout the period considered. The different behavior shown by younger Mexicans (Figure 1b) could be at least partially attributed to the age-differentiated social isolation that was promoted in Mexico, which proved to be useful in reducing the respective ${RI}^{T}$ values (Figure 1b). Therefore, the proposed methodology cannot be used in these population groups. Thus, it can finally be affirmed that the 30–39 group is the best option to be used as the reference group for the elderly Mexicans; therefore, from now on, the following will be considered: ${RI}_{R}^{T}={RI}_{30-39}^{T}$.

Since the reference group remained completely unvaccinated during the entire study period, the unvaccinated fraction of the reference group and the totality of the group are identical. Therefore, the daily ${RI}_{30-39}^{T}$ values are identical to the daily ${RI}_{30-39}^{U}$ values (relative incidence of the unvaccinated members of the R group). In addition, as previously mentioned, when both groups remained completely unvaccinated, the 60+ group and the 30–39 group had, each day, values for their respective relative incidences that were practically indistinguishable themselves, that is, ${RI}_{30-39}^{U}≅{RI}_{60+}^{U}$. Finally, since the behavior of the unvaccinated individuals in the 60+ group was not altered by the vaccination process in such a populational group, the following equation ${RI}_{30-39}^{T}={RI}_{30-39}^{U}≅{RI}_{60+}^{U}$ is also valid when referring to the period in which the 60+ group was partially vaccinated and the 30–39 group remained unvaccinated.

### 2.3. The Beneficial Effect of Vaccines against Symptomatic Disease 

In Figure 1a, it can be observed that, some days after starting the vaccination process in the 60+ group, that is, some days after 15 February 2021 (to the right of the vertical line), the black curve gradually separates from the others, showing that the daily ${RI}^{T}$ values of this group were comparatively lower than those describing the behavior of the 50–59, 40–49, and 30–39 groups. This behavior was replicated when the other population groups considered in this figure started the process of being vaccinated. As previously mentioned, a vaccination process that was regulated mainly by age was implemented in Mexico, which advanced in the order of decreasing age [42]. Therefore, the last behavior can mostly be explained by considering that vaccination decreased the sensibility to contagion in the vaccinated individuals. Thus, the curves progressively get further apart as the vaccination processes advance in the respective population groups, representing the beneficial effect induced by them.

Thus, this work proposes an indicator that represents the beneficial effect that the vaccination process induced each day (during the date x) in the 60+ group ($B_{60+}$ profile), which can be calculated using Equation (2).

(2) $$B_{60+}^{date\;x}=1-\frac{{\left.{RI}_{60+}^{T}\right|}^{date\;x}}{{\left.{RI}_{60+}^{U}\right|}^{date\;x}}≅1-\frac{{\left.{RI}_{60+}^{T}\right|}^{date\;x}}{{\left.{RI}_{30-39}^{T}\right|}^{date\;x}}$$

According to Equation (2), the daily values of $B_{60+}^{date\;x}$ can be calculated using the daily values of the total relative incidences of the 60+ and 30–39 groups (${\left.{RI}_{60+}^{T}\right|}^{date\;x}$ and ${\left.{RI}_{30-39}^{T}\right|}^{date\;x}$ values), which are available in Figure 1. Note that the set of $B_{60+}^{date\;x}$ values becomes a profile ($B_{60+}$ profile), that is, a curve showing different values daily.

### 2.4. Vaccination Profile for the Interest Population Group

**Figure 2:**
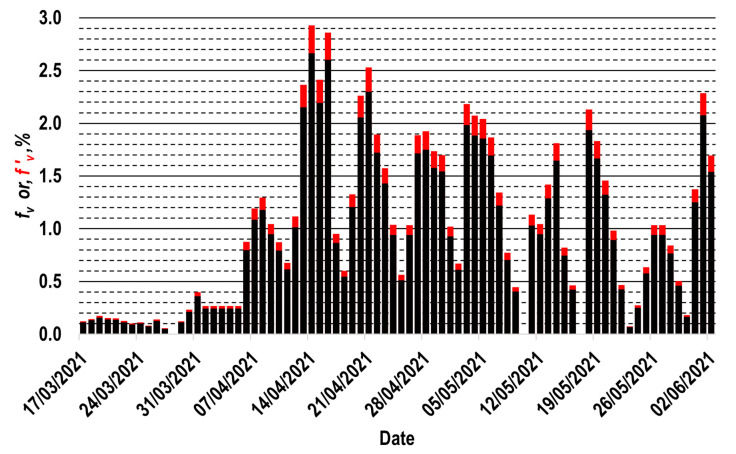
Vaccination profiles, referred to the completion date of the respective vaccination schemes. Profile considering officially published real-time information ($f_{v}$; black [44]), or assuming an underestimation of 10% in this profile (${f^{\prime }}_{v}$; red [44,46]).

Finally, the availability of the vaccination profile for the interest population group is required to apply the data processing strategy proposed in this work. Regarding the vaccination profile data for the 60+ group, they were collected from the information reported in real-time by the health secretary of Mexico, thus obtaining the n values of $f_{v}$, which are represented as black columns in Figure 2. Each $f_{v}$ value represents the percentage of individuals in the 60+ group that completed their respective vaccination schemes daily; the $f_{v}$ values have been defined so that the total population of the 60+ group represents 100%. It is important to mention that this is the only official public source that allows the vaccination progress of such a population group to be analyzed [44]. However, some months later, the same authority officially published the total number of daily doses applied during the first trimester of vaccination in Mexico [46], which is expectably more accurate information. Thus, the information published by both available references can be comparatively analyzed [44,46]. From such analysis, it can be concluded that, during that trimester, the $f_{v}$ values reported in real-time presented a slight underestimation, which was evaluated to be around 10% (some details about this estimation are presented in Supplementary Material S2).

In this circumstance, in order to estimate the effect of a possible underestimation of 10% in the number of individuals vaccinated each day, a second vaccination profile is shown in Figure 2. Such a profile is constituted by the n values of ${f^{\prime }}_{v}$, which are represented as red columns in this figure; each ${f^{\prime }}_{v}$ value was calculated as follows: ${f^{\prime }}_{v}={1.1f}_{v}$. Since practically all the individuals who requested to be vaccinated in this group were vaccinated within 78 days, both profiles are constituted by 78 values (n=78); note that the sum of all $f_{v}$ values is near 0.7, as only around 70% of the elderly Mexicans were vaccinated in this period.

### 2.5. The Relationship between the Vaccine Effectiveness Profile ($E_{60+}$ Profile), the Beneficial Effect Profile ($B_{60+}$ Profile) and the Vaccination Profile

As a first hypothetical case, let us consider that the 60+ group had only the $f_{1}$ percentage of its individuals completely vaccinated ($f_{1}=0.11\%$; first column in the histogram shown in Figure 2); that is, only 0.11% of the individuals in this group completed their respective vaccination schemes. For this hypothetical case, the following is algebraically demonstrable (the demonstration is presented in Supplementary Material S3):

(3) B60+date x=f1100E60+m

where $B_{60+}^{date\;x}$ is the beneficial effect that the vaccinated individuals in the 60+ group (0.11% of the group) induced in the complete group during the specific date x, whereas $E_{60+}^{m}$ is the effectiveness value that the vaccines administrated to such individuals had m days after completing their respective vaccination schemes. Note that m is the difference in days between the date x on which such a beneficial effect is evaluated and the first day that a vaccination scheme is completed in the 60+ group (17 March 2021), that is, m=date x−17 March 2021; to exemplify, when the date x is 1 April 2021, m=15 days. Additionally, as defined in Figure 2, $f_{1}$ is the percentage of the 60+ group that completed their vaccination schemes on 17 March 2021; that is, $f_{1}$ is the quotient between the number of individuals in the 60+ group that completed their vaccination schemes on this day and the total population of elderly Mexicans (15.1 million individuals [43]).

Let us now consider that in the 60+ group, only the $f_{1}$ and $f_{2}$ percentages completed their respective vaccination schemes (0.11% of the 60+ group completed their respective schemes on 17 March 2021, and the other 0.13% completed their respective ones on 18 March 2021; Figure 2). For this new hypothetical case, the beneficial effect produced in the global 60+ group can be obtained by adding the respective beneficial effects produced by each fraction of the 60+ group, as indicated in Equation (4) (additional details about the procedure for obtaining Equation (4) are presented in Supplementary Material S3).

(4) B60+date x=f1100E60+m+f2100E60+m−1

Observe that on the datex, 0.11% of the individuals in the 60+ group had m days of time from vaccination, but on this date, 0.13% of the individuals in the 60+ group ($f_{2}=0.13\%$; Figure 2) had only m−1 days since vaccination.

In the case of vaccines showing an effectiveness value that does not vary over time (as traditionally has been considered), $E_{60+}^{m}=E_{60+}^{m-1}$. Therefore, Equation (4) can be reduced to the following: $B_{60+}=\left(f_{1}+f_{2}\right)\left(E_{60+}\right)=f_{T}\left(E_{60+}\right)$, where $f_{T}$ is the total percentage of individuals vaccinated in the correspondent group. Thus, it can be affirmed that Equation (1) is a particular case of Equation (4) (and of its general form presented as Equation (5)), which is only valid when the effectiveness is maintained constant during the whole studied period; if such condition is not fulfilled, biased results will be produced. Thus, regardless of the $E_{60+}$ profile shape, with either a time-dependent curve or a horizontal line with a single value, Equation (5) can be used to estimate the $E_{60+}$ profile.

Since the 60+ group was vaccinated in 78 consecutive days, with each subgroup having completed its respective vaccination schemes in only one day, Equation (4) must be expanded as follows:

(5) B60+date x=f1100E60+m+f2100E60+m−1+f3100E60+m−2+⋯+f77100E60+m−76+f78100E60+m−77

as part of the proposed procedure, Equation (5) must be used several times, one time for each date x inside the period in which there are available values for the beneficial effect ($B_{60+}^{date\;x}$ values). Thus, Equation (5) represents a nonlinear system of equations that establishes the complex relationship among the $B_{60+}$ profile, the $E_{60+}$ profile, and the vaccination profile. The different $E_{60+}^{t}$ values cannot be calculated directly (t denotes the time from vaccination, specifically, the time since each individual completed his/her respective vaccination scheme and the date of interest). Therefore, they must be estimated through a systematic fitting process that is described, in detail, in Supplementary Material S4.

## 3. Results

### 3.1. The Resulting Beneficial Effect Profile ($B_{60+}$ Profile)

**Figure 3:**
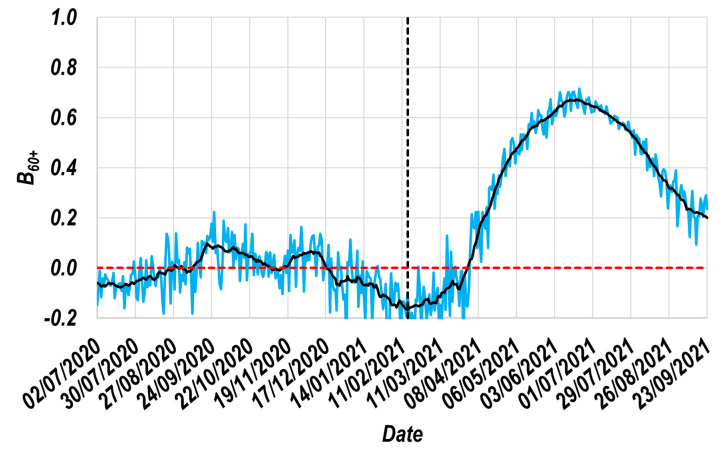
Beneficial effect induced by the vaccines administrated to Mexicans. Original data for 60+ group (___). Smoothed data (using a centered moving average of the 15^th^ order [47]) for 60+ group (___). Reference markers: Date at which the vaccination process of the 60+ group started (- - -) [44], and zero value (- - -).

The daily $B_{60+}^{date\;x}$ values obtained through Equation (2) (Section 2.3) and the daily values of ${RI}_{60+}^{T}$ and ${RI}_{30-39}^{T}$ are presented in Figure 3 (blue line). However, since the curve shown in blue has high statistical variability, a centered moving average of 15th order was used to improve the observability of its trend [47], allowing its smoothing without affecting its original trend. Thus, such a curve after applying this smoothing procedure is presented in black; note that this curve was used to estimate the $E_{60+}$ profile.

In this curve, it can be observed that there was a very long period (>37 weeks) in which the daily $B_{60+}^{date\;x}$ values were practically zero (most of them between −0.1 and 0.1), which quantitatively shows that the daily values of ${RI}_{60+}^{T}$ and ${RI}_{30-39}^{T}$ are practically identical (requirement to assign to the R group). Additionally, the proximity between the dates on which such a profile begins a sharp increment and the date on which the vaccination of the 60+ group started (vertical line in Figure 3 [44]) supports the hypothesis that the susceptibility of the group to contagion decreases progressively as the vaccination process advances.

Unfortunately, after mid-June, the values of the beneficial effect profile show an opposite trend, decreasing as time advances. On this subject, it is relevant to consider that around June 2, the vaccination process in the 60+ group had practically concluded, with around 70% of the total population of this group having been vaccinated up to that date [44]. The latter behavior is consistent with the fact that the vaccine progressively decreases its effectiveness over time, which represents valuable knowledge that could be used to anticipate the need for boosters for this population group. Equivalent behaviors have been reported in other population groups [19,20].

### 3.2. The Resulting Vaccine Effectiveness Profile ($E_{60+}$ Profile)

**Figure 4:**
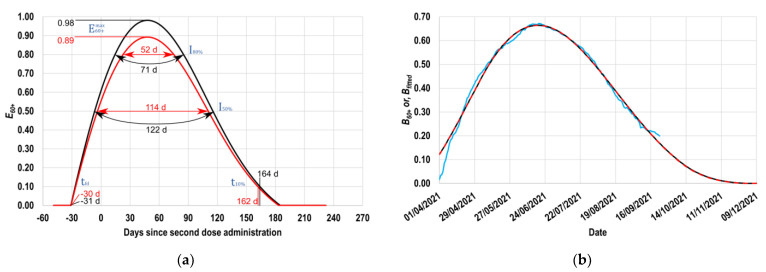
Results obtained with the proposed methodology, considering the officially published real-time vaccination profile (black curves) or assuming an underestimation of 10% in such a profile (red curves). (**a**) $E_{60+}$ profiles. (**b**) Real-world $B_{60+}$ smooth profile (blue curve) and fitted profiles ($B_{fitted}$).

As a result of the application of the here proposed systematic fitting process, continuous $E_{60+}$ profiles are obtained (Figure 4a). However, for some uses, it is preferable to have available a simplified description of the resulting $E_{60+}$ profile. For such cases, the following representative parameters of such a profile have been defined (Figure 4a):

- Maximum value of the $E_{60+}$ profile ($E_{60+}^{max}$ value);
- Number of days prior to the application of the second dose during which the first dose effects are perceptible in the group ($t_{fd}$);
- Time interval during which the daily value of $E_{60+}$ is greater than 0.8 ($I_{80\%}$);
- Time interval during which the daily value of $E_{60+}$ is greater than 0.5 ($I_{50\%}$);
- Minimum number of days after completing the vaccination scheme required for the daily value of $E_{60+}$ to decrease to values less than 0.1 ($t_{10\%}$).

The adequate application of the procedure described in Supplementary Material S4, through the use of a preconfigured Excel spreadsheet (Supplementary Material S5) and its user guide (Supplementary Material S6) allowed the obtainment of the $E_{60+}$ profiles presented in Figure 4a. The black curve shown in this figure was calculated considering the $f_{v}$ histogram, whereas the red one represents the $E_{60+}$ profile estimated considering a possible underestimation of the number of vaccinated individuals (${f^{\prime }}_{v}$ histogram). Taking into account the mentioned in Section 2.4., it is expected that the real $E_{60+}$ profile is located between the black and red profiles shown in Figure 4a.

Regarding the $E_{60+}$ profile estimated from the vaccination profile reported in real-time ($f_{v}$ histogram; black curve in Figure 4a), it must be highlighted that its maximum value is practically unitary ($E_{60+}^{max}=0.98$; Figure 4a), demonstrating the very high maximum effectiveness against the symptomatic disease of vaccines used in this population group. In addition, the obtained results allow this study to affirm that a favorable immune response with populationally detectable effects started around 31 days before applying the second dose ($t_{fd}$ value). This value shows a qualitative agreement with the difference in days (30 days) between the beginning of the application of the first doses (15 February 2021) and the second doses (17 March 2021) in this population group [44]. The last demonstrates that the immunological effects are globally detectable very shortly after the application of the first dose.

Concerning the $I_{80\%}$ value represented in Figure 4a (the time interval during which the daily value of $E_{60+}$ is greater than 0.8), the obtained results estimate that for 71 days, a robust immune response is induced in the vaccinated population ($E_{60+}$ > 0.8). Equivalently, the $I_{50\%}$ value (Figure 4a) suggests that for 122 days, the $E_{60+}$ value could be considered acceptable (>50%) [48]. Finally, the value of $t_{10\%}$ (number of days after the completion of the vaccination scheme required for the $E_{60+}$ profile to show values smaller than 0.1) indicates that five and a half months after applying the second dose, the vaccine’s immunological benefits could already be considered marginal.

Complementarily, analyzing the $E_{60+}$ profile calculated considering the ${f^{\prime }}_{v}$ histogram (red curve in Figure 4a), it can be observed that the value of $E_{60+}^{max}$ is 0.89. Therefore, it is realistic to expect that the value of $E_{60+}^{max}$, which characterizes the set of vaccines applied in Mexico to the 60+ group, is higher than 0.89; in most members of this population group, the BNT162b2 or AZD1222 vaccines were administered. In addition, comparatively analyzing the behavior of the parameters $I_{80\%}$ and $I_{50\%}$, one can observe that when a possible underestimation in the number of vaccinated individuals is considered (by using the ${f^{\prime }}_{v}$ histogram), the respective protection intervals decrease slightly (for $I_{80\%}$, from 71 to 52 days, and for $I_{50\%}$ from 122 to 114 days). Therefore, it is valid to indicate that the real value of $I_{80\%}$ is approximately two months, while $I_{50\%}$ is approximately four months. 

Finally, by focusing on the parameter $t_{10\%}$, it is possible to observe that the possible underestimation in the values of $f_{v}$ does not significantly impact this parameter, which remains close to 6 months in both cases (162 vs. 164 days).

To evidence the excellent level of fitting achieved by this methodology, in Figure 4b, the following profiles are presented: (a) real-world $B_{60+}$ smooth profile (in blue; reproduced from Figure 3), (b) $B_{60+}$ profile calculated with $f_{v}$ data (in black), and (c) $B_{60+}$ profile calculated with ${f^{\prime }}_{v}$ data (in red).

Figure 4b shows the high similarity between the real-world information (profile in blue) and the respective fitting curves (black and red profiles). Quantitatively speaking, the root of the normalized mean square deviation of both profiles is less than 3% concerning the real data, which validates the applicability of the proposed methodology for the example case.

### 3.3. Comparison with Previous Reports

**Figure 5:**
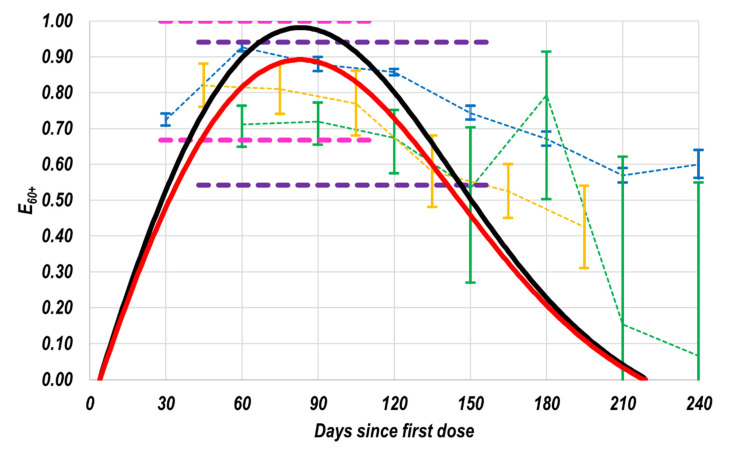
Comparison of the results of this study against previously reported information. Proposed $E_{60+}$ profiles (considering the $f_{v}$ (___) or the ${f^{\prime }}_{v}$ (___) profiles). 95% CIs for the interest group published by the respective manufacturers for the BNT162b2 (- - -) [9] and AZD1222 (- - -) [10] vaccines. Set of E values as a function of the time from vaccination for the BNT162b2 vaccine (___) [16], (___) [17], and (___) [18].

For comparison, in Figure 5, the proposed profiles (reproduced from Figure 4a) and some previously reported E values are shown; their respective 95% confidence intervals (CIs) are also included. It is essential to mention that, since practically the total population of the interest groups was considered in this work (15.1 million for the 60+ group and 18.4 million for the 30–39 group), the 95% CIs of the here proposed profiles are imperceptible in Figure 5. The 95% CIs for the single E values estimated in the original phase 3 clinical trials for the vaccines BNT162b2 [9] and AZD1222 [10] are shown as dotted lines in Figure 5 (pink and purple, respectively). Since the minimum period in which each manufacturer’s data could be valid correspond to their respective period to count cases, such dotted lines were graphically extended to cover such periods, that is, 28 to 111 days since the first dose for the BNT162b2 vaccine [9], and 43 to 158 days for the AZD1222 vaccine [10].

Additionally, some available 95% CIs for the effectiveness as a function of the time from vaccination, published by different research groups for the BNT162b2 vaccine, are also presented in Figure 5 (blue [16], green [17], or yellow [18]). Finally, only to ease the visualization, slim dotted lines join the isolated monthly values reported in each study.

A comparison among the three available evolutions over time (blue [16], green [17], or yellow [18]), and the confidence interval for the E value reported by the BNT162b2 vaccine’s manufacturer [9], shows that there is a qualitative agreement among them during the period to count cases of the original phase 3 clinical trial, that is, between 28 and 111 days after the first dose. Nevertheless, the qualitative agreement among such evolutions over time [16,17,18] demonstrates that effectiveness wanes after a certain $E_{60+}^{max}$ value is reached, which was not explicitly mentioned in the original phase 3 clinical trial [9].

On the other hand, the slight or moderate differences among such evolutions over time [16,17,18] can be explained, among other reasons, due to the different proportions of the variants that were circulating during the respective studies and the slightly different age categories considered in them (in two of them [16,18], the studied group is the 65+ group, while in the other one [17], the studied group was the 60+ group).

Now, comparing the lines in black or red (this work’s results) with the areas framed with thick dotted lines (95% CIs published by the vaccines’ manufacturers), it is possible to note in Figure 5 that, qualitatively speaking, both results are in agreement. However, from the quantitative point of view, the superiority of this work’s results is evident, presenting a continuous E profile, which shows a significantly higher precision than the original E values. It is worth noting that, in this work, practically all the interest population is considered, not only a sample of them. Therefore, the confidence intervals of the results of this work are imperceptible in Figure 5. Moreover, when the here presented novel approach is used, the bias arising from accumulating cases over a long period is practically avoided; as mentioned, Equation (1) was not designed to be used in such a way.

Focusing now on the time evolution of the proposed $E_{60+}$ profiles (black and red curves), they show a qualitative agreement with the previously available results [16,17,18]; indeed, all of them wane in effectiveness as the time from vaccination increases. However, as in the previous analysis, the statistical superiority of the actual proposal is evident (practically unbiased and considerably more precise values are produced). It is also worth noting that this approach produces a continuous smooth profile instead of the traditional isolated discrete values. This type of result is noticeably more useful for designing, by mathematical modeling, improved public health strategies, which can increase their positive impact on the contention of the local and global pandemic.

## 4. Discussion

Until now, vaccines have been useful in containing the pandemic partially, reaching very different success levels among the different countries. However, its persistence for more than three years [49], coupled with the inability to provide the required vaccines to the entire humanity timely [25] (several times per year), makes it essential to seek improved vaccination schedules to maximize the mitigation of the pandemic with the available vaccines at both levels, local and global. Nevertheless, the availability of the E profiles that characterize each involved population group with precision and without bias is an indispensable requirement for designing such improved vaccination schedules.

As mentioned, some efforts have been made on this issue by evaluating the vaccine effectiveness, considering the relative incidences in different periods during the COVID-19 pandemic [16,17,18]. In such works, the requirement to collect enough confirmed cases in order to guarantee suitable statistic robustness (e.g., to shorten the confidence intervals) forced the authors to accumulate the confirmed cases during numerous days, only allowing them to estimate monthly E values. Regardless of such a limitation, their results qualitatively demonstrate that the vaccine’s effectiveness wanes as the time since vaccination increases, which is in qualitative agreement with this work’s results. Unfortunately, each value obtained by this methodology [16,17,18] was calculated by grouping individuals with different times since vaccination as equivalent, which is methodologically incorrect and generates biased results. Therefore, it is expected that their monthly values have a certain level of inaccuracy, which motivated us to seek an alternative methodology that does not involve such methodologic bias.

The present study considers a number of individuals that is considerably higher than that used in the previous studies [16,17,18] (this study counted 512,660 confirmed cases in the 60+ group), avoiding considering confirmed cases as equivalents when they had very different times since vaccination. Therefore, the obtained $E_{60+}$ profile reduced the bias produced by the traditional procedure. In addition, since practically all the individuals in the involved population groups were considered, the confidence interval length is practically negligible. Finally, as a consequence of the huge number of confirmed cases counted, even while studying the population subgroups classified by age, sex, race, or comorbidities, the results’ statistical robustness could be assured. Thus, the proposed methodology allows the obtainment of continuous *E* profiles, showing a lower level of bias and higher precision than previous methodologies.

However, to obtain the advantages mentioned above, the following two requirements must be fulfilled:

(1) The availability of the mDBs containing realistic information about the symptom onset dates of many thousands of confirmed cases (for this work’s example, more than half a million confirmed cases were considered), as well as the number of individuals vaccinated each day in the study group. It is essential to recognize that the obtainable results are as realistic as the used information. In Mexico, during the study period, the COVID-19 tests, the registration of confirmed cases, and the vaccination campaigns were entirely controlled by the country’s leading health authorities; this could favor minor or slight imprecisions in data.
(2) The assignation of a reference group; for this study, the 30–39 group was selected. During a long period, the reference group must have exhibited daily values of its relative incidence that were similar to those shown by the study group. Figure 1a and Figure 3 demonstrate that the Mexican 30–39 group fulfilled such a condition for at least 37 weeks. It would be expected that population groups in which the recommendation to socially isolate was successfully implemented would not fulfill this condition. Therefore, its fulfillment must not be assumed, and it must be corroborated with real-world data. To illustrate these exceptions, consider the case of the Mexican students, who continued their education remotely during the study period. This strategy is expected to have significantly contributed to the decrease in relative incidence among younger Mexicans (<30 years old). Thus, the proposed methodology must not be applied to such population groups.

The last two requirements were probably not fulfilled in all countries during the COVID-19 pandemic. However, it is likely that such conditions were fulfilled in several of them. In addition, it is likely that the proposed methodology’s availability would help to increase the world’s interest in generating suitable mDBs and professionalizing the process of uploading data to them, which would be expected to increase the number of countries fulfilling the first requirement.

When the conditions allow its use, using the proposed data processing strategy is a good option in order to estimate, with high precision and practically without bias, the E profile that characterizes the set of vaccines administered to a given population group. Nevertheless, as previously mentioned, it must be recognized that the results obtained using the proposed methodology are as realistic as the used mDBs are correct and unbiased, resulting in a strong motivation to systematize the massive collection of data regarding emerging diseases. Despite the utility of previous studies to qualitatively show the waning of the vaccine’s effectiveness, their discrete E values [16,17,18] are not a formal continuous profile and cannot be used to successfully design vaccination schedules that considerably improve the control of the pandemic with the available vaccines. Conversely, local health authorities can now use the continuous E profiles that are obtainable by this novel approach to select, considering real-world information, the most opportune moment to apply boosters to each local population group. Such selection includes considering that the magnitude of the benefit inducible by vaccines could vary considerably among the population groups.

In addition, the integral analysis of such profiles would allow the daily identification of the population group(s) in which the available vaccines would highly increase the expected benefits (e.g., minimization of confirmed COVID-19 cases).

This methodology could now be used to characterize the set of COVID-19 vaccines administered in any region or country with the required historical information available. In addition, it could also be used to evaluate the behavior of any vaccine developed in the future, especially those showing time-dependent effectiveness. In fact, when possible, it would be recommendable to use this methodology periodically to actualize, with precision and accuracy, the vaccine’s effectiveness values, which have been previously measured for the population groups of interest, but considering the currently circulating variants.

Once the mDBs have been implemented, and the required computational tool is available (Supplementary Materials S4 and S5), the cost, the efforts, and the time required to implement our proposal are negligible compared to those involved during traditional phase 3 clinical trials, allowing actualizations to be performed as frequently as required. Such actualizations are useful to monitor and quickly identify the need for making modifications to the previous versions of the vaccines, aiming for an improvement in their response against emerging variants, all without recruiting big testing groups and spending time on long studies.

Regarding the studied populational group, the presented results demonstrate that in elderly Mexicans, the global effectiveness of the vaccines used in this population group is acceptable (>0.50 [48]) only for approximately four months. Thus, applying several vaccine doses per year would be required to suitably control the contagion in this population group, which is precious practical information. However, it should be noted that this protection level could be modified with the appearance of new SARS-CoV-2 variants in the population. Therefore, as previously mentioned, periodic evaluations with this methodology would be necessary for successful monitoring.

As an additional contribution, the proposed methodology can be adapted to estimate the vaccine effectiveness against hospitalization and mortality. This information would improve the characterization of the expected benefits of the set of vaccines administered to each population group, allowing the optimization of other global objectives (e.g., minimizing hospitalization or death rates).

Finally, the proposed methodology represents an advance concerning the currently available methodologies. However, it must be recognized that massive databases containing information about millions of individuals must be available to apply this methodology successfully. In addition, such information must unbiasedly represent real-world conditions. Concerning this requirement, the availability of national or even international databases would allow this methodology to offer high precision and accuracy, now for the characterization of COVID-19 vaccines, but in the future, to characterize other diseases’ vaccines.

## 5. Conclusions

The proposed methodology allows precise and accurate estimations of the effectiveness profiles that characterize each population group, allowing the effect of age, sex, race, or comorbidities to be considered, while maintaining statistical robustness. This knowledge is valuable for designing vaccination schedules that increase the global benefits of vaccination campaigns implemented in different countries. Due to its generality, the impact of the proposed methodology is not limited to the COVID-19 pandemic. It can also be used to analyze any other disease for which vaccines with time-dependent effectiveness have been developed.

## Data Availability

The basic data used for performing this study (e.g., the mDB corresponding to 25 December 2021) are available in the links referenced in this article’s main text or the files included in its Supplementary Materials.

## Supplementary Materials

The following supporting information can be downloaded at: https://www.mdpi.com/article/10.3390/vaccines11040719/s1, Supplementary Material S1: File: Code.py: Phyton script used for filtration process; File: 211225COVID19MEXICO.pdf: The public link to obtain the massive database used to count and classify the confirmed cases; File: Results.xlsx: Result of the filtration process used to count and classify the confirmed cases. File: README.txt: Relevant observations that should be considered before trying to read the massive database, Supplementary Material S2: Details about the vaccination process: An approximation of the number of vaccines administered daily, Supplementary Material S3: Algebraic demonstrations of the mathematical relationship among the $B_{60+}$ profile, the $E_{60+}$ profile, and the vaccination profile for two example cases, Supplementary Material S4: Details regarding the systematic fitting process used to estimate the $E_{60+}$ profile (References [50,51,52,53] are cited in Supplementary Materials), Supplementary Material S5: Excel spreadsheet preconfigured to perform the systematic fitting process required to estimate the $E_{60+}$ profile, Supplementary Material S6: A user guide for utilizing the Excel spreadsheet included in Supplementary Material S5, Supplementary Material S7: Excel spreadsheet containing example hypothetical profiles required to practice with the preconfigured Excel spreadsheet included in Supplementary Material S5.

## Abbreviations

WHO	World Health Organization
E	Vaccine effectiveness
B	Beneficial effect induced by the administrated vaccines
$f_{v}$	Percentage of the population daily vaccinated (registered in real-time)
${f^{\prime }}_{v}$	Percentage of the population daily vaccinated (considering a possible underestimation of 10% concerning the $f_{v}$ values)
RI	Relative Incidence
${}^{V}$	Vaccinated group (expressed as a superscript)
${}^{U}$	Unvaccinated group (expressed as a superscript)
${}^{T}$	Totality of a group (expressed as a superscript)
CC	Confirmed Cases
P	Population of a group
mDB	massive Database
R	Reference group
${}_{+60}$	Group of 60 or more years old (expressed as a subscript)
${}_{R}$	Reference group (expressed as a subscript)
${}_{30-39}$	Group of 30 to 39 years old (expressed as a subscript)
${}^{date\;x}$	A specific date for which an affirmation is valid (expressed as a superscript)
${}^{t}$	time elapsed since the completion of the vaccination scheme (expressed as a superscript)
$E^{max}$	Maximum value of the E profile
$t_{fd}$	Number of days prior to the application of the second dose during which the first dose effects are perceptible in the group
$I_{80\%}$	Time interval during which the daily value of E is greater than 0.8
$I_{50\%}$	Time interval during which the daily value of E is greater than 0.5
$t_{10\%}$	Minimum number of days after completing the vaccination scheme required for the daily value of E to decrease to values less than 0.1.
CI	Confidence Interval
