# Supplementary material for: A Novel Approach to Obtain Vaccine Effectiveness Continuous Profiles. Example Case: COVID-19 in Elderly Mexicans
Source: Vaccines (Basel). 2023 Mar 23;11(4):719. doi: 10.3390/vaccines11040719 (PMC10142991; doi:10.3390/vaccines11040719)
Supplement: Supplementary file 1 [file vaccines-11-00719-s001.zip › Supplementary Material 1/211225COVID19MEXICO.pdf]

The file named 211225COVID19MEXICO.csv, containing the COVID-19 massive database saved on December 25, 2021, by Mexico's National Health authorities, which was used to count and classify the confirmed cases, is available at: https://datosabiertos.salud.gob.mx/gobmx/salud/datos_abiertos/historicos/2021/12/datos_abiertos_covid19_25.12.2021.zip
