# Supplementary material for: A Novel Approach to Obtain Vaccine Effectiveness Continuous Profiles. Example Case: COVID-19 in Elderly Mexicans
Source: Vaccines (Basel). 2023 Mar 23;11(4):719. doi: 10.3390/vaccines11040719 (PMC10142991; doi:10.3390/vaccines11040719)
Supplement: Supplementary file 1 [file vaccines-11-00719-s001.zip › Supplementary Material 3.pdf]

DEMONSTRATION 1

Let us consider that in the $date\ x$, the 60+ group is only constituted for $P_{60+}^{V}|^{date\ x}$ individuals that completed their respective vaccination schemes on the $date\ j$ (in this system, the only vaccination date was the $date\ j$, therefore, $P_{60+}^{V}|^{date\ j+1} = P_{60+}^{V}|^{date\ x}$), and $P_{60+}^{U}|^{date\ x}$ Unvaccinated individuals (as in the previous case, $P_{60+}^{U}|^{date\ j+1} = P_{60+}^{U}|^{date\ x}$). Although it implies a slight imprecision, it was considered that the total population in the 60+ group remained constant during the studied period.

For this case, it is desirable to demonstrate the validity of Eq. 3 of the manuscript. Thus, the demonstration starts with the following equation:

$$B_{60+}^{date\ x} = \left(f_1/_{100}\right)\left(E_{60+}^{m}\right) \tag{SM3-1}$$

where $B_{60+}^{date\ x}$ is the Beneficial effect that the vaccinated individuals in the 60+ group induced in the global 60+ group during the specific $date\ x$, whereas E_{60+}^{m} is the effectiveness value that the vaccines administrated to such individuals had m days after completing their respective vaccination schemes. Note that m is the difference in days between the $date\ x$ in which the $B_{60+}^{date\ x}$ value is being evaluated and the first day of completion of vaccination schemes of the 60+ group (for this case, March 17, 2021), that is, $m = date\ x - March\ 17, 2021$. Besides, it must be considered that

$$f_1 = \left(\frac{P_{60+}^{V}|^{date\ j+1}}{P_{60+}^{V}|^{date\ j+1} + P_{60+}^{U}|^{date\ j+1}}\right)(100) = \left(\frac{P_{60+}^{V}|^{date\ x}}{P_{60+}^{V}|^{date\ x} + P_{60+}^{U}|^{date\ x}}\right)(100).$$

On the other hand, adapting the equation traditionally used to calculate the vaccine effectiveness (Eq. 1 of the manuscript) for a vaccine that shows a time-dependent E_{60+} profile, it is possible to establish for this new case the following multiple-relationship:

$$E_{60+}^{m} = 1 - \frac{RI_{60+}^{V}|^{date\ x}}{RI_{60+}^{U}|^{date\ x}} = 1 - \frac{\left(\frac{CC_{60+}^{V}|^{date\ x}}{P_{60+}^{V}|^{date\ x}}\right)}{\left(\frac{CC_{60+}^{U}|^{date\ x}}{P_{60+}^{U}|^{date\ x}}\right)} \tag{SM3-2}$$

Note that Eq. SM3-2 is a particular case of Eq. 1 of the manuscript, where all the Confirmed Cases ($CC_{60+}^{V}|^{date\ x}$ or $CC_{60+}^{U}|^{date\ x}$) were collected in only one day (specifically on the $date\ x$), and m is the time from vaccination, that is, $m = date\ x - date\ j$. Besides, as mentioned in the manuscript, the Relative Incidences ($RI_{60+}^{V}|^{date\ x}$ or $RI_{60+}^{U}|^{date\ x}$) have been expressed as the number of confirmed cases counted in the $date\ x$ for every 100 thousand individuals of the respective group (Vaccinated or Unvaccinated individuals in the 60+ group).

Substituting the last two expressions on Equation SM3-1, the following relationship can be established: $B_{60+}^{date\ x} = \frac{P_{60+}^{V}|^{date\ x}}{P_{60+}^{V}|^{date\ x} + P_{60+}^{U}|^{date\ x}}\left(1 - \frac{\left(\frac{CC_{60+}^{V}|^{date\ x}}{P_{60+}^{V}|^{date\ x}}\right)}{\left(\frac{CC_{60+}^{U}|^{date\ x}}{P_{60+}^{U}|^{date\ x}}\right)}\right)$. However, this equation can be reordered as follows: $B_{60+}^{date\ x} = \frac{P_{60+}^{V}|^{date\ x}}{P_{60+}^{V}|^{date\ x} + P_{60+}^{U}|^{date\ x}} - \frac{\left(\frac{CC_{60+}^{V}|^{date\ x}}{P_{60+}^{V}|^{date\ x} + P_{60+}^{U}|^{date\ x}}\right)}{\left(\frac{CC_{60+}^{U}|^{date\ x}}{P_{60+}^{U}|^{date\ x}}\right)}$. Thus, considering now that

$$\frac{P_{60+}^{V}\big|^{date\,x}}{P_{60+}^{V}\big|^{date\,x}+P_{60+}^{U}\big|^{date\,x}} = \frac{P_{60+}^{V}\big|^{date\,x}+P_{60+}^{U}\big|^{date\,x}-P_{60+}^{U}\big|^{date\,x}}{P_{60+}^{V}\big|^{date\,x}+P_{60+}^{U}\big|^{date\,x}} = 1-\frac{P_{60+}^{U}\big|^{date\,x}}{P_{60+}^{V}\big|^{date\,x}+P_{60+}^{U}\big|^{date\,x}}$$, and that

$$\frac{\left(\frac{CC_{60+}^{V}\big|^{date\,x}}{P_{60+}^{V}\big|^{date\,x}+P_{60+}^{U}\big|^{date\,x}}\right)}{\left(\frac{CC_{60+}^{U}\big|^{date\,x}}{P_{60+}^{U}\big|^{date\,x}}\right)} = \frac{\left(\frac{CC_{60+}^{V}\big|^{date\,x}}{CC_{60+}^{U}\big|^{date\,x}}\right)}{\left(\frac{P_{60+}^{V}\big|^{date\,x}+P_{60+}^{U}\big|^{date\,x}}{P_{60+}^{U}\big|^{date\,x}}\right)} = \left(\frac{CC_{60+}^{V}\big|^{date\,x}}{CC_{60+}^{U}\big|^{date\,x}}\right)\left(\frac{P_{60+}^{U}\big|^{date\,x}}{P_{60+}^{V}\big|^{date\,x}+P_{60+}^{U}\big|^{date\,x}}\right)$$

The following equation can be proposed:

$$B_{60+}^{date\,x} = 1-\frac{P_{60+}^{U}\big|^{date\,x}}{P_{60+}^{V}\big|^{date\,x}+P_{60+}^{U}\big|^{date\,x}}-\left(\frac{CC_{60+}^{V}\big|^{date\,x}}{CC_{60+}^{U}\big|^{date\,x}}\right)\left(\frac{P_{60+}^{U}\big|^{date\,x}}{P_{60+}^{V}\big|^{date\,x}+P_{60+}^{U}\big|^{date\,x}}\right)$$
$$= 1-\left(1+\left(\frac{CC_{60+}^{V}\big|^{date\,x}}{CC_{60+}^{U}\big|^{date\,x}}\right)\right)\left(\frac{P_{60+}^{U}\big|^{date\,x}}{P_{60+}^{V}\big|^{date\,x}+P_{60+}^{U}\big|^{date\,x}}\right)$$

Alternatively, it is possible to reorder the previous equation as follows:

$$B_{60+}^{date\,x} = 1-\left(\frac{CC_{60+}^{U}\big|^{date\,x}+CC_{60+}^{V}\big|^{date\,x}}{CC_{60+}^{U}\big|^{date\,x}}\right)\left(\frac{P_{60+}^{U}\big|^{date\,x}}{P_{60+}^{V}\big|^{date\,x}+P_{60+}^{U}\big|^{date\,x}}\right) =$$

$$1-\frac{\left(\frac{CC_{60+}^{U}\big|^{date\,x}+CC_{60+}^{V}\big|^{date\,x}}{CC_{60+}^{U}\big|^{date\,x}}\right)}{\left(\frac{P_{60+}^{V}\big|^{date\,x}+P_{60+}^{U}\big|^{date\,x}}{P_{60+}^{U}\big|^{date\,x}}\right)} = 1-\frac{\frac{CC_{60+}^{U}\big|^{date\,x}+CC_{60+}^{V}\big|^{date\,x}}{P_{60+}^{V}\big|^{date\,x}+P_{60+}^{U}\big|^{date\,x}}}{\frac{CC_{60+}^{U}\big|^{date\,x}}{P_{60+}^{U}\big|^{date\,x}}} \quad \text{(SM3-3)}$$

In Eq. SM3-3, it can be identified that $\left(\frac{CC_{60+}^{U}\big|^{date\,x}+CC_{60+}^{V}\big|^{date\,x}}{P_{60+}^{V}\big|^{date\,x}+P_{60+}^{U}\big|^{date\,x}}\right) = \frac{RI_{60+}^{T}\big|^{date\,x}}{100000}$ (total relative incidence in the 60+ group, which is expressed as the total number of confirmed cases in the $date\ x$ in the 60+ group per every 100,000 individuals in the such group), and $\left(\frac{CC_{60+}^{U}\big|^{date\,x}}{P_{60+}^{U}\big|^{date\,x}}\right) = \frac{RI_{60+}^{U}\big|^{date\,x}}{100000}$.

Thus, the following expression can be obtained:

$$B_{60+}^{date\,x} = 1-\frac{RI_{60+}^{T}\big|^{date\,x}}{RI_{60+}^{U}\big|^{date\,x}} \quad \text{(SM3-4)}$$

Besides, in the manuscript was demonstrated that $RI_{60+}^{U}\big|^{date\,x} \cong RI_{30-39}^{U}\big|^{date\,x}$ (relative incidence of confirmed cases in the $date\ x$ in the 30-39 group, which is constituted only by unvaccinated individuals). Therefore, the following equation can also be proposed as valid:

$$B_{60+}^{date\,x} \cong 1-\frac{RI_{60+}^{T}\big|^{date\,x}}{RI_{30-39}^{U}\big|^{date\,x}} \quad \text{(SM3-5)}$$

Note that Eq. SM3-4 and Eq. SM3-5 constitute the Eq. 2 of the manuscript, that is

$$B_{60+}^{date\,x} = 1-\frac{RI_{60+}^{T}\big|^{date\,x}}{RI_{60+}^{U}\big|^{date\,x}} \cong 1-\frac{RI_{60+}^{T}\big|^{date\,x}}{RI_{30-39}^{U}\big|^{date\,x}} \quad \text{(2 of the manuscript)}$$

The previous mathematical demonstration validates the relationship among the B_{60+} profile (set of $B_{60+}^{date\,x}$ values), the E_{60+} profile (set of E_{60+}^{t} values, being t the time from vaccination) and the one-day vaccination profile (f_1 value) proposed in the manuscript.

DEMONSTRATION 2

Let us now consider that in the $date\ x$, the 60+ group is constituted for $P_{60+}^{V_1}\big|^{date\ x}$ individuals that were Vaccinated on the $date\ j$, $P_{60+}^{V_2}\big|^{date\ x}$ individuals that were Vaccinated on the next day to the $date\ j$, that is, the $date\ j\ +\ 1$ and $P_{60+}^{U}|^{date\ x}$ Unvaccinated individuals. Note that, since in this system the only vaccination dates were the $date\ j$ and the $date\ j+1$, the following equalities are valid: a) $P_{60+}^{V_1}\big|^{date\ j+1} = P_{60+}^{V_1}\big|^{date\ x}$, b) $P_{60+}^{V_2}\big|^{date\ j+2} = P_{60+}^{V_2}\big|^{date\ x}$, and c) $P_{60+}^{U}|^{date\ j+2} = P_{60+}^{U}|^{date\ x}$.

As in demonstration 1, it is now desirable to valid Eq. 4 of the manuscript, that is, the following equation:

$$B_{60+}^{date\ x} = \left({f_1}/{100}\right)(E_{60+}^{m}) + \left({f_2}/{100}\right)(E_{60+}^{m-1}) \tag{SM3-6}$$

For this system,

$$f_1 = \left(\frac{P_{60+}^{V_1}\big|^{date\ j+1}}{P_{60+}^{V_1}\big|^{date\ j+1} + P_{60+}^{V_2}\big|^{date\ j+2} + P_{60+}^{U}\big|^{date\ j+2}}\right)(100) = \left(\frac{P_{60+}^{V_1}\big|^{date\ x}}{P_{60+}^{V_1}\big|^{date\ x} + P_{60+}^{V_2}\big|^{date\ x} + P_{60+}^{U}\big|^{date\ x}}\right)(100), \text{ and}$$

$$f_2 = \left(\frac{P_{60+}^{V_2}\big|^{date\ j+2}}{P_{60+}^{V_1}\big|^{date\ j+1} + P_{60+}^{V_2}\big|^{date\ j+2} + P_{60+}^{U}|^{date\ j+2}}\right)(100) = \left(\frac{P_{60+}^{V_2}\big|^{date\ x}}{P_{60+}^{V_1}\big|^{date\ x} + P_{60+}^{V_2}\big|^{date\ x} + P_{60+}^{U}|^{date\ x}}\right)(100)$$

$$E_{60+}^{m} = 1 - \frac{\left(\frac{CC_{60+}^{V_1}\big|^{date\ x}}{P_{60+}^{V_1}\big|^{date\ x}}\right)}{\left(\frac{CC_{60+}^{U}\big|^{date\ x}}{P_{60+}^{U}\big|^{date\ x}}\right)}, \text{ and } E_{60+}^{m-1} = 1 - \frac{\left(\frac{CC_{60+}^{V_2}\big|^{date\ x}}{P_{60+}^{V_2}\big|^{date\ x}}\right)}{\left(\frac{CC_{60+}^{U}\big|^{date\ x}}{P_{60+}^{U}\big|^{date\ x}}\right)}.$$

In the last expressions, $CC_{60+}^{V_1}\big|^{date\ x}$ denotes the number of confirmed cases that presented onset symptoms specifically on $date\ x$, counting only the cases that occurred in the subgroup V_1, which only contains those individuals that completed their respective vaccination schemes in the $date\ j$ (first vaccination day); note that the total number of members in the subgroup V_1 is $P_{60+}^{V_1}\big|^{date\ x}$. Similarly, $CC_{60+}^{V_2}\big|^{date\ x}$ denotes the number of confirmed cases that presented onset symptoms specifically on $date\ x$, but now counting only the cases in the subgroup V_2, which only contains those individuals that completed their respective vaccination schemes in the $date\ j+1$ (second vaccination day); the total number of members in the subgroup V_2 is $P_{60+}^{V_2}\big|^{date\ x}$. Finally, $CC_{60+}^{U}|^{date\ x}$ denotes the number of confirmed cases that presented onset symptoms specifically on $date\ x$ in the subgroup U, which only contains those individuals of the group 60+ that remained unvaccinated in the $date\ x$; the number of members in the unvaccinated subgroup U is $P_{60+}^{U}|^{date\ x}$. Besides, note that, on the $date\ x$, the individuals vaccinated the $date\ j$ have m days of time from vaccination, but, on the same $date\ x$, the individuals vaccinated on the $date\ j+1$ have only $m-1$ days of time from vaccination.

Thus, the following expression can be obtained by substituting the last expressions in Eq. SM3-6.

$$B_{60+}^{date\ x} = \frac{P_{60+}^{V_1}\big|^{date\ x}}{P_{60+}^{V_1}\big|^{date\ x} + P_{60+}^{V_2}\big|^{date\ x} + P_{60+}^{U}\big|^{date\ x}}\left(1 - \frac{\left(\frac{CC_{60+}^{V_1}\big|^{date\ x}}{P_{60+}^{V_1}\big|^{date\ x}}\right)}{\left(\frac{CC_{60+}^{U}\big|^{date\ x}}{P_{60+}^{U}\big|^{date\ x}}\right)}\right) + \frac{P_{60+}^{V_2}\big|^{date\ x}}{P_{60+}^{V_1}\big|^{date\ x} + P_{60+}^{V_2}\big|^{date\ x} + P_{60+}^{U}\big|^{date\ x}}\left(1 - \frac{\left(\frac{CC_{60+}^{V_2}\big|^{date\ i}}{P_{60+}^{V_2}\big|^{date\ i}}\right)}{\left(\frac{CC_{60+}^{U}\big|^{date\ i}}{P_{60+}^{U}\big|^{date\ i}}\right)}\right) \tag{SM3-7}$$

However, after the multiplication of terms, the resulting expression is the following:

$$B_{60+}^{date\,x} = \frac{P_{60+}^{V_1}\Big|^{date\,x}}{P_{60+}^{V_1}\Big|^{date\,x} + P_{60+}^{V_2}\Big|^{date\,x} + P_{60+}^{U}\Big|^{date\,x}} - \frac{\left(\frac{CC_{60+}^{V_1}\Big|^{date\,x}}{P_{60+}^{V_1}\Big|^{date\,x} + P_{60+}^{V_2}\Big|^{date\,x} + P_{60+}^{U}\Big|^{date\,x}}\right)}{\left(\frac{CC_{60+}^{U}\big|^{date\,x}}{P_{60+}^{U}\big|^{date\,x}}\right)} +$$

$$\frac{P_{60+}^{V_2}\Big|^{date\,x}}{P_{60+}^{V_1}\Big|^{date\,x} + P_{60+}^{V_2}\Big|^{date\,x} + P_{60+}^{U}\Big|^{date\,x}} - \frac{\left(\frac{CC_{60+}^{V_2}\Big|^{date\,x}}{P_{60+}^{V_1}\Big|^{date\,x} + P_{60+}^{V_2}\Big|^{date\,x} + P_{60+}^{U}\Big|^{date\,x}}\right)}{\left(\frac{CC_{60+}^{U}\big|^{date\,x}}{P_{60+}^{U}\big|^{date\,x}}\right)}$$

Nevertheless, considering now the following equalities:

$$\frac{P_{60+}^{V1}\Big|^{date\,x}}{P_{60+}^{V1}\Big|^{date\,x} + P_{60+}^{V2}\Big|^{date\,x} + P_{60+}^{U}\Big|^{date\,x}} + \frac{P_{60+}^{V2}\Big|^{date\,x}}{P_{60+}^{V1}\Big|^{date\,x} + P_{60+}^{V2}\Big|^{date\,x} + P_{60+}^{U}\Big|^{date\,x}} = \frac{P_{60+}^{V1}\Big|^{date\,x} + P_{60+}^{V2}\Big|^{date\,x}}{P_{60+}^{V1}\Big|^{date\,x} + P_{60+}^{V2}\Big|^{date\,x} + P_{60+}^{U}\Big|^{date\,x}} =$$

$$\frac{P_{60+}^{V1}\Big|^{date\,x} + P_{60+}^{V2}\Big|^{date\,x} + P_{60+}^{U}\Big|^{date\,x} - P_{60+}^{U}\Big|^{date\,x}}{P_{60+}^{V1}\Big|^{date\,x} + P_{60+}^{V2}\Big|^{date\,x} + P_{60+}^{U}\Big|^{date\,x}} = 1 - \frac{P_{60+}^{U}\Big|^{date\,x}}{P_{60+}^{V1}\Big|^{date\,x} + P_{60+}^{V2}\Big|^{date\,x} + P_{60+}^{U}\Big|^{date\,x}}$$, as well as the following ones:

$$\frac{\left(\frac{CC_{60+}^{V_1}\Big|^{date\,x}}{P_{60+}^{V_1}\Big|^{date\,x} + P_{60+}^{V_2}\Big|^{date\,x} + P_{60+}^{U}\Big|^{date\,x}}\right)}{\left(\frac{CC_{60+}^{U}\big|^{date\,x}}{P_{60+}^{U}\big|^{date\,x}}\right)} + \frac{\left(\frac{CC_{60+}^{V_2}\Big|^{date\,x}}{P_{60+}^{V_1}\Big|^{date\,x} + P_{60+}^{V_2}\Big|^{date\,x} + P_{60+}^{U}\Big|^{date\,x}}\right)}{\left(\frac{CC_{60+}^{U}\big|^{date\,x}}{P_{60+}^{U}\big|^{date\,x}}\right)} = \frac{\left(\frac{CC_{60+}^{V_1}\Big|^{date\,x} + CC_{60+}^{V_2}\Big|^{date\,x}}{P_{60+}^{V_1}\Big|^{date\,x} + P_{60+}^{V_2}\Big|^{date\,x} + P_{60+}^{U}\Big|^{date\,x}}\right)}{\left(\frac{CC_{60+}^{U}\big|^{date\,x}}{P_{60+}^{U}\big|^{date\,x}}\right)}$$

$$= \frac{\left(\frac{CC_{60+}^{V_1}\Big|^{date\,x} + CC_{60+}^{V_2}\Big|^{date\,x}}{CC_{60+}^{U}\big|^{date\,x}}\right)}{\left(\frac{P_{60+}^{V_1}\Big|^{date\,x} + P_{60+}^{V_2}\Big|^{date\,x} + P_{60+}^{U}\big|^{date\,x}}{P_{60+}^{U}\big|^{date\,x}}\right)} = \left(\frac{CC_{60+}^{V_1}\Big|^{date\,x} + CC_{60+}^{V_2}\Big|^{date\,x}}{CC_{60+}^{U}\big|^{date\,x}}\right)\left(\frac{P_{60+}^{U}\big|^{date\,x}}{P_{60+}^{V_1}\Big|^{date\,x} + P_{60+}^{V_2}\Big|^{date\,x} + P_{60+}^{U}\big|^{date\,x}}\right)$$

It is possible to establish the following equalities:

$$B_{60+}^{date\,x} = 1 - \frac{P_{60+}^{U}\big|^{date\,x}}{P_{60+}^{V_1}\Big|^{date\,x} + P_{60+}^{V_2}\Big|^{date\,x} + P_{60+}^{U}\big|^{date\,x}} - \left(\frac{CC_{60+}^{V_1}\Big|^{date\,x} + CC_{60+}^{V_2}\Big|^{date\,x}}{CC_{60+}^{U}\big|^{date\,x}}\right)\left(\frac{P_{60+}^{U}\big|^{date\,x}}{P_{60+}^{V_1}\Big|^{date\,x} + P_{60+}^{V_2}\Big|^{date\,x} + P_{60+}^{U}\big|^{date\,x}}\right)$$

$$= 1 - \left(1 + \left(\frac{CC_{60+}^{V_1}\Big|^{date\,x} + CC_{60+}^{V_2}\Big|^{date\,x}}{CC_{60+}^{U}\big|^{date\,x}}\right)\right)\left(\frac{P_{60+}^{U}\big|^{date\,x}}{P_{60+}^{V_1}\Big|^{date\,x} + P_{60+}^{V_2}\Big|^{date\,x} + P_{60+}^{U}\big|^{date\,x}}\right)$$

Alternatively, by reordering the previous equation, the following expression can be obtained:

$$B_{60+}^{date\,x} = 1 - \left(\frac{CC_{60+}^{U}\Big|^{date\,x} + CC_{60+}^{V_1}\Big|^{date\,x} + CC_{60+}^{V_2}\Big|^{date\,x}}{CC_{60+}^{U}\big|^{date\,x}}\right)\left(\frac{P_{60+}^{U}\big|^{date\,x}}{P_{60+}^{V_1}\Big|^{date\,x} + P_{60+}^{V_2}\Big|^{date\,x} + P_{60+}^{U}\big|^{date\,x}}\right) =$$

$$= 1 - \frac{\left(\frac{CC_{60+}^{U}\Big|^{date\,x} + CC_{60+}^{V1}\Big|^{date\,x} + CC_{60+}^{V2}\Big|^{date\,x}}{CC_{60+}^{U}\big|^{date\,x}}\right)}{\left(\frac{P_{60+}^{V1}\Big|^{date\,x} + P_{60+}^{V2}\Big|^{date\,x} + P_{60+}^{U}\Big|^{date\,x}}{P_{60+}^{U}\big|^{date\,x}}\right)} = 1 - \frac{\left(\frac{CC_{60+}^{U}\Big|^{date\,x} + CC_{60+}^{V1}\Big|^{date\,x} + CC_{60+}^{V2}\Big|^{date\,x}}{P_{60+}^{V1}\Big|^{date\,x} + P_{60+}^{V2}\Big|^{date\,x} + P_{60+}^{U}\Big|^{date\,x}}\right)}{\left(\frac{CC_{60+}^{U}\big|^{date\,x}}{P_{60+}^{U}\big|^{date\,x}}\right)} \qquad \text{(SM3-8)}$$

Identifying now that $\left(\frac{CC_{60+}^{U}\Big|^{date\,x} + CC_{60+}^{V1}\Big|^{date\,x} + CC_{60+}^{V2}\Big|^{date\,x}}{P_{60+}^{V1}\Big|^{date\,x} + P_{60+}^{V2}\Big|^{date\,x} + P_{60+}^{U}\Big|^{date\,x}}\right) = \frac{RI_{60+}^{T}\big|^{date\,x}}{100000}$, and $\left(\frac{CC_{60+}^{U}\big|^{date\,x}}{P_{60+}^{U}\big|^{date\,x}}\right) = \frac{RI_{60+}^{U}\big|^{date\,x}}{100000}$

It can finally be obtained following equation:

$$B_{60+}^{date\ x} = 1 - \frac{RI_{60+}^{T}\big|^{date\ x}}{RI_{60+}^{U}\big|^{date\ x}} \quad \text{(SM3-9)}$$

Note that Eqs. SM3-9 and SM3-4 are identical. Thus, despite the number of vaccination days (one day -demonstration 1-, two days -demonstration 2- or any other amount of days), the $B_{60+}^{date\ x}$ values can be calculated by using Eq. 2 of the manuscript, which, as mentioned above, implicitly contains the equations SM3-4 and SM3-5.

Besides, just like Eq. SM3-1 was expanded to produce Eq. SM3-6, this equation (Eq. SM3-6) can be expanded to consider a 60+ group constituted by several subgroups vaccinated, each of them on consecutive days, as indicated in the equation presented below (Eq. 5 of the manuscript).

$$B_{60+}^{date\ x} = \left({f_1}/{100}\right)(E_{60+}^{m}) + \left({f_2}/{100}\right)(E_{60+}^{m-1}) + \left({f_3}/{100}\right)(E_{60+}^{m-2}) + \cdots + \left({f_{77}}/{100}\right)(E_{60+}^{m-76}) + \left({f_{78}}/{100}\right)(E_{60+}^{m-77}) \quad \text{(5 of the manuscript)}$$

The last equation is a fundamental mathematical relationship for the proposed data processing strategy.
