# Supplementary material for: A Novel Approach to Obtain Vaccine Effectiveness Continuous Profiles. Example Case: COVID-19 in Elderly Mexicans
Source: Vaccines (Basel). 2023 Mar 23;11(4):719. doi: 10.3390/vaccines11040719 (PMC10142991; doi:10.3390/vaccines11040719)
Supplement: Supplementary file 1 [file vaccines-11-00719-s001.zip › Supplementary Material 4.pdf]

As mentioned in the manuscript, the system of equations that establish the complex relationship among the B_{60+} profile, the E_{60+} profile and the vaccination profile is represented by the following mathematical expression:

$$B_{60+}^{date\ x} = \left({f_1}/{100}\right)(E_{60+}^{m}) + \left({f_2}/{100}\right)(E_{60+}^{m-1}) + \left({f_3}/{100}\right)(E_{60+}^{m-2}) + \cdots + \left({f_{77}}/{100}\right)(E_{60+}^{m-76}) + \left({f_{78}}/{100}\right)(E_{60+}^{m-77}) \quad \text{(SM4-1)}$$

In the last expression, it is evident that the different E_{60+}^{t} values can not be calculated directly (t denotes the time from vaccination). Therefore, they must be estimated through the systematic fitting process that will be described later.

However, at this moment, it is convenient to mention that the physical meaning of the E_{60+}^{t} values allow assuming that such a set of values must be part of a curve with a smooth shape. Thus, since its high flexibility, the Eq. SM4.2 is proposed to represent numerically the E_{60+} profile (set of E_{60+}^{t} values).

$$E_{60+}^{t} = \left((A_L)\left(\frac{\left(e^{(t+D_L)(C_L)}\right) - \left(e^{(t+D_L)(C_L)}\right)}{\left(e^{(t+D_L)(C_L)}\right) + \left(e^{(t+D_L)(C_L)}\right)}\right) + B_L \right)\left((A_R)\left(\frac{\left(e^{(t+D_R)(C_R)}\right) - \left(e^{(t+D_R)(C_R)}\right)}{\left(e^{(t+D_R)(C_R)}\right) + \left(e^{(t+D_R)(C_R)}\right)}\right) + B_R \right) \quad \text{(SM4-2)}$$

In such a procedure, the A_L, B_L, C_L, D_L, A_R, B_R, C_R, and D_R parameters (highlighted in blue in Eq. SM4-2) will be systematically modified, iteration by iteration, to improve the data's fitting (section SM4.1). However, in each iteration, a value will be assigned to each parameter, allowing the obtainment of a mathematical expression where each E_{60+}^{t} value depends only on the specific value of t (time from vaccination; parameter highlighted in red in Eq. SM4-2). As the only exception, assuming that the daily E_{60+}^{t} values are always positive, when for a given t value Eq. SM4-1 produces negative values, then $E_{60+}^{t} = 0$. Besides, since the t values have been referenced to the day on which each individual completed his vaccination scheme, and it is expected that the effect of the vaccines begins shortly after applying the first dose, in vaccination schemes with two doses, the t value may become negative.

SM4.1. The numerical fitting process to obtain the vaccine effectiveness profile (E_{60+} profile).

The required numerical fitting process can be implemented by sequentially performing the following actions:

1) Assign the initial values for each of the eight parameters highlighted in blue in Eq. SM4-2; it is recommended to use the reference values presented in Table SM4.1.

2) Substitute in Eq. SM4-2 the values of the parameters highlighted in blue considered in each iteration and the respective t values to calculate the set of daily E_{60+}^{t} values for the required t interval.
3) Evaluate each term in Eq. SM4-1 (n terms, being n the number of vaccination days) for each of the q days (days for which a $B_{60+}^{date\ x}$ value is available) within the defined estimation period; the profile obtained will be called $B_{60+,iterative}$ profile.
4) Calculate the normalized square deviation between each real-world value of $1 - B_{60+}^{date\ x}$ and each iterative value of $1 - B_{60+,iterative}^{date\ x}$ as follows: $\left(\frac{(1-B_{60+}^{date\ x})-(1-B_{60+,iterative}^{date\ x})}{1-B_{60+}^{date\ x}}\right)^2$. Note that q different values must be computed, one for each available $B_{60+}^{date\ x}$ value.
5) Evaluate, from the q values generated in the previous paragraph, the root of the normalized mean square deviation of the iterative values concerning the real-world data ($RNMS$ value), as follows: $RNMS = \sqrt{\frac{\sum_{i=1}^{q}\left(\frac{(1-B_{60+}^{date\ x})-(1-B_{60+,iterative}^{date\ x})}{1-BE_{60+}^{date\ x}}\right)^2}{q}}$.
6) Systematically modify the parameters of the proposed equation (Eq. SM4-2) to assign, within the recommended search intervals (Table SM4.1), a new set of parameters highlighted in blue that improves the fitting by decreasing the $RNMS$ value. Multiple algorithms allow varying these parameters systematically. However, in this study, the algorithm known as Generalized Reduced Gradient (GRG) Nonlinear was used [SM4-1].
7) Repeat steps 2 to 6 until the minimum $RNMS$ value is obtained. The final result of this iterative procedure will be named B_{fitted} profile.

Although the above-described procedure can be carried out in multiple ways, it is recommended to use the add-in known as Solver of the Excel software of Microsoft [50-53]. A preconfigured worksheet for doing this activity (Supplementary Material # 5), a user guide describing how to use this tool to estimate the E_{60+} profile for a hypothetic example case (Supplementary Material # 6) and a worksheet with a hypothetical example of such estimation (Supplementary Material #7) are included as Supplementary Materials.

References

50. Microsoft. Define and solve a problem by using Solver. Available on: https://support.microsoft.com/en-us/office/define-and-solve-a-problem-by-using-solver-5d1a388f-079d-43ac-a7eb-f63e45925040 (accessed on 01 February 2023).
51. Yu, D.C.; Fagan, J.E.; Foote, B.; Aly, A.A. An optimal load flow study by the generalized reduced gradient approach. Electric Power Systems Research 1986, 10(1), 47–53. doi:10.1016/0378-7796(86)90048-9

52. Lasdon, L.S.; Fox, R.L.; Ratner, M.W. Nonlinear optimization using the generalized reduced gradient method. Revue française d'automatique, informatique, recherche opérationnelle. Recherche opérationnelle 1974, 3, 73-103. Available online at: http://www.numdam.org/item/?id=RO_1974__8_3_73_0 (accessed on 14 March 2023)
53. Facó, J.L.D. A generalized reduced gradient algorithm for solving large-scale discrete-time nonlinear optimal control problems. IFAC Proceedings 1989, 22(2), 45–50. doi:10.1016/b978-0-08-037869-5.50011-x

Table SM4.1. Reference values and search intervals for the indicated parameters.

Parameter	Reference values	Search interval
A_L<br>B_L<br>C_L<br>D_L	3.0<br>2.5<br>0.02<br>-70	2.5 to 5.0<br>-2.0 to 4.0<br>-5.00 to 5.00<br>-100 to -40
A_R<br>B_R<br>C_R<br>D_R	-5.0<br>5.0<br>0.015<br>50	-10.0 to -4.5<br>-5.0 to 5.4<br>0.010 to 5.000<br>0 to 100
