# Supplementary material for: A Novel Approach to Obtain Vaccine Effectiveness Continuous Profiles. Example Case: COVID-19 in Elderly Mexicans
Source: Vaccines (Basel). 2023 Mar 23;11(4):719. doi: 10.3390/vaccines11040719 (PMC10142991; doi:10.3390/vaccines11040719)
Supplement: Supplementary file 1 [file vaccines-11-00719-s001.zip › Supplementary Material 6.pdf]

USER GUIDE (Effectiveness Worksheet)

The effectiveness profile representing the global behavior of the set of vaccines applied to the 60+ group in Mexico was obtained by using an Excel worksheet (Excel file named: Supplementary Material #5).

Since this file can easily be adapted to many other populational groups (G), this USER GUIDE aims to help USERS utilize such a computational tool to obtain the effectiveness profile characterizing their studied group. For it, it is required that ***USERS*** have the respective B_G and vaccination profiles for their target group (G). Only for illustrative purposes, to exemplify the use of this computational tool, here are defined hypothetical but realistic profiles (they are not real data) for the B_G profile and for the vaccination profile; both are included in Supplementary material # 7 in the worksheet: Hypothetical Profiles.

SECTION 1. INFORMATION PREPROCESSING

The objective of the actual section is to modify the required information to a format that allows its insertion in the main worksheet (Excel file named Supplementary Material #5). Note that, for illustrative purposes, such an Excel worksheet has been maintained with the final results of the real system presented in the paper.

STEP 1:

Adapt the **USERS**' f_j profile (vaccination profile used in the study group) to a table with two columns; in the left column, write the dates, while in the right one, the fractions of the total group that completed their respective vaccination schemes each day (f_j values). It is obligatory to use consecutive dates, as skipping dates is not valid for this worksheet. Figure S1 shows the initial data corresponding to the hypothetical profile proposed and its histogram for the vaccination profile (available in Supplementary Material # 7; worksheet named: Hypothetical profiles. Columns A and B).

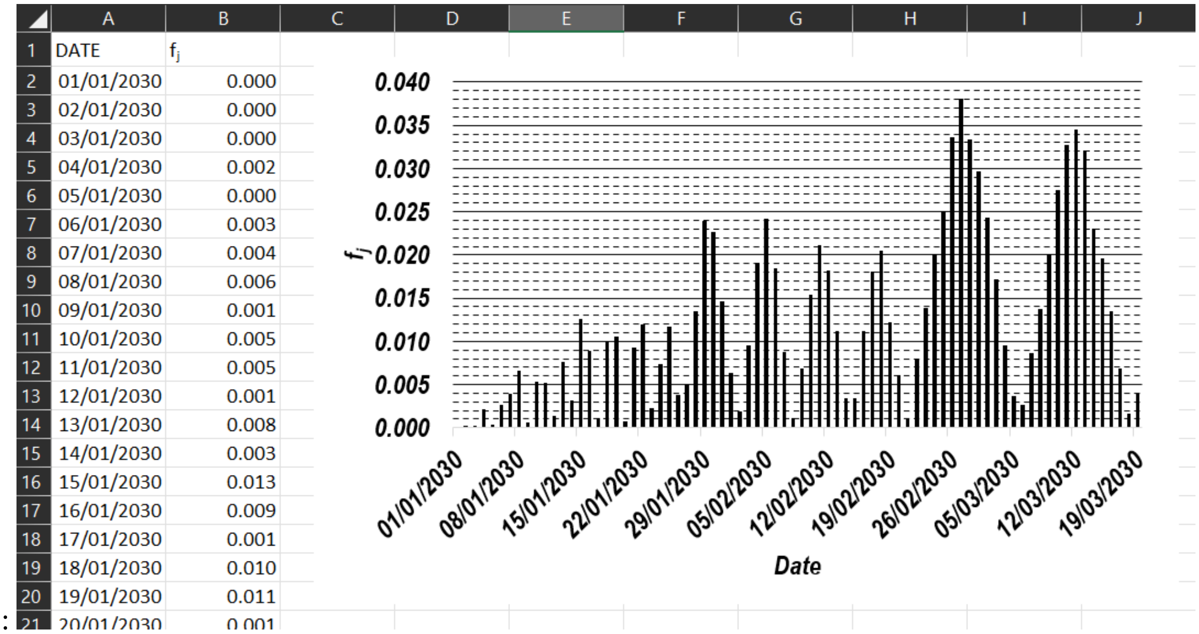

	A	B
1	DATE	f_j
2	01/01/2030	0.000
3	02/01/2030	0.000
4	03/01/2030	0.000
5	04/01/2030	0.002
6	05/01/2030	0.000
7	06/01/2030	0.003
8	07/01/2030	0.004
9	08/01/2030	0.006
10	09/01/2030	0.001
11	10/01/2030	0.005
12	11/01/2030	0.005
13	12/01/2030	0.001
14	13/01/2030	0.008
15	14/01/2030	0.003
16	15/01/2030	0.013
17	16/01/2030	0.009
18	17/01/2030	0.001
19	18/01/2030	0.010
20	19/01/2030	0.011
21	20/01/2030	0.001

Figure S1. Initial data of the f_j profile and the whole histogram showing the hypothetical vaccination profile (f_j) used to exemplify.

STEP 2:

Adapt the available B_G profile (Beneficial Effect characterizing the studied group (G)) to a table with two columns; in the left column, write the dates, while in the right one, the daily values of B_G, as is shown in Figure S2. Note that it is recommendable that the $BE_{S,G}$ profile is as large as possible to improve the fitting level obtained with the proposed methodology; the application

of the methodology using a $BE_{S,G}$ profile constituted by comparatively few data, usually will provide incorrect or, at least, imprecise results. In Figure S2 are shown the initial data of the proposed B_G profile, as well as the plot showing the whole B_G profile (available in Supplementary Material # 7, worksheet named: Hypothetical profiles. Columns A and C).

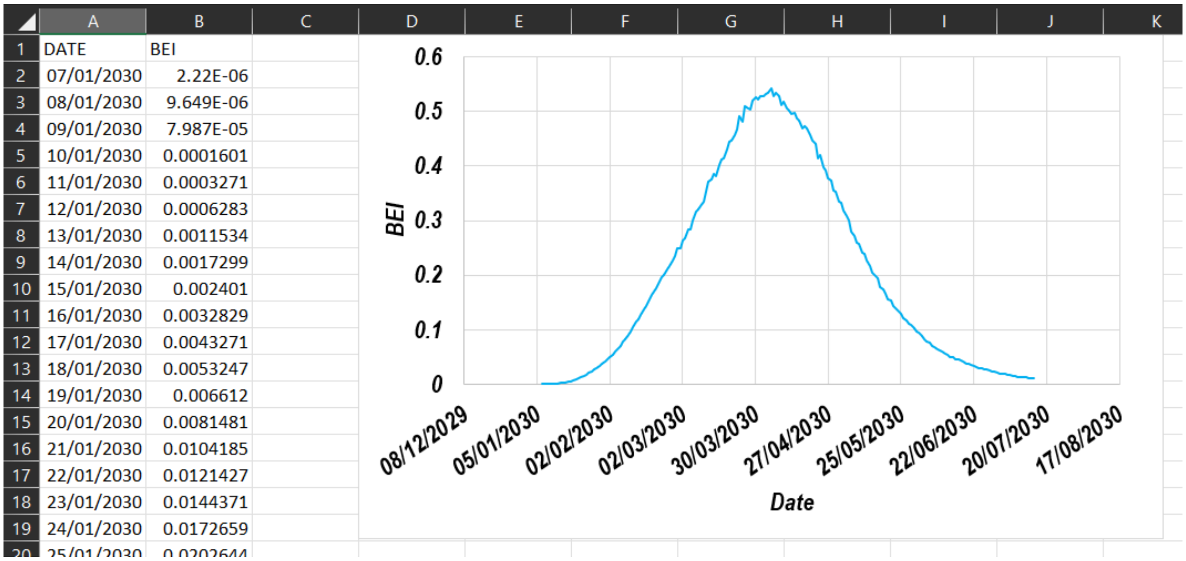

	A	B
1	DATE	BEI
2	07/01/2030	2.22E-06
3	08/01/2030	9.649E-06
4	09/01/2030	7.987E-05
5	10/01/2030	0.0001601
6	11/01/2030	0.0003271
7	12/01/2030	0.0006283
8	13/01/2030	0.0011534
9	14/01/2030	0.0017299
10	15/01/2030	0.002401
11	16/01/2030	0.0032829
12	17/01/2030	0.0043271
13	18/01/2030	0.0053247
14	19/01/2030	0.006612
15	20/01/2030	0.0081481
16	21/01/2030	0.0104185
17	22/01/2030	0.0121427
18	23/01/2030	0.0144371
19	24/01/2030	0.0172659
20	25/01/2030	0.0202644

Figure S2. Initial data of the B_G profile and plot showing the whole B_G profile.

SECTION 2: OBTAINING THE LIMITS OF AN INTERVAL

STEP 1:

Define the first day at which it could be expectable that vaccine(s) would have produced a noticeable beneficial effect; consider the day at which the respective vaccination schemes are completed as the reference point (day zero). In single-dose vaccines, it is recommendable to use zero days. In the case of multi-dose vaccines, it is recommendable to use a negative value that includes, in excess, the day on which the first doses were administrated. For example, if the second doses were administered on average 30 days after the first ones, define the value as -37 days or lower (e.g., -38, -39, and so on).

Note that if the value is defined as moderately lower, the model would usually identify the correct value. However, if the selected value is higher than the real value, the model tends to be as imprecise as the difference between the defined value and the actual one. The proposed methodology can indistinctly be used for single-dose or multi-dose systems. Therefore, to show the versatility of our proposal, since the system considered in the paper was a two-doses system, a hypothetical single-dose system was selected for the example described here. Therefore, the above-mentioned initial value was defined as zero days.

STEP 2:

Define the first day at which it could be expectable that the global effectiveness of the vaccine(s) would remain practically constant over time, inspecting the available daily B_G values; take the same reference day as in **STEP 1**. The ideal circumstance is that enough daily B_G values will be available to cover, as possible, the whole interval of effectiveness. For the hypothetical example, since there are available 190 values for the B_G profile, it is convenient to use 190 days as the superior limit of the interval.

SECTION 3: SUBSTITUTING THE REQUIRED INFORMATION IN THE MAIN WORKSHEET

STEP 1:

Considering the interval 0 to 190 formed with the previously defined limits inferior and superior. The values in column I of the main worksheet must be substituted as follows. Insert in cell $I2$ the maximum value of the defined interval (that is, 190), continue inserting in cell $I3$ the value 189, and continue consecutively down, up to insert the minimum value of the interval, that is, inserting the value zero in the cell $I192$. Later, erase the rest of the preloaded data of column I by cleaning the data of the cells $I193$, $I194$, etc., as shown in Figure S3. In addition, erase the rest of the preloaded data of columns J, K, and L by cleaning the data of cells J193, J194,… K193, K194, … L193, L194, …, as shown in Figure S3

	I	J	K	L
1	Days referred to second dose administration	Left factor	Right factor	$E_{S,60+}$
2	190	5.1737	0.0188	0.0975
3	189	5.1700	0.0193	0.0997
4	188	5.1662	0.0197	0.1019
5	187	5.1623	0.0202	0.1042
6	186	5.1583	0.0207	0.1065
7	185	5.1541	0.0211	0.1089
8	184	5.1499	0.0216	0.1114
9	183	5.1455	0.0221	0.1139
10	182	5.1409	0.0227	0.1165
11	181	5.1362	0.0232	0.1191
12	180	5.1314	0.0237	0.1218
13	179	5.1264	0.0243	0.1246
14	178	5.1213	0.0249	0.1274
15	177	5.1160	0.0255	0.1303
16	176	5.1105	0.0261	0.1333
17	175	5.1049	0.0267	0.1363
18	174	5.0991	0.0273	0.1395

…

175	17	0.6343	1.2880	0.8170
176	16	0.6122	1.3170	0.8062
177	15	0.5905	1.3465	0.7951
178	14	0.5693	1.3766	0.7837
179	13	0.5487	1.4072	0.7721
180	12	0.5285	1.4384	0.7601
181	11	0.5087	1.4701	0.7479
182	10	0.4895	1.5024	0.7355
183	9	0.4707	1.5353	0.7227
184	8	0.4524	1.5687	0.7097
185	7	0.4345	1.6027	0.6964
186	6	0.4171	1.6373	0.6829
187	5	0.4001	1.6725	0.6692
188	4	0.3835	1.7082	0.6552
189	3	0.3674	1.7445	0.6409
190	2	0.3517	1.7814	0.6265
191	1	0.3363	1.8189	0.6118
192	0	0.3214	1.8570	0.5968
193				
194				
195				

Figure S3. Sections initial (left image) and final (right image) of the columns I, J, K, and L, after modification described in STEP 1 of SECTION 3.

STEP 2:

Mark, with yellow color, the row that contains a zero value in column I; for this example, row 192 (as shown in Figure S4).

	A	B	C	D	E	F	G	H	I	J	K	L	M
181	07/02/2021								11	0.5087	1.4701	0.7479	
182	08/02/2021								10	0.4895	1.5024	0.7355	
183	09/02/2021								9	0.4707	1.5353	0.7227	
184	10/02/2021								8	0.4524	1.5687	0.7097	
185	11/02/2021								7	0.4345	1.6027	0.6964	
186	12/02/2021								6	0.4171	1.6373	0.6829	
187	13/02/2021								5	0.4001	1.6725	0.6692	
188	14/02/2021								4	0.3835	1.7082	0.6552	
189	15/02/2021								3	0.3674	1.7445	0.6409	
190	16/02/2021								2	0.3517	1.7814	0.6265	
191	17/02/2021								1	0.3363	1.8189	0.6118	
192	18/02/2021								0	0.3214	1.8570	0.5968	
193	19/02/2021												

Figure S4. Row 192, which was marked in yellow, as it includes the zero value in column I.

Later, the **USER** must copy (**Ctrl + C**) all the available $BE_{S,G}$ data and paste (**Ctrl + V**) them in column C of the main worksheet (column titled $BE_{S,60+}$), starting at the yellow row (for this example, row 192), as shown in Figure S5. Note: In case additional data exist in column C, eliminate all data that does not correspond to the interest profile.

	A	B	C	D	E	F	G	H	I	J	K	L
184	10/02/2021								8	0.4524	1.5687	0.7097
185	11/02/2021								7	0.4345	1.6027	0.6964
186	12/02/2021								6	0.4171	1.6373	0.6829
187	13/02/2021								5	0.4001	1.6725	0.6692
188	14/02/2021								4	0.3835	1.7082	0.6552
189	15/02/2021								3	0.3674	1.7445	0.6409
190	16/02/2021								2	0.3517	1.7814	0.6265
191	17/02/2021								1	0.3363	1.8189	0.6118
192	18/02/2021		2.2198E-06						0	0.3214	1.8570	0.5968
193	19/02/2021		9.6492E-06									
194	20/02/2021		7.987E-05									
195	21/02/2021		0.00016005									
196	22/02/2021		0.00032708									
197	23/02/2021		0.00062834									
198	24/02/2021		0.00115344									
199	25/02/2021		0.00172985									
200	26/02/2021		0.002401									
201	27/02/2021		0.00328293									
202	28/02/2021		0.00432712									
203	01/03/2021		0.00532471									

Figure S5. ***$BE_{S,G}$ data pasted at the right position.***

STEP 3:

Modify the original dates in column A of the main worksheet (see left side in Figure 6; “BEFORE”) to correspond with those of the considered $BE_{S,G}$ profile (see the right side in Figure 6; “AFTER”). Start writing, in cell $A192$, the date of the first available $BE_{S,G}$ value (for this example: 07/01/2030) continuing up, day to day, up to cell $A2$ (for this example, must be written in such a cell: 01/07/2029). Later, continue in the down direction with the same logic until cell $A381$ (the position of the last available value of the $BE_{S,G}$ profile; 15/07/2030), and eliminate all remaining dates in this column. After these modifications, the worksheet will look like as shown on the right side of Figure S6.

	A	B	C
183	09/02/2021		
184	10/02/2021		
185	11/02/2021		
186	12/02/2021		
187	13/02/2021		
188	14/02/2021		
189	15/02/2021		
190	16/02/2021		
191	17/02/2021		
192	18/02/2021		2.2198E-06
193	19/02/2021		9.6492E-06
194	20/02/2021		7.987E-05
195	21/02/2021		0.00016005
196	22/02/2021		0.00032708
197	23/02/2021		0.00062834
198	24/02/2021		0.00115344
199	25/02/2021		0.00172985
200	26/02/2021		0.002401
201	27/02/2021		0.00328293
202	28/02/2021		0.00432712
203	01/03/2021		0.00532471
204	02/03/2021		0.00661202
205	03/03/2021		0.0081481
206	04/03/2021		0.01041849
207	05/03/2021		0.01214266

BEFORE

	A	B	C
183	29/12/2029		
184	30/12/2029		
185	31/12/2029		
186	01/01/2030		
187	02/01/2030		
188	03/01/2030		
189	04/01/2030		
190	05/01/2030		
191	06/01/2030		
192	07/01/2030		2.2198E-06
193	08/01/2030		9.6492E-06
194	09/01/2030		7.987E-05
195	10/01/2030		0.00016005
196	11/01/2030		0.00032708
197	12/01/2030		0.00062834
198	13/01/2030		0.00115344
199	14/01/2030		0.00172985
200	15/01/2030		0.002401
201	16/01/2030		0.00328293
202	17/01/2030		0.00432712
203	18/01/2030		0.00532471
204	19/01/2030		0.00661202
205	20/01/2030		0.0081481
206	21/01/2030		0.01041849
207	22/01/2030		0.01214266

AFTER

Figure S6. Modification of column A (column for dates). Left side: Worksheet before modification. Right side: Worksheet after modification.

STEP 4:

Modify the original values in column B of the main worksheet (see left side in Figure S7; “BEFORE”) to correspond with those of the **USER’s** vaccination profile (see the right side in Figure S7; “AFTER”). For it, copy (**Ctrl + C**) all the values of the **USER’s** vaccination profile and paste (**Ctrl + V**) them, starting in the cell of column B of the main worksheet that allows the dates and the f_j values are in agreement, as in the original version. For this example, since the first vaccination day was January 1, 2030, after this modification, the worksheet will look like shown on the right side of Figure S7; the new data will be located from cell $B186$ (01/01/2030) up to cell $B263$ (19/03/2030). Later, eliminate all the unnecessary values in column B.

	A	B	C
183	29/12/2029		
184	30/12/2029		
185	31/12/2029		
186	01/01/2030		
187	02/01/2030		
188	03/01/2030		
189	04/01/2030		
190	05/01/2030		
191	06/01/2030		
192	07/01/2030		2.2198E-06
193	08/01/2030		9.6492E-06
194	09/01/2030		7.987E-05
195	10/01/2030		0.00016005
196	11/01/2030		0.00032708
197	12/01/2030		0.00062834
198	13/01/2030		0.00115344
199	14/01/2030		0.00172985
200	15/01/2030		0.002401
201	16/01/2030		0.00328293
202	17/01/2030		0.00432712
203	18/01/2030		0.00532471
204	19/01/2030		0.00661202
205	20/01/2030		0.0081481
206	21/01/2030		0.01041849
207	22/01/2030		0.01214266

BEFORE

	A	B	C
183	29/12/2029		
184	30/12/2029		
185	31/12/2029		
186	01/01/2030	0.000	
187	02/01/2030	0.000	
188	03/01/2030	0.000	
189	04/01/2030	0.002	
190	05/01/2030	0.000	
191	06/01/2030	0.003	
192	07/01/2030	0.004	2.2198E-06
193	08/01/2030	0.006	9.6492E-06
194	09/01/2030	0.001	7.987E-05
195	10/01/2030	0.005	0.00016005
196	11/01/2030	0.005	0.00032708
197	12/01/2030	0.001	0.00062834
198	13/01/2030	0.008	0.00115344
199	14/01/2030	0.003	0.00172985
200	15/01/2030	0.013	0.002401
201	16/01/2030	0.009	0.00328293
202	17/01/2030	0.001	0.00432712
203	18/01/2030	0.010	0.00532471
204	19/01/2030	0.011	0.00661202
205	20/01/2030	0.001	0.0081481
206	21/01/2030	0.009	0.01041849
207	22/01/2030	0.012	0.01214266

AFTER

Figure S7. Substitution of the f_j values characterizing the considered vaccination profile.

SECTION 4: MODIFYING PRECODED FORMULAS TO ADAPT TO THE SPECIFIC CHARACTERISTICS OF INTRODUCED PROFILES

STEP 1:

Cut (**Ctrl + X**) the cell $D234$ (previously marked in yellow) and paste (**Ctrl + V**) it in the cell of column D marked in yellow ($D192$), as shown in Figure S8.

	A	B	C	D	E	F	G
225	09/02/2030	0.007	0.08924368				
226	10/02/2030	0.015	0.09639479				
227	11/02/2030	0.021	0.10398238				
228	12/02/2030	0.018	0.1154966				
229	13/02/2030	0.011	0.1179826				
230	14/02/2030	0.003	0.12589625				
231	15/02/2030	0.003	0.13659654				
232	16/02/2030	0.011	0.14365848				
233	17/02/2030	0.018	0.15162868				
234	18/02/2030	0.020	0.16295924	0.00555325	0.83704076	0.99444675	0.03536301
235	19/02/2030	0.012	0.16874562	0.00956432	0.83125438	0.99043568	0.03667045
236	20/02/2030	0.006	0.17522687	0.01011822	0.82477313	0.98988178	0.04007473
237	21/02/2030	0.001	0.18741163	0.01346428	0.81258837	0.98653572	0.04582416
238	22/02/2030	0.008	0.19695129	0.01681626	0.80304871	0.98318374	0.05031675

Cut (**Ctrl+X**) cell $D234$

	A	B	C	D	E	F	G	H	I	J	K	L
183	29/12/2029								9	0.4707	1.5353	0.7227
184	30/12/2029								8	0.4524	1.5687	0.7097
185	31/12/2029								7	0.4345	1.6027	0.6964
186	01/01/2030	0.000							6	0.4171	1.6373	0.6829
187	02/01/2030	0.000							5	0.4001	1.6725	0.6692
188	03/01/2030	0.000							4	0.3835	1.7082	0.6552
189	04/01/2030	0.002							3	0.3674	1.7445	0.6409
190	05/01/2030	0.000							2	0.3517	1.7814	0.6265
191	06/01/2030	0.003							1	0.3363	1.8189	0.6118
192	07/01/2030	0.004	2.2198E-06	0.00555325					0	0.3214	1.8570	0.5968
193	08/01/2030	0.006	9.6492E-06									
194	09/01/2030	0.001	7.987E-05									
195	10/01/2030	0.005	0.00016005									
196	11/01/2030	0.005	0.00032708									
[illegible]	[illegible]	[illegible]	[illegible]									

Paste (**Ctrl + V**) it in cell *D*192

Figure S8. Cut and paste process for the formula "SUMAPRODUCTO."

STEP 2:

Modify the interval considered in the recently pasted "SUMAPRODUCTO" formula (in cell D192 was previously written: "=SUMAPRODUCTO(B2:B283,$L$2:$L$283)") to adjust it to the effectiveness interval considered by the **USER** (after modification, for this case, in cell D192 must be written: "=SUMAPRODUCTO(B2:B192,$L$2:$L$192)"), as shown in Figure S9.

A	B	C	D	E	F	G	H	I	J	K	L
DATE	f_j	$BEI_{s,60+}$	BEI_{fitted}	$1\text{-}BEI_{s,60+}$	$1\text{-}BEI_{fitted}$	Normalized Squared Deviation		Days referred to second dose administration	Left factor	Right factor	$E_{s,60+}$
01/07/2029								190	5.1737	0.0188	0.0975
02/07/2029								189	5.1700	0.0193	0.0997
03/07/2029								188	5.1662	0.0197	0.1019
04/07/2029								187	5.1623	0.0202	0.1042
05/07/2029								186	5.1583	0.0207	0.1065
06/07/2029								185	5.1541	0.0211	0.1089
07/07/2029								184	5.1499	0.0216	0.1114
08/07/2029								183	5.1455	0.0221	0.1139
09/07/2029								182	5.1409	0.0227	0.1165
10/07/2029								181	5.1362	0.0232	0.1191
11/07/2029								180	5.1314	0.0237	0.1218
12/07/2029								179	5.1264	0.0243	0.1246
13/07/2029								178	5.1213	0.0249	0.1274
14/07/2029								177	5.1160	0.0255	0.1303
15/07/2029								176	5.1105	0.0261	0.1333
16/07/2029								175	5.1049	0.0267	0.1363
17/07/2029								174	5.0991	0.0273	0.1395
18/07/2029								173	5.0931	0.0280	0.1426
19/07/2029			SUMAPRODUCTO(matriz1, [matriz2], [matriz3], [matriz4], ...)					172	5.0870	0.0287	0.1459
20/07/2029								171	5.0806	0.0294	0.1492
21/07/2029								170	5.0741	0.0301	0.1527
22/07/2029								169	5.0673	0.0308	0.1562
23/07/2029								168	5.0604	0.0316	0.1597
24/07/2029								167	5.0532	0.0323	0.1634

BEFORE. Superior limit

	A	B	C	D	E	F	G	H	I	J	K	L
259	15/03/2030	0.020	0.38248582	0.15515809	0.61751418	0.84484191	0.13552237					
260	16/03/2030	0.013	0.40193147	0.15887161	0.59806853	0.84112839	0.1651675					
261	17/03/2030	0.007	0.41207051	0.16717212	0.58792949	0.83282788	0.17350875					
262	18/03/2030	0.002	0.41462574	0.18123128	0.58537426	0.81876872	0.15896951					
263	19/03/2030	0.004	0.43013324	0.19855391	0.56986676	0.80144609	0.16514025					
264	20/03/2030		0.44553803	0.21274385	0.55446197	0.78725615	0.17627907					
265	21/03/2030		0.44786543	SUMAPRODUCTO(matriz1, [matriz2], [matriz3], [matriz4], ...)								
266	22/03/2030		0.45634697	0.2255016	0.54365303	0.7744984	0.18030125					
267	23/03/2030		0.46654687	0.23294091	0.53345313	0.76705909	0.19176763					
268	24/03/2030		0.49265103	0.2455108	0.50734897	0.7544892	0.23728665					
269	25/03/2030		0.48150447	0.26168168	0.51849553	0.73831832	0.17974441					
270	26/03/2030		0.50919245	0.27631374	0.49080755	0.72368626	0.22513192					
271	27/03/2030		0.50610803	0.28694065	0.49389197	0.71305935	0.19691913					
272	28/03/2030		0.50418028	0.29298584	0.49581972	0.70701416	0.18143346					
273	29/03/2030		0.52056383	0.29898049	0.47943617	0.70101951	0.21360558					
274	30/03/2030		0.52614525	0.30953947	0.47385475	0.69046053	0.20895347					
275	31/03/2030		0.52288957	0.32429365	0.47711043	0.67570635	0.17326181					
276	01/04/2030		0.52863415	0.34064142	0.47136585	0.65935858	0.15906181					
277	02/04/2030		0.52821966	0.35217583	0.47178034	0.64782417	0.13923934					
278	03/04/2030		0.53305858	0.36014186	0.46694142	0.63985814	0.13713526					
279	04/04/2030		0.53505635	0.36506466	0.46494365	0.63493534	0.1336764					
280	05/04/2030		0.54207528	0.37400434	0.45792472	0.62599566	0.13470915					
281	06/04/2030		0.52854054	0.38644796	0.47145946	0.61355204	0.09083519					
282	07/04/2030		0.53442301	0.40263204	0.46557699	0.59736796	0.08012873					
283	08/04/2030		0.52741845	0.42200396	0.47258155	0.57799604	0.04975619					
284	09/04/2030		0.51120239	0.44667561	0.48879761	0.55332439	0.01742697					

BEFORE. Inferior limit

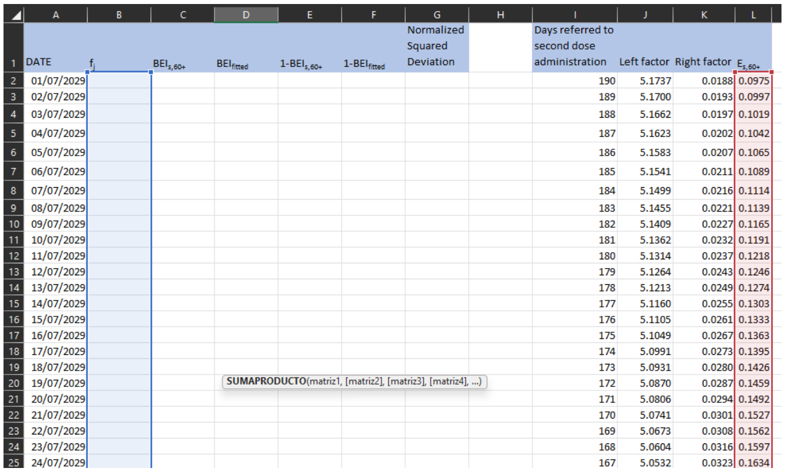

	A	B	C	D	E	F	G	H	I	J	K	L
1	DATE	f_j	$BEI_{s,60+}$	BEI_{fitted}	$1\text{-}BEI_{s,60+}$	$1\text{-}BEI_{fitted}$	Normalized Squared Deviation		Days referred to second dose administration	Left factor	Right factor	$E_{s,60+}$
2	01/07/2029								190	5.1737	0.0188	0.0975
3	02/07/2029								189	5.1700	0.0193	0.0997
4	03/07/2029								188	5.1662	0.0197	0.1019
5	04/07/2029								187	5.1623	0.0202	0.1042
6	05/07/2029								186	5.1583	0.0207	0.1065
7	06/07/2029								185	5.1541	0.0211	0.1089
8	07/07/2029								184	5.1499	0.0216	0.1114
9	08/07/2029								183	5.1455	0.0221	0.1139
10	09/07/2029								182	5.1409	0.0227	0.1165
11	10/07/2029								181	5.1362	0.0232	0.1191
12	11/07/2029								180	5.1314	0.0237	0.1218
13	12/07/2029								179	5.1264	0.0243	0.1246
14	13/07/2029								178	5.1213	0.0249	0.1274
15	14/07/2029								177	5.1160	0.0255	0.1303
16	15/07/2029								176	5.1105	0.0261	0.1333
17	16/07/2029								175	5.1049	0.0267	0.1363
18	17/07/2029								174	5.0991	0.0273	0.1395
19	18/07/2029								173	5.0931	0.0280	0.1426
20	19/07/2029			SUMAPRODUCTO(matriz1, [matriz2], [matriz3], [matriz4], ...)					172	5.0870	0.0287	0.1459
21	20/07/2029								171	5.0806	0.0294	0.1492
22	21/07/2029								170	5.0741	0.0301	0.1527
23	22/07/2029								169	5.0673	0.0308	0.1562
24	23/07/2029								168	5.0604	0.0316	0.1597
25	24/07/2029								167	5.0532	0.0323	0.1634

AFTER. Superior limit

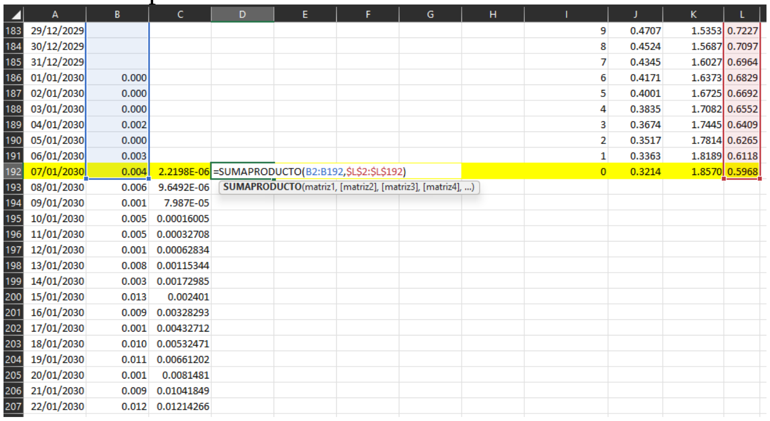

	A	B	C	D	E	F	G	H	I	J	K	L
183	29/12/2029								9	0.4707	1.5353	0.7227
184	30/12/2029								8	0.4524	1.5687	0.7097
185	31/12/2029								7	0.4345	1.6027	0.6964
186	01/01/2030	0.000							6	0.4171	1.6373	0.6829
187	02/01/2030	0.000							5	0.4001	1.6725	0.6692
188	03/01/2030	0.000							4	0.3835	1.7082	0.6552
189	04/01/2030	0.002							3	0.3674	1.7445	0.6409
190	05/01/2030	0.000							2	0.3517	1.7814	0.6265
191	06/01/2030	0.003							1	0.3363	1.8189	0.6118
192	07/01/2030	0.004	2.2198E-06	=SUMAPRODUCTO(B2:B192,$L$2:$L192)					0	0.3214	1.8570	0.5968
193	08/01/2030	0.006	9.6492E-06	SUMAPRODUCTO(matriz1, [matriz2], [matriz3], [matriz4], ...)								
194	09/01/2030	0.001	7.987E-05									
195	10/01/2030	0.005	0.00016005									
196	11/01/2030	0.005	0.00032708									
197	12/01/2030	0.001	0.00062834									
198	13/01/2030	0.008	0.00115344									
199	14/01/2030	0.003	0.00172985									
200	15/01/2030	0.013	0.002401									
201	16/01/2030	0.009	0.00328293									
202	17/01/2030	0.001	0.00432712									
203	18/01/2030	0.010	0.00532471									
204	19/01/2030	0.011	0.00661202									
205	20/01/2030	0.001	0.0081481									
206	21/01/2030	0.009	0.01041849									
207	22/01/2030	0.012	0.01214266									

AFTER. Inferior limit

Figure S9. Interval modification of formula "SUMAPRODUCTO" written in cell D192.

STEP 3:

In the down direction, extend the recently modified cell $D192$ to cover all the available $BE_{S,G}$ data (for this example, up to cell $D381$). Later, eliminate the content in any other cell in column D, as shown in Figure S10.

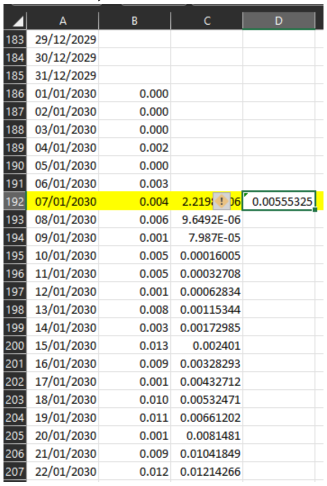

	A	B	C	D
183	29/12/2029			
184	30/12/2029			
185	31/12/2029			
186	01/01/2030	0.000		
187	02/01/2030	0.000		
188	03/01/2030	0.000		
189	04/01/2030	0.002		
190	05/01/2030	0.000		
191	06/01/2030	0.003		
192	07/01/2030	0.004	2.219[illegible]06	0.00555325
193	08/01/2030	0.006	9.6492E-06	
194	09/01/2030	0.001	7.987E-05	
195	10/01/2030	0.005	0.00016005	
196	11/01/2030	0.005	0.00032708	
197	12/01/2030	0.001	0.00062834	
198	13/01/2030	0.008	0.00115344	
199	14/01/2030	0.003	0.00172985	
200	15/01/2030	0.013	0.002401	
201	16/01/2030	0.009	0.00328293	
202	17/01/2030	0.001	0.00432712	
203	18/01/2030	0.010	0.00532471	
204	19/01/2030	0.011	0.00661202	
205	20/01/2030	0.001	0.0081481	
206	21/01/2030	0.009	0.01041849	
207	22/01/2030	0.012	0.01214266	

3.1 Select the cell $D192$.

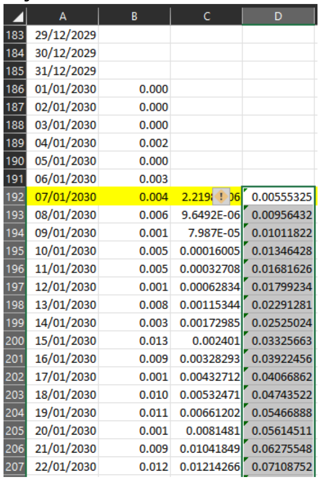

	A	B	C	D
183	29/12/2029			
184	30/12/2029			
185	31/12/2029			
186	01/01/2030	0.000		
187	02/01/2030	0.000		
188	03/01/2030	0.000		
189	04/01/2030	0.002		
190	05/01/2030	0.000		
191	06/01/2030	0.003		
192	07/01/2030	0.004	2.219[illegible]06	0.00555325
193	08/01/2030	0.006	9.6492E-06	0.00956432
194	09/01/2030	0.001	7.987E-05	0.01011822
195	10/01/2030	0.005	0.00016005	0.01346428
196	11/01/2030	0.005	0.00032708	0.01681626
197	12/01/2030	0.001	0.00062834	0.01799234
198	13/01/2030	0.008	0.00115344	0.02291281
199	14/01/2030	0.003	0.00172985	0.02525024
200	15/01/2030	0.013	0.002401	0.03325663
201	16/01/2030	0.009	0.00328293	0.03922456
202	17/01/2030	0.001	0.00432712	0.04066862
203	18/01/2030	0.010	0.00532471	0.04743522
204	19/01/2030	0.011	0.00661202	0.05466888
205	20/01/2030	0.001	0.0081481	0.05614511
206	21/01/2030	0.009	0.01041849	0.06275548
207	22/01/2030	0.012	0.01214266	0.07108752

3.2 Extend the selected cell.

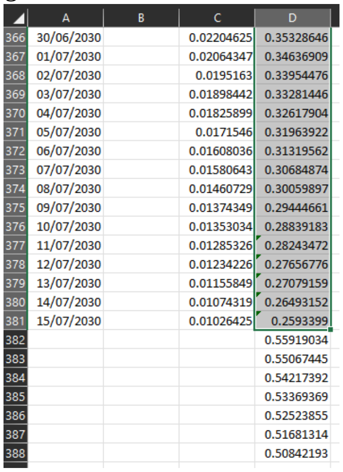

	A	B	C	D
366	30/06/2030		0.02204625	0.35328646
367	01/07/2030		0.02064347	0.34636909
368	02/07/2030		0.0195163	0.33954476
369	03/07/2030		0.01898442	0.33281446
370	04/07/2030		0.01825899	0.32617904
371	05/07/2030		0.0171546	0.31963922
372	06/07/2030		0.01608036	0.31319562
373	07/07/2030		0.01580643	0.30684874
374	08/07/2030		0.01460729	0.30059897
375	09/07/2030		0.01374349	0.29444661
376	10/07/2030		0.01353034	0.28839183
377	11/07/2030		0.01285326	0.28243472
378	12/07/2030		0.01234226	0.27656776
379	13/07/2030		0.01155849	0.27079159
380	14/07/2030		0.01074319	0.26493152
381	15/07/2030		0.01026425	0.2593399
382				0.55919034
383				0.55067445
384				0.54217392
385				0.53369369
386				0.52523855
387				0.51681314
388				0.50842193

3.3 Continue extending up to user data requires it; up to the last row of column C containing information.

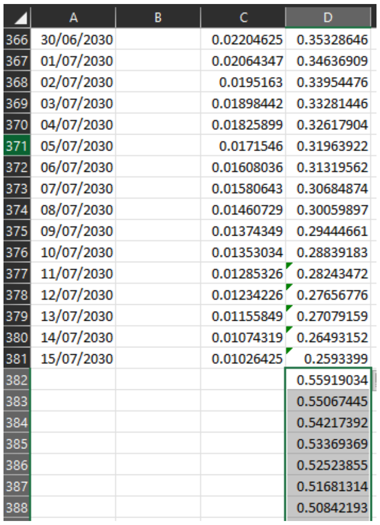

3.4 Select the data in column D placed below cell $D381$.

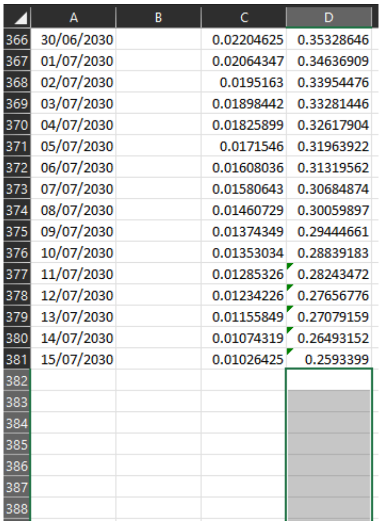

3.5 Eliminate the selected data (unnecessary data).

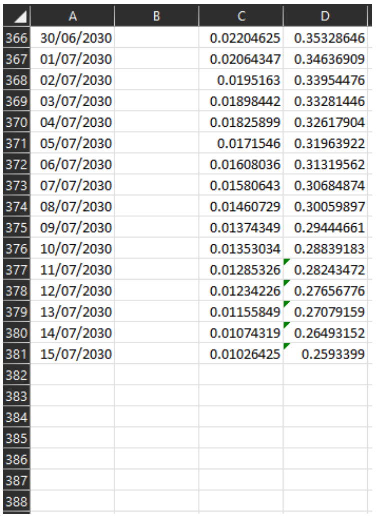

3.6 Final result.

Figure S10. Expansion of the formula in cell $D192$ along column D.

STEP 4:

Cut (Ctrl + X) the cell $F234$ and paste it in the yellow-marked cell of column F (for this example, cell $F192$), as shown in Figure S11.

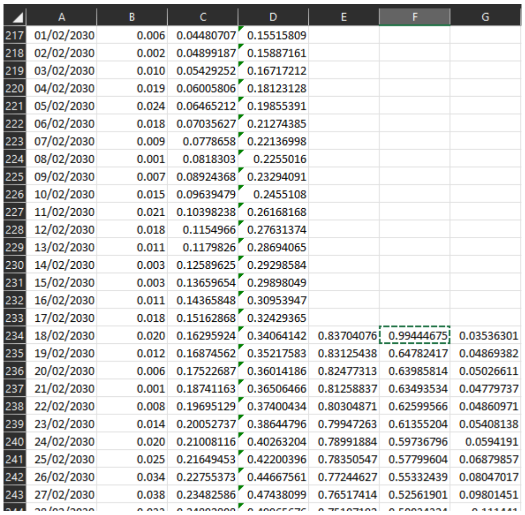

BEFORE. Cut (Ctrl+X) cell $F234$

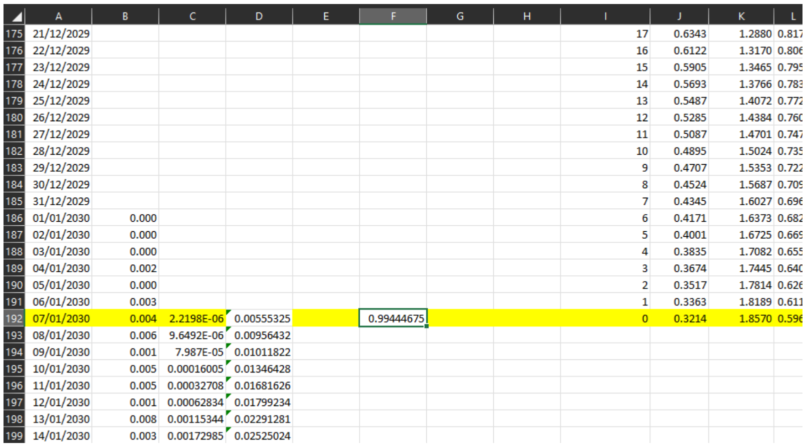

AFTER. Paste it in cell $F192$

Figure S11. Cutting of the cell F234 and its pasting in the yellow-marked cell of column F.

STEP 5:

Equivalently to **STEP 3**, extend, in the down direction, the recently modified cell $F192$, up to cover all the available $BE_{S,G}$ data (for this example, up to cell $F381$). Later, eliminate the remaining content in any other cell in the column, as shown in Figure S12.

	A	B	C	D	E	F
183	29/12/2029					
184	30/12/2029					
185	31/12/2029					
186	01/01/2030	0.000				
187	02/01/2030	0.000				
188	03/01/2030	0.000				
189	04/01/2030	0.002				
190	05/01/2030	0.000				
191	06/01/2030	0.003				
192	07/01/2030	0.004	2.2198E-06	0.00555325		0.99444675
193	08/01/2030	0.006	9.6492E-06	0.00956432		
194	09/01/2030	0.001	7.987E-05	0.01011822		
195	10/01/2030	0.005	0.00016005	0.01346428		
196	11/01/2030	0.005	0.00032708	0.01681626		
197	12/01/2030	0.001	0.00062834	0.01799234		
198	13/01/2030	0.008	0.00115344	0.02291281		
199	14/01/2030	0.003	0.00172985	0.02525024		
200	15/01/2030	0.013	0.002401	0.03325663		
201	16/01/2030	0.009	0.00328293	0.03922456		
202	17/01/2030	0.001	0.00432712	0.04066862		
203	18/01/2030	0.010	0.00532471	0.04743522		
204	19/01/2030	0.011	0.00661202	0.05466888		
205	20/01/2030	0.001	0.0081481	0.05614511		
206	21/01/2030	0.009	0.01041849	0.06275548		
207	22/01/2030	0.012	0.01214266	0.07108752		

5.1 Select cell $F192$.

	A	B	C	D	E	F
183	29/12/2029					
184	30/12/2029					
185	31/12/2029					
186	01/01/2030	0.000				
187	02/01/2030	0.000				
188	03/01/2030	0.000				
189	04/01/2030	0.002				
190	05/01/2030	0.000				
191	06/01/2030	0.003				
192	07/01/2030	0.004	2.2198E-06	0.00555325		0.99444675
193	08/01/2030	0.006	9.6492E-06	0.00956432		0.99043568
194	09/01/2030	0.001	7.987E-05	0.01011822		0.98988178
195	10/01/2030	0.005	0.00016005	0.01346428		0.98653572
196	11/01/2030	0.005	0.00032708	0.01681626		0.98318374
197	12/01/2030	0.001	0.00062834	0.01799234		0.98200766
198	13/01/2030	0.008	0.00115344	0.02291281		0.97708719
199	14/01/2030	0.003	0.00172985	0.02525024		0.97474976
200	15/01/2030	0.013	0.002401	0.03325663		0.96674337
201	16/01/2030	0.009	0.00328293	0.03922456		0.96077544
202	17/01/2030	0.001	0.00432712	0.04066862		0.95933138
203	18/01/2030	0.010	0.00532471	0.04743522		0.95256478
204	19/01/2030	0.011	0.00661202	0.05466888		0.94533112
205	20/01/2030	0.001	0.0081481	0.05614511		0.94385489
206	21/01/2030	0.009	0.01041849	0.06275548		0.93724452
207	22/01/2030	0.012	0.01214266	0.07108752		0.92891248

5.2 Extend the selected cell.

	A	B	C	D	E	F
366	30/06/2030		0.02204625	0.35328646	0.97795375	0.64671354
367	01/07/2030		0.02064347	0.34636909	0.97935653	0.65363091
368	02/07/2030		0.0195163	0.33954476	0.9804837	0.66045524
369	03/07/2030		0.01898442	0.33281446	0.98101558	0.66718554
370	04/07/2030		0.01825899	0.32617904	0.98174101	0.67382096
371	05/07/2030		0.0171546	0.31963922	0.9828454	0.68036078
372	06/07/2030		0.01608036	0.31319562	0.98391964	0.68680438
373	07/07/2030		0.01580643	0.30684874	0.98419357	0.69315126
374	08/07/2030		0.01460729	0.30059897	0.98539271	0.69940103
375	09/07/2030		0.01374349	0.29444661	0.98625651	0.70555339
376	10/07/2030		0.01353034	0.28839183	0.98646966	0.71160817
377	11/07/2030		0.01285326	0.28243472	0.98714674	0.71756528
378	12/07/2030		0.01234226	0.27656776	0.98765774	0.72343224
379	13/07/2030		0.01155849	0.27079159	0.98844151	0.72920841
380	14/07/2030		0.01074319	0.26493152	0.98925681	0.73506848
381	15/07/2030		0.01026425	0.2593399	0.98973575	0.7406601
382					1	1
383					1	1
384					1	1
385					1	1
386					1	1
387					1	1
388					1	1

5.3 Select the data in column F placed below cell $F381$.

	A	B	C	D	E	F
366	30/06/2030		0.02204625	0.35328646	0.97795375	0.64671354
367	01/07/2030		0.02064347	0.34636909	0.97935653	0.65363091
368	02/07/2030		0.0195163	0.33954476	0.9804837	0.66045524
369	03/07/2030		0.01898442	0.33281446	0.98101558	0.66718554
370	04/07/2030		0.01825899	0.32617904	0.98174101	0.67382096
371	05/07/2030		0.0171546	0.31963922	0.9828454	0.68036078
372	06/07/2030		0.01608036	0.31319562	0.98391964	0.68680438
373	07/07/2030		0.01580643	0.30684874	0.98419357	0.69315126
374	08/07/2030		0.01460729	0.30059897	0.98539271	0.69940103
375	09/07/2030		0.01374349	0.29444661	0.98625651	0.70555339
376	10/07/2030		0.01353034	0.28839183	0.98646966	0.71160817
377	11/07/2030		0.01285326	0.28243472	0.98714674	0.71756528
378	12/07/2030		0.01234226	0.27656776	0.98765774	0.72343224
379	13/07/2030		0.01155849	0.27079159	0.98844151	0.72920841
380	14/07/2030		0.01074319	0.26493152	0.98925681	0.73506848
381	15/07/2030		0.01026425	0.2593399	0.98973575	0.7406601
382					1	
383					1	
384					1	
385					1	
386					1	
387					1	
388					1	

5.4 Eliminate such data (unnecessary data).

Figure S12. Expansion of the formula in cell $F192$ along column F.

STEP 6:

Extend the cell $E234$ (the first cell containing information in column E) up to the yellow-marked cell in column E (for this example, cell $E192$). Later, erase the content in column E below cell E381, as shown in Figure S13.

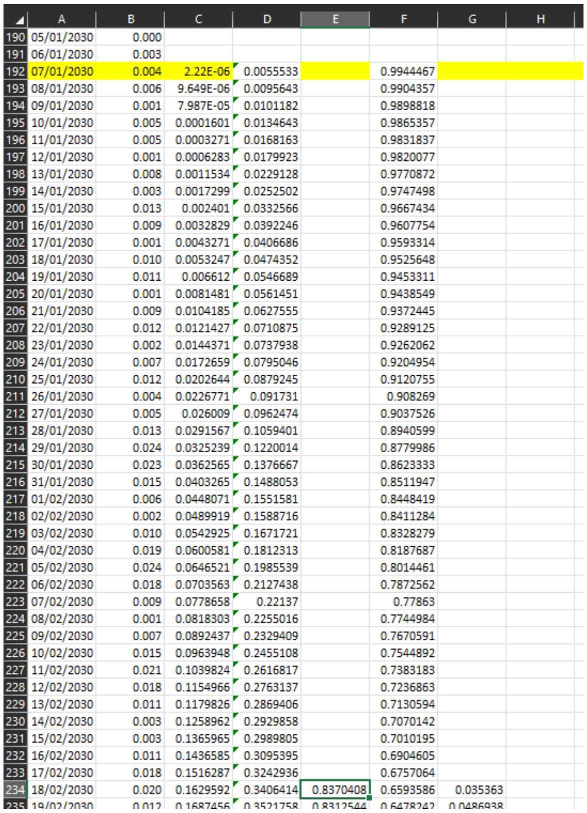

	A	B	C	D	E	F	G	H
190	05/01/2030	0.000						
191	06/01/2030	0.003						
192	07/01/2030	0.004	2.22E-06	0.0055533		0.9944467		
193	08/01/2030	0.006	9.649E-06	0.0095643		0.9904357		
194	09/01/2030	0.001	7.987E-05	0.0101182		0.9898818		
195	10/01/2030	0.005	0.0001601	0.0134643		0.9865357		
196	11/01/2030	0.005	0.0003271	0.0168163		0.9831837		
197	12/01/2030	0.001	0.0006283	0.0179923		0.9820077		
198	13/01/2030	0.008	0.0011534	0.0229128		0.9770872		
199	14/01/2030	0.003	0.0017299	0.0252502		0.9747498		
200	15/01/2030	0.013	0.002401	0.0332566		0.9667434		
201	16/01/2030	0.009	0.0032829	0.0392246		0.9607754		
202	17/01/2030	0.001	0.0043271	0.0406686		0.9593314		
203	18/01/2030	0.010	0.0053247	0.0474352		0.9525648		
204	19/01/2030	0.011	0.006612	0.0546689		0.9453311		
205	20/01/2030	0.001	0.0081481	0.0561451		0.9438549		
206	21/01/2030	0.009	0.0104185	0.0627555		0.9372445		
207	22/01/2030	0.012	0.0121427	0.0710875		0.9289125		
208	23/01/2030	0.002	0.0144371	0.0737938		0.9262062		
209	24/01/2030	0.007	0.0172659	0.0795046		0.9204954		
210	25/01/2030	0.012	0.0202644	0.0879245		0.9120755		
211	26/01/2030	0.004	0.0226771	0.091731		0.908269		
212	27/01/2030	0.005	0.026009	0.0962474		0.9037526		
213	28/01/2030	0.013	0.0291567	0.1059401		0.8940599		
214	29/01/2030	0.024	0.0325239	0.1220014		0.8779986		
215	30/01/2030	0.023	0.0362565	0.1376667		0.8623333		
216	31/01/2030	0.015	0.0403265	0.1488053		0.8511947		
217	01/02/2030	0.006	0.0448071	0.1551581		0.8448419		
218	02/02/2030	0.002	0.0489919	0.1588716		0.8411284		
219	03/02/2030	0.010	0.0542925	0.1671721		0.8328279		
220	04/02/2030	0.019	0.0600581	0.1812313		0.8187687		
221	05/02/2030	0.024	0.0646521	0.1985539		0.8014461		
222	06/02/2030	0.018	0.0703563	0.2127438		0.7872562		
223	07/02/2030	0.009	0.0778658	0.22137		0.77863		
224	08/02/2030	0.001	0.0818303	0.2255016		0.7744984		
225	09/02/2030	0.007	0.0892437	0.2329409		0.7670591		
226	10/02/2030	0.015	0.0963948	0.2455108		0.7544892		
227	11/02/2030	0.021	0.1039824	0.2616817		0.7383183		
228	12/02/2030	0.018	0.1154966	0.2763137		0.7236863		
229	13/02/2030	0.011	0.1179826	0.2869406		0.7130594		
230	14/02/2030	0.003	0.1258962	0.2929858		0.7070142		
231	15/02/2030	0.003	0.1365965	0.2989805		0.7010195		
232	16/02/2030	0.011	0.1436585	0.3095395		0.6904605		
233	17/02/2030	0.018	0.1516287	0.3242936		0.6757064		
234	18/02/2030	0.020	0.1629592	0.3406414	0.8370408	0.6593586	0.035363	
235	19/02/2030	0.012	0.1687456	0.3521758	0.8312544	0.6478242	0.0486938	

6.1 Select cell $E234$.

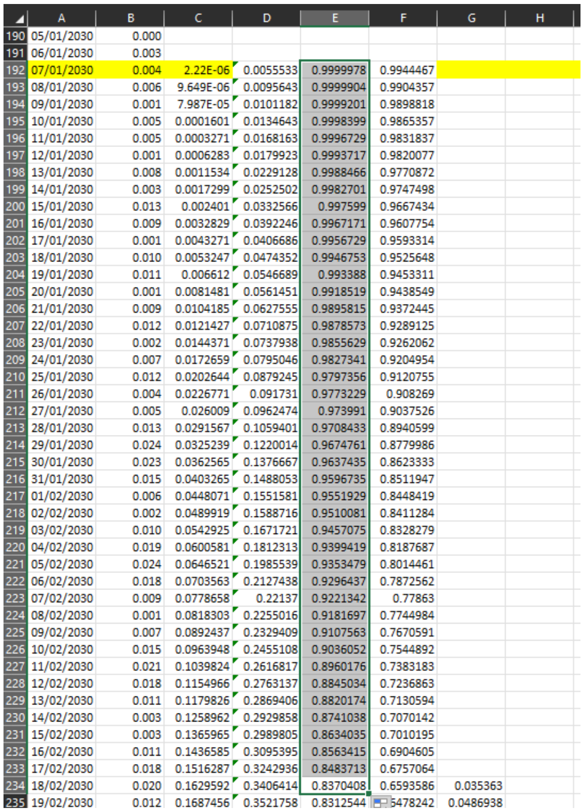

	A	B	C	D	E	F	G	H
190	05/01/2030	0.000						
191	06/01/2030	0.003						
192	07/01/2030	0.004	2.22E-06	0.0055533	0.9999978	0.9944467		
193	08/01/2030	0.006	9.649E-06	0.0095643	0.9999904	0.9904357		
194	09/01/2030	0.001	7.987E-05	0.0101182	0.9999201	0.9898818		
195	10/01/2030	0.005	0.0001601	0.0134643	0.9998399	0.9865357		
196	11/01/2030	0.005	0.0003271	0.0168163	0.9996729	0.9831837		
197	12/01/2030	0.001	0.0006283	0.0179923	0.9993717	0.9820077		
198	13/01/2030	0.008	0.0011534	0.0229128	0.9988466	0.9770872		
199	14/01/2030	0.003	0.0017299	0.0252502	0.9982701	0.9747498		
200	15/01/2030	0.013	0.002401	0.0332566	0.997599	0.9667434		
201	16/01/2030	0.009	0.0032829	0.0392246	0.9967171	0.9607754		
202	17/01/2030	0.001	0.0043271	0.0406686	0.9956729	0.9593314		
203	18/01/2030	0.010	0.0053247	0.0474352	0.9946753	0.9525648		
204	19/01/2030	0.011	0.006612	0.0546689	0.993388	0.9453311		
205	20/01/2030	0.001	0.0081481	0.0561451	0.9918519	0.9438549		
206	21/01/2030	0.009	0.0104185	0.0627555	0.9895815	0.9372445		
207	22/01/2030	0.012	0.0121427	0.0710875	0.9878573	0.9289125		
208	23/01/2030	0.002	0.0144371	0.0737938	0.9855629	0.9262062		
209	24/01/2030	0.007	0.0172659	0.0795046	0.9827341	0.9204954		
210	25/01/2030	0.012	0.0202644	0.0879245	0.9797356	0.9120755		
211	26/01/2030	0.004	0.0226771	0.091731	0.9773229	0.908269		
212	27/01/2030	0.005	0.026009	0.0962474	0.973991	0.9037526		
213	28/01/2030	0.013	0.0291567	0.1059401	0.9708433	0.8940599		
214	29/01/2030	0.024	0.0325239	0.1220014	0.9674761	0.8779986		
215	30/01/2030	0.023	0.0362565	0.1376667	0.9637435	0.8623333		
216	31/01/2030	0.015	0.0403265	0.1488053	0.9596735	0.8511947		
217	01/02/2030	0.006	0.0448071	0.1551581	0.9551929	0.8448419		
218	02/02/2030	0.002	0.0489919	0.1588716	0.9510081	0.8411284		
219	03/02/2030	0.010	0.0542925	0.1671721	0.9457075	0.8328279		
220	04/02/2030	0.019	0.0600581	0.1812313	0.9399419	0.8187687		
221	05/02/2030	0.024	0.0646521	0.1985539	0.9353479	0.8014461		
222	06/02/2030	0.018	0.0703563	0.2127438	0.9296437	0.7872562		
223	07/02/2030	0.009	0.0778658	0.22137	0.9221342	0.77863		
224	08/02/2030	0.001	0.0818303	0.2255016	0.9181697	0.7744984		
225	09/02/2030	0.007	0.0892437	0.2329409	0.9107563	0.7670591		
226	10/02/2030	0.015	0.0963948	0.2455108	0.9036052	0.7544892		
227	11/02/2030	0.021	0.1039824	0.2616817	0.8960176	0.7383183		
228	12/02/2030	0.018	0.1154966	0.2763137	0.8845034	0.7236863		
229	13/02/2030	0.011	0.1179826	0.2869406	0.8820174	0.7130594		
230	14/02/2030	0.003	0.1258962	0.2929858	0.8741038	0.7070142		
231	15/02/2030	0.003	0.1365965	0.2989805	0.8634035	0.7010195		
232	16/02/2030	0.011	0.1436585	0.3095395	0.8563415	0.6904605		
233	17/02/2030	0.018	0.1516287	0.3242936	0.8483713	0.6757064		
234	18/02/2030	0.020	0.1629592	0.3406414	0.8370408	0.6593586	0.035363	
235	19/02/2030	0.012	0.1687456	0.3521758	0.8312544	[illegible]478242	0.0486938	

6.2 Extend the selected cell.

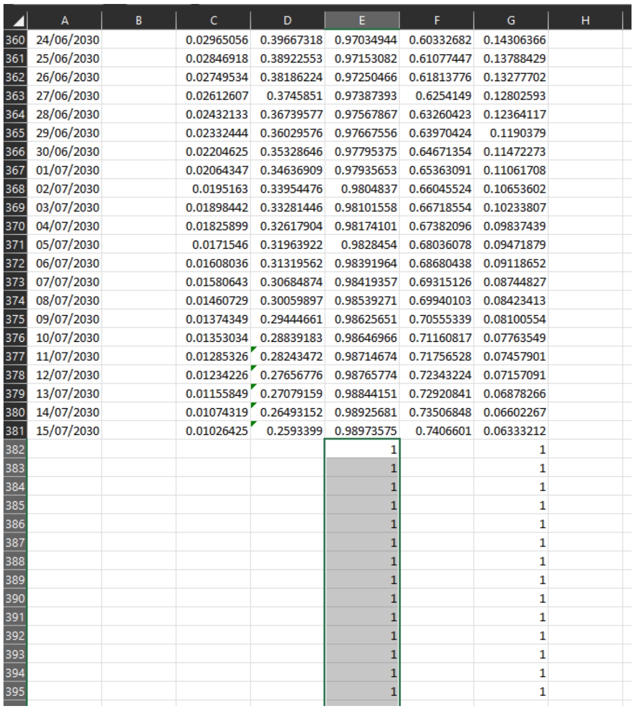

	A	B	C	D	E	F	G	H
360	24/06/2030		0.02965056	0.39667318	0.97034944	0.60332682	0.14306366	
361	25/06/2030		0.02846918	0.38922553	0.97153082	0.61077447	0.13788429	
362	26/06/2030		0.02749534	0.38186224	0.97250466	0.61813776	0.13277702	
363	27/06/2030		0.02612607	0.3745851	0.97387393	0.6254149	0.12802593	
364	28/06/2030		0.02432133	0.36739577	0.97567867	0.63260423	0.12364117	
365	29/06/2030		0.02332444	0.36029576	0.97667556	0.63970424	0.1190379	
366	30/06/2030		0.02204625	0.35328646	0.97795375	0.64671354	0.11472273	
367	01/07/2030		0.02064347	0.34636909	0.97935653	0.65363091	0.11061708	
368	02/07/2030		0.0195163	0.33954476	0.9804837	0.66045524	0.10653602	
369	03/07/2030		0.01898442	0.33281446	0.98101558	0.66718554	0.10233807	
370	04/07/2030		0.01825899	0.32617904	0.98174101	0.67382096	0.09837439	
371	05/07/2030		0.0171546	0.31963922	0.9828454	0.68036078	0.09471879	
372	06/07/2030		0.01608036	0.31319562	0.98391964	0.68680438	0.09118652	
373	07/07/2030		0.01580643	0.30684874	0.98419357	0.69315126	0.08744827	
374	08/07/2030		0.01460729	0.30059897	0.98539271	0.69940103	0.08423413	
375	09/07/2030		0.01374349	0.29444661	0.98625651	0.70555339	0.08100554	
376	10/07/2030		0.01353034	0.28839183	0.98646966	0.71160817	0.07763549	
377	11/07/2030		0.01285326	0.28243472	0.98714674	0.71756528	0.07457901	
378	12/07/2030		0.01234226	0.27656776	0.98765774	0.72343224	0.07157091	
379	13/07/2030		0.01155849	0.27079159	0.98844151	0.72920841	0.06878266	
380	14/07/2030		0.01074319	0.26493152	0.98925681	0.73506848	0.06602267	
381	15/07/2030		0.01026425	0.2593399	0.98973575	0.7406601	0.06333212	
382					1		1	
383					1		1	
384					1		1	
385					1		1	
386					1		1	
387					1		1	
388					1		1	
389					1		1	
390					1		1	
391					1		1	
392					1		1	
393					1		1	
394					1		1	
395					1		1	

6.3 Select the data in column E placed below cell E381.

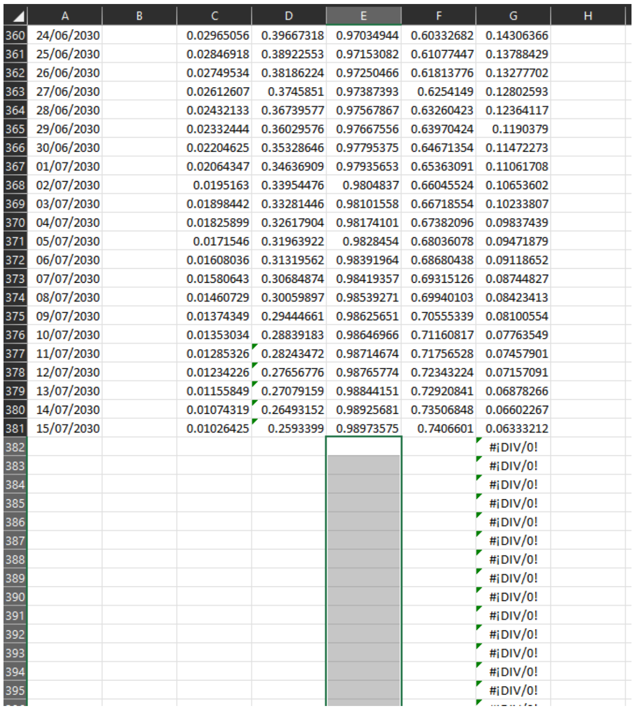

	A	B	C	D	E	F	G	H
360	24/06/2030		0.02965056	0.39667318	0.97034944	0.60332682	0.14306366	
361	25/06/2030		0.02846918	0.38922553	0.97153082	0.61077447	0.13788429	
362	26/06/2030		0.02749534	0.38186224	0.97250466	0.61813776	0.13277702	
363	27/06/2030		0.02612607	0.3745851	0.97387393	0.6254149	0.12802593	
364	28/06/2030		0.02432133	0.36739577	0.97567867	0.63260423	0.12364117	
365	29/06/2030		0.02332444	0.36029576	0.97667556	0.63970424	0.1190379	
366	30/06/2030		0.02204625	0.35328646	0.97795375	0.64671354	0.11472273	
367	01/07/2030		0.02064347	0.34636909	0.97935653	0.65363091	0.11061708	
368	02/07/2030		0.0195163	0.33954476	0.9804837	0.66045524	0.10653602	
369	03/07/2030		0.01898442	0.33281446	0.98101558	0.66718554	0.10233807	
370	04/07/2030		0.01825899	0.32617904	0.98174101	0.67382096	0.09837439	
371	05/07/2030		0.0171546	0.31963922	0.9828454	0.68036078	0.09471879	
372	06/07/2030		0.01608036	0.31319562	0.98391964	0.68680438	0.09118652	
373	07/07/2030		0.01580643	0.30684874	0.98419357	0.69315126	0.08744827	
374	08/07/2030		0.01460729	0.30059897	0.98539271	0.69940103	0.08423413	
375	09/07/2030		0.01374349	0.29444661	0.98625651	0.70555339	0.08100554	
376	10/07/2030		0.01353034	0.28839183	0.98646966	0.71160817	0.07763549	
377	11/07/2030		0.01285326	0.28243472	0.98714674	0.71756528	0.07457901	
378	12/07/2030		0.01234226	0.27656776	0.98765774	0.72343224	0.07157091	
379	13/07/2030		0.01155849	0.27079159	0.98844151	0.72920841	0.06878266	
380	14/07/2030		0.01074319	0.26493152	0.98925681	0.73506848	0.06602267	
381	15/07/2030		0.01026425	0.2593399	0.98973575	0.7406601	0.06333212	
382							#¡DIV/0!	
383							#¡DIV/0!	
384							#¡DIV/0!	
385							#¡DIV/0!	
386							#¡DIV/0!	
387							#¡DIV/0!	
388							#¡DIV/0!	
389							#¡DIV/0!	
390							#¡DIV/0!	
391							#¡DIV/0!	
392							#¡DIV/0!	
393							#¡DIV/0!	
394							#¡DIV/0!	
395							#¡DIV/0!	

6.4 Eliminate the selected data (unnecessary data).

Figure S13. Extension of the formula contained in cell E234 and elimination of unnecessary data

STEP 7:

Write in the yellow cell of column G, the formula shown in Figure S14.

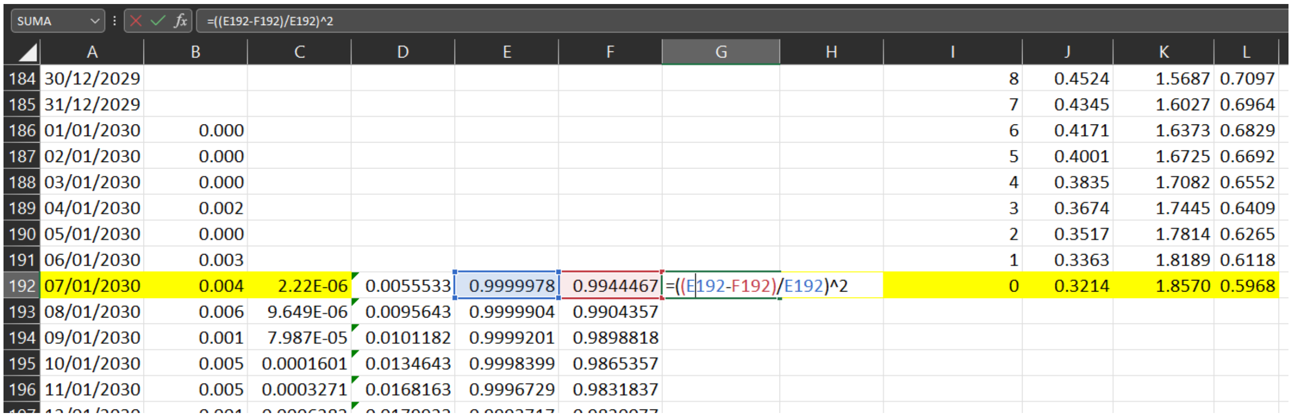

SUMA | =((E192-F192)/E192)^2

	A	B	C	D	E	F	G	H	I	J	K	L
184	30/12/2029								8	0.4524	1.5687	0.7097
185	31/12/2029								7	0.4345	1.6027	0.6964
186	01/01/2030	0.000							6	0.4171	1.6373	0.6829
187	02/01/2030	0.000							5	0.4001	1.6725	0.6692
188	03/01/2030	0.000							4	0.3835	1.7082	0.6552
189	04/01/2030	0.002							3	0.3674	1.7445	0.6409
190	05/01/2030	0.000							2	0.3517	1.7814	0.6265
191	06/01/2030	0.003							1	0.3363	1.8189	0.6118
192	07/01/2030	0.004	2.22E-06	0.0055533	0.9999978	0.9944467	=((E192-F192)/E192)^2		0	0.3214	1.8570	0.5968
193	08/01/2030	0.006	9.649E-06	0.0095643	0.9999904	0.9904357						
194	09/01/2030	0.001	7.987E-05	0.0101182	0.9999201	0.9898818						
195	10/01/2030	0.005	0.0001601	0.0134643	0.9998399	0.9865357						
196	11/01/2030	0.005	0.0003271	0.0168163	0.9996729	0.9831837						
[illegible]	[illegible]	[illegible]	[illegible]	[illegible]	[illegible]	[illegible]						

Figure S14. Insertion of the formula for the square deviation in the yellow-marked cell of column G.

STEP 8:

Extend the cell $G192$ down along column G (for this example, up to cell $G381$). Later, eliminate the remaining content in any other cell in column G, as shown in Figure S15.

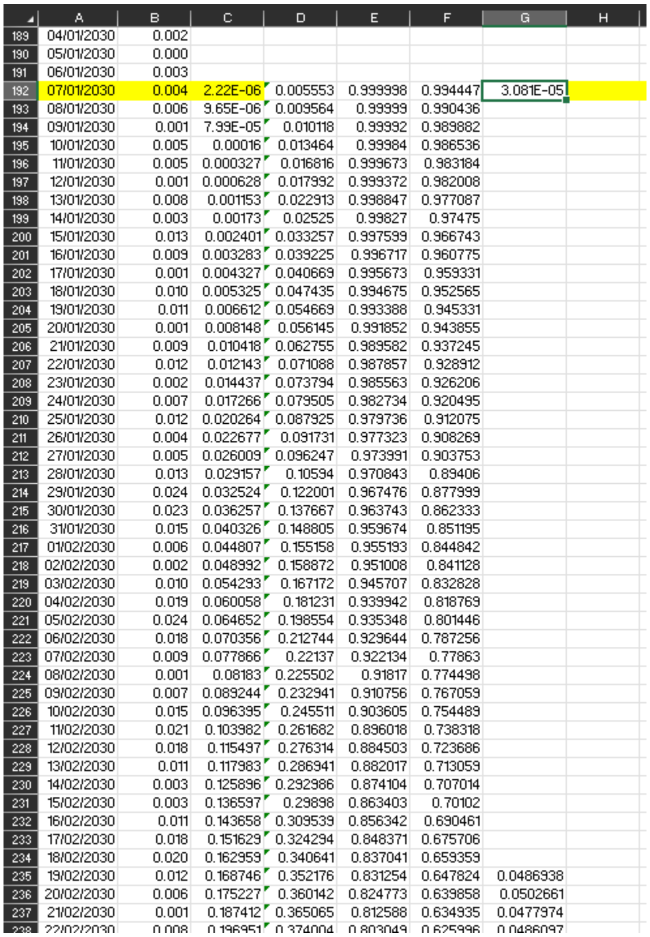

	A	B	C	D	E	F	G	H
189	04/01/2030	0.002						
190	05/01/2030	0.000						
191	06/01/2030	0.003						
192	07/01/2030	0.004	2.22E-06	0.005553	0.999998	0.994447	3.081E-05	
193	08/01/2030	0.006	9.65E-06	0.009564	0.99999	0.990436		
194	09/01/2030	0.001	7.99E-05	0.010118	0.99992	0.989882		
195	10/01/2030	0.005	0.00016	0.013464	0.99984	0.986536		
196	11/01/2030	0.005	0.000327	0.016816	0.999673	0.983184		
197	12/01/2030	0.001	0.000628	0.017992	0.999372	0.982008		
198	13/01/2030	0.008	0.001153	0.022913	0.998847	0.977087		
199	14/01/2030	0.003	0.00173	0.02525	0.99827	0.97475		
200	15/01/2030	0.013	0.002401	0.033257	0.997599	0.966743		
201	16/01/2030	0.009	0.003283	0.039225	0.996717	0.960775		
202	17/01/2030	0.001	0.004327	0.040669	0.995673	0.959331		
203	18/01/2030	0.010	0.005325	0.047435	0.994675	0.952565		
204	19/01/2030	0.011	0.006612	0.054669	0.993388	0.945331		
205	20/01/2030	0.001	0.008148	0.056145	0.991852	0.943855		
206	21/01/2030	0.009	0.010418	0.062755	0.989582	0.937245		
207	22/01/2030	0.012	0.012143	0.071088	0.987857	0.928912		
208	23/01/2030	0.002	0.014437	0.073794	0.985563	0.926206		
209	24/01/2030	0.007	0.017266	0.079505	0.982734	0.920495		
210	25/01/2030	0.012	0.020264	0.087925	0.979736	0.912075		
211	26/01/2030	0.004	0.022677	0.091731	0.977323	0.908269		
212	27/01/2030	0.005	0.026009	0.096247	0.973991	0.903753		
213	28/01/2030	0.013	0.029157	0.10594	0.970843	0.89406		
214	29/01/2030	0.024	0.032524	0.122001	0.967476	0.877999		
215	30/01/2030	0.023	0.036257	0.137667	0.963743	0.862333		
216	31/01/2030	0.015	0.040326	0.148805	0.959674	0.851195		
217	01/02/2030	0.006	0.044807	0.155158	0.955193	0.844842		
218	02/02/2030	0.002	0.048992	0.158872	0.951008	0.841128		
219	03/02/2030	0.010	0.054293	0.167172	0.945707	0.832828		
220	04/02/2030	0.019	0.060058	0.181231	0.939942	0.818769		
221	05/02/2030	0.024	0.064652	0.198554	0.935348	0.801446		
222	06/02/2030	0.018	0.070356	0.212744	0.929644	0.787256		
223	07/02/2030	0.009	0.077866	0.22137	0.922134	0.77863		
224	08/02/2030	0.001	0.08183	0.225502	0.91817	0.774498		
225	09/02/2030	0.007	0.089244	0.232941	0.910756	0.767059		
226	10/02/2030	0.015	0.096395	0.245511	0.903605	0.754489		
227	11/02/2030	0.021	0.103982	0.261682	0.896018	0.738318		
228	12/02/2030	0.018	0.115497	0.276314	0.884503	0.723686		
229	13/02/2030	0.011	0.117983	0.286941	0.882017	0.713059		
230	14/02/2030	0.003	0.125896	0.292986	0.874104	0.707014		
231	15/02/2030	0.003	0.136597	0.29898	0.863403	0.70102		
232	16/02/2030	0.011	0.143658	0.309539	0.856342	0.690461		
233	17/02/2030	0.018	0.151629	0.324294	0.848371	0.675706		
234	18/02/2030	0.020	0.162959	0.340641	0.837041	0.659359		
235	19/02/2030	0.012	0.168746	0.352176	0.831254	0.647824	0.0486938	
236	20/02/2030	0.006	0.175227	0.360142	0.824773	0.639858	0.0502661	
237	21/02/2030	0.001	0.187412	0.365065	0.812588	0.634935	0.0477974	
238	22/02/2030	0.008	0.196951	0.374004	0.803049	0.625996	0.0486097	

7.1 Select cell $G192$.

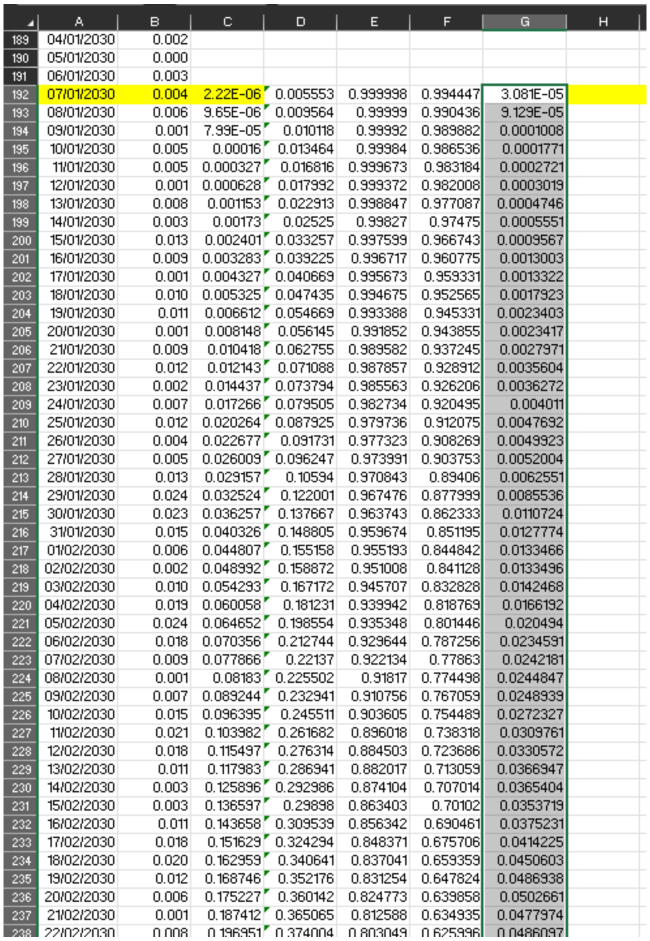

	A	B	C	D	E	F	G	H
189	04/01/2030	0.002						
190	05/01/2030	0.000						
191	06/01/2030	0.003						
192	07/01/2030	0.004	2.22E-06	0.005553	0.999998	0.994447	3.081E-05	
193	08/01/2030	0.006	9.65E-06	0.009564	0.99999	0.990436	9.129E-05	
194	09/01/2030	0.001	7.99E-05	0.010118	0.99992	0.989882	0.0001008	
195	10/01/2030	0.005	0.00016	0.013464	0.99984	0.986536	0.0001771	
196	11/01/2030	0.005	0.000327	0.016816	0.999673	0.983184	0.0002721	
197	12/01/2030	0.001	0.000628	0.017992	0.999372	0.982008	0.0003019	
198	13/01/2030	0.008	0.001153	0.022913	0.998847	0.977087	0.0004746	
199	14/01/2030	0.003	0.00173	0.02525	0.99827	0.97475	0.0005551	
200	15/01/2030	0.013	0.002401	0.033257	0.997599	0.966743	0.0009567	
201	16/01/2030	0.009	0.003283	0.039225	0.996717	0.960775	0.0013003	
202	17/01/2030	0.001	0.004327	0.040669	0.995673	0.959331	0.0013322	
203	18/01/2030	0.010	0.005325	0.047435	0.994675	0.952565	0.0017923	
204	19/01/2030	0.011	0.006612	0.054669	0.993388	0.945331	0.0023403	
205	20/01/2030	0.001	0.008148	0.056145	0.991852	0.943855	0.0023417	
206	21/01/2030	0.009	0.010418	0.062755	0.989582	0.937245	0.0027971	
207	22/01/2030	0.012	0.012143	0.071088	0.987857	0.928912	0.0035604	
208	23/01/2030	0.002	0.014437	0.073794	0.985563	0.926206	0.0036272	
209	24/01/2030	0.007	0.017266	0.079505	0.982734	0.920495	0.004011	
210	25/01/2030	0.012	0.020264	0.087925	0.979736	0.912075	0.0047692	
211	26/01/2030	0.004	0.022677	0.091731	0.977323	0.908269	0.0049923	
212	27/01/2030	0.005	0.026009	0.096247	0.973991	0.903753	0.0052004	
213	28/01/2030	0.013	0.029157	0.10594	0.970843	0.89406	0.0062551	
214	29/01/2030	0.024	0.032524	0.122001	0.967476	0.877999	0.0085536	
215	30/01/2030	0.023	0.036257	0.137667	0.963743	0.862333	0.0110724	
216	31/01/2030	0.015	0.040326	0.148805	0.959674	0.851195	0.0127774	
217	01/02/2030	0.006	0.044807	0.155158	0.955193	0.844842	0.0133466	
218	02/02/2030	0.002	0.048992	0.158872	0.951008	0.841128	0.0133496	
219	03/02/2030	0.010	0.054293	0.167172	0.945707	0.832828	0.0142468	
220	04/02/2030	0.019	0.060058	0.181231	0.939942	0.818769	0.0166192	
221	05/02/2030	0.024	0.064652	0.198554	0.935348	0.801446	0.020494	
222	06/02/2030	0.018	0.070356	0.212744	0.929644	0.787256	0.0234591	
223	07/02/2030	0.009	0.077866	0.22137	0.922134	0.77863	0.0242181	
224	08/02/2030	0.001	0.08183	0.225502	0.91817	0.774498	0.0244847	
225	09/02/2030	0.007	0.089244	0.232941	0.910756	0.767059	0.0248939	
226	10/02/2030	0.015	0.096395	0.245511	0.903605	0.754489	0.0272327	
227	11/02/2030	0.021	0.103982	0.261682	0.896018	0.738318	0.0309761	
228	12/02/2030	0.018	0.115497	0.276314	0.884503	0.723686	0.0330572	
229	13/02/2030	0.011	0.117983	0.286941	0.882017	0.713059	0.0366947	
230	14/02/2030	0.003	0.125896	0.292986	0.874104	0.707014	0.0365404	
231	15/02/2030	0.003	0.136597	0.29898	0.863403	0.70102	0.0353719	
232	16/02/2030	0.011	0.143658	0.309539	0.856342	0.690461	0.0375231	
233	17/02/2030	0.018	0.151629	0.324294	0.848371	0.675706	0.0414225	
234	18/02/2030	0.020	0.162959	0.340641	0.837041	0.659359	0.0450603	
235	19/02/2030	0.012	0.168746	0.352176	0.831254	0.647824	0.0486938	
236	20/02/2030	0.006	0.175227	0.360142	0.824773	0.639858	0.0502661	
237	21/02/2030	0.001	0.187412	0.365065	0.812588	0.634935	0.0477974	
238	22/02/2030	0.008	0.196951	0.374004	0.803049	0.625996	0.0486097	

7.2 Extend selected cell.

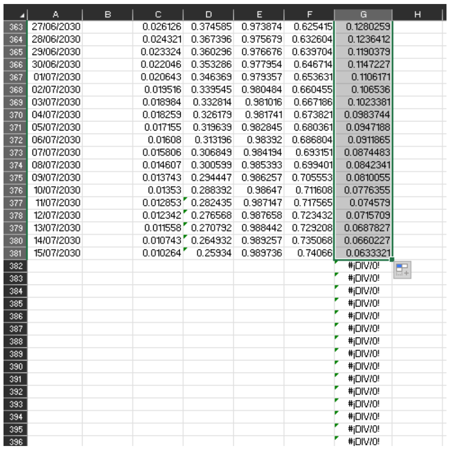

	A	B	C	D	E	F	G	H
363	27/06/2030		0.026126	0.374585	0.973874	0.625415	0.1280259	
364	28/06/2030		0.024321	0.367396	0.975679	0.632604	0.1236412	
365	29/06/2030		0.023324	0.360296	0.976676	0.639704	0.1190379	
366	30/06/2030		0.022046	0.353286	0.977954	0.646714	0.1147227	
367	01/07/2030		0.020643	0.346369	0.979357	0.653631	0.1106171	
368	02/07/2030		0.019516	0.339545	0.980484	0.660455	0.106536	
369	03/07/2030		0.018984	0.332814	0.981016	0.667186	0.1023381	
370	04/07/2030		0.018259	0.326179	0.981741	0.673821	0.0983744	
371	05/07/2030		0.017155	0.319639	0.982845	0.680361	0.0947188	
372	06/07/2030		0.01608	0.313196	0.98392	0.686804	0.0911865	
373	07/07/2030		0.015806	0.306849	0.984194	0.693151	0.0874483	
374	08/07/2030		0.014607	0.300599	0.985393	0.699401	0.0842341	
375	09/07/2030		0.013743	0.294447	0.986257	0.705553	0.0810055	
376	10/07/2030		0.01353	0.288392	0.98647	0.711608	0.0776355	
377	11/07/2030		0.012853	0.282435	0.987147	0.717565	0.074579	
378	12/07/2030		0.012342	0.276568	0.987658	0.723432	0.0715709	
379	13/07/2030		0.011558	0.270792	0.988442	0.729208	0.0687827	
380	14/07/2030		0.010743	0.264932	0.989257	0.735068	0.0660227	
381	15/07/2030		0.010264	0.25934	0.989736	0.74066	0.0633321	
382							#¡DIV/0!	
383							#¡DIV/0!	
384							#¡DIV/0!	
385							#¡DIV/0!	
386							#¡DIV/0!	
387							#¡DIV/0!	
388							#¡DIV/0!	
389							#¡DIV/0!	
390							#¡DIV/0!	
391							#¡DIV/0!	
392							#¡DIV/0!	
393							#¡DIV/0!	
394							#¡DIV/0!	
395							#¡DIV/0!	
396							#¡DIV/0!	

7.3 Continue the extension up to cell $G381$.

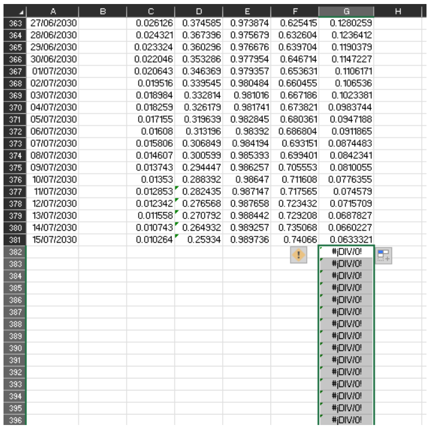

	A	B	C	D	E	F	G	H
363	27/06/2030		0.026126	0.374585	0.973874	0.625415	0.1280259	
364	28/06/2030		0.024321	0.367396	0.975679	0.632604	0.1236412	
365	29/06/2030		0.023324	0.360296	0.976676	0.639704	0.1190379	
366	30/06/2030		0.022046	0.353286	0.977954	0.646714	0.1147227	
367	01/07/2030		0.020643	0.346369	0.979357	0.653631	0.1106171	
368	02/07/2030		0.019516	0.339545	0.980484	0.660455	0.106536	
369	03/07/2030		0.018984	0.332814	0.981016	0.667186	0.1023381	
370	04/07/2030		0.018259	0.326179	0.981741	0.673821	0.0983744	
371	05/07/2030		0.017155	0.319639	0.982845	0.680361	0.0947188	
372	06/07/2030		0.01608	0.313196	0.98392	0.686804	0.0911865	
373	07/07/2030		0.015806	0.306849	0.984194	0.693151	0.0874483	
374	08/07/2030		0.014607	0.300599	0.985393	0.699401	0.0842341	
375	09/07/2030		0.013743	0.294447	0.986257	0.705553	0.0810055	
376	10/07/2030		0.01353	0.288392	0.98647	0.711608	0.0776355	
377	11/07/2030		0.012853	0.282435	0.987147	0.717565	0.074579	
378	12/07/2030		0.012342	0.276568	0.987658	0.723432	0.0715709	
379	13/07/2030		0.011558	0.270792	0.988442	0.729208	0.0687827	
380	14/07/2030		0.010743	0.264932	0.989257	0.735068	0.0660227	
381	15/07/2030		0.010264	0.25934	0.989736	0.74066	0.0633321	
382							#¡DIV/0!	
383							#¡DIV/0!	
384							#¡DIV/0!	
385							#¡DIV/0!	
386							#¡DIV/0!	
387							#¡DIV/0!	
388							#¡DIV/0!	
389							#¡DIV/0!	
390							#¡DIV/0!	
391							#¡DIV/0!	
392							#¡DIV/0!	
393							#¡DIV/0!	
394							#¡DIV/0!	
395							#¡DIV/0!	
396							#¡DIV/0!	

7.4 Select the data in column G placed below cell $G381$.

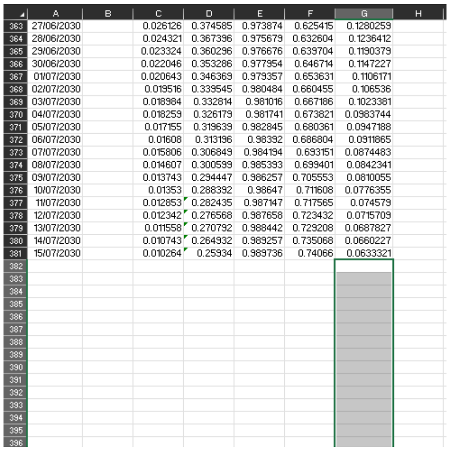

	A	B	C	D	E	F	G	H
363	27/06/2030		0.026126	0.374585	0.973874	0.625415	0.1280259	
364	28/06/2030		0.024321	0.367396	0.975679	0.632604	0.1236412	
365	29/06/2030		0.023324	0.360296	0.976676	0.639704	0.1190379	
366	30/06/2030		0.022046	0.353286	0.977954	0.646714	0.1147227	
367	01/07/2030		0.020643	0.346369	0.979357	0.653631	0.1106171	
368	02/07/2030		0.019516	0.339545	0.980484	0.660455	0.106536	
369	03/07/2030		0.018984	0.332814	0.981016	0.667186	0.1023381	
370	04/07/2030		0.018259	0.326179	0.981741	0.673821	0.0983744	
371	05/07/2030		0.017155	0.319639	0.982845	0.680361	0.0947188	
372	06/07/2030		0.01608	0.313196	0.98392	0.686804	0.0911865	
373	07/07/2030		0.015806	0.306849	0.984194	0.693151	0.0874483	
374	08/07/2030		0.014607	0.300599	0.985393	0.699401	0.0842341	
375	09/07/2030		0.013743	0.294447	0.986257	0.705553	0.0810055	
376	10/07/2030		0.01353	0.288392	0.98647	0.711608	0.0776355	
377	11/07/2030		0.012853	0.282435	0.987147	0.717565	0.074579	
378	12/07/2030		0.012342	0.276568	0.987658	0.723432	0.0715709	
379	13/07/2030		0.011558	0.270792	0.988442	0.729208	0.0687827	
380	14/07/2030		0.010743	0.264932	0.989257	0.735068	0.0660227	
381	15/07/2030		0.010264	0.25934	0.989736	0.74066	0.0633321	
382								
383								
384								
385								
386								
387								
388								
389								
390								
391								
392								
393								
394								
395								
396								

7.5 Eliminate the selected data (unnecessary data).

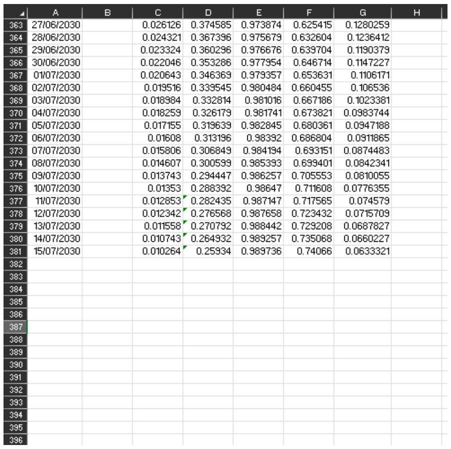

	A	B	C	D	E	F	G	H
363	27/06/2030		0.026126	0.374585	0.973874	0.625415	0.1280259	
364	28/06/2030		0.024321	0.367396	0.975679	0.632604	0.1236412	
365	29/06/2030		0.023324	0.360296	0.976676	0.639704	0.1190379	
366	30/06/2030		0.022046	0.353286	0.977954	0.646714	0.1147227	
367	01/07/2030		0.020643	0.346369	0.979357	0.653631	0.1106171	
368	02/07/2030		0.019516	0.339545	0.980484	0.660455	0.106536	
369	03/07/2030		0.018984	0.332814	0.981016	0.667186	0.1023381	
370	04/07/2030		0.018259	0.326179	0.981741	0.673821	0.0983744	
371	05/07/2030		0.017155	0.319639	0.982845	0.680361	0.0947188	
372	06/07/2030		0.01608	0.313196	0.98392	0.686804	0.0911865	
373	07/07/2030		0.015806	0.306849	0.984194	0.693151	0.0874483	
374	08/07/2030		0.014607	0.300599	0.985393	0.699401	0.0842341	
375	09/07/2030		0.013743	0.294447	0.986257	0.705553	0.0810055	
376	10/07/2030		0.01353	0.288392	0.98647	0.711608	0.0776355	
377	11/07/2030		0.012853	0.282435	0.987147	0.717565	0.074579	
378	12/07/2030		0.012342	0.276568	0.987658	0.723432	0.0715709	
379	13/07/2030		0.011558	0.270792	0.988442	0.729208	0.0687827	
380	14/07/2030		0.010743	0.264932	0.989257	0.735068	0.0660227	
381	15/07/2030		0.010264	0.25934	0.989736	0.74066	0.0633321	
382								
383								
384								
385								
386								
387								
388								
389								
390								
391								
392								
393								
394								
395								
396								

7.6 Final result.

Figure S15. Extension of the formula contained in cell $G192$ and elimination of unnecessary data

SECTION 5: MODIFYING THE FITTING HYPERPARAMETERS

STEP 1:

Modify the interval for calculating the mean square deviation as shown in cell V3 presented in Figure S16. For this example, square deviations were evaluated in column G between rows 192 and 381. Therefore, the formula in cell V3 must be: "=RAIZ(PROMEDIO(G192:G381))."

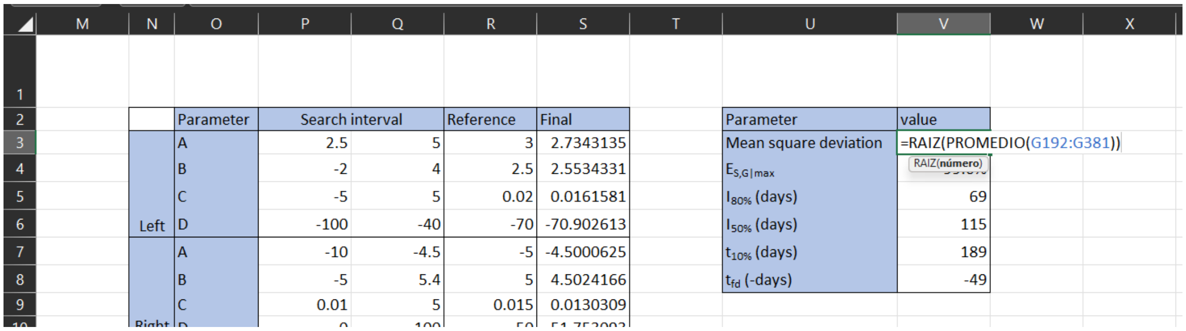

	N	O	P	Q	R	S
2		Parameter	Search interval		Reference	Final
3	Left	A	2.5	5	3	2.7343135
4		B	-2	4	2.5	2.5534331
5		C	-5	5	0.02	0.0161581
6		D	-100	-40	-70	-70.902613
7	Right	A	-10	-4.5	-5	-4.5000625
8		B	-5	5.4	5	4.5024166
9		C	0.01	5	0.015	0.0130309
10		D	[illegible]	[illegible]	[illegible]	[illegible]

	U	V
2	Parameter	value
3	Mean square deviation	=RAIZ(PROMEDIO(G192:G381))
4	$E_{S,G\|max}$	[illegible]
5	$I_{80\%}$ (days)	69
6	$I_{50\%}$ (days)	115
7	$t_{10\%}$ (days)	189
8	t_{fd} (-days)	-49

Figure S16. Area in the worksheet presenting the characteristic hyperparameters of the resulting curve for the vaccine effectiveness.

STEP 2:

Copy (**Ctrl + C**) the recommended values to start the search from the column named "Reference" (values from R3 to R10) and paste (**Ctrl + V**) them into the column named "Final" (S3 to S10), as shown in Figure S17.

BEFORE

	Parameter	Search interval		Reference	Final
	A	2.5	5	3	2.73431348
	B	-2	4	2.5	2.55343308
	C	-5	5	0.02	0.01615815
Left	D	-100	-40	-70	-70.9026133
	A	-10	-4.5	-5	-4.50006254
	B	-5	5.4	5	4.50241657
	C	0.01	5	0.015	0.01303086
Right	D	0	100	50	51.7530934

AFTER

	Parameter	Search interval		Reference	Final
	A	2.5	5	3	3
	B	-2	4	2.5	2.5
	C	-5	5	0.02	0.02
Left	D	-100	-40	-70	-70
	A	-10	-4.5	-5	-5
	B	-5	5.4	5	5
	C	0.01	5	0.015	0.015
Right	D	0	100	50	50

Figure S17. Insertion of the initial values

SECTION 6: SOLVER ADD-IN ENABLING

To enable the solver plugin, it is necessary to follow the next procedure (extracted from: https://support.microsoft.com/es-es/office/carga-del-complemento-solver-en-excel-2016-612926fc-d53b-46b4-872c-e24772f078ca).

STEP 1:

Click on "Archivo" (Figure S18)

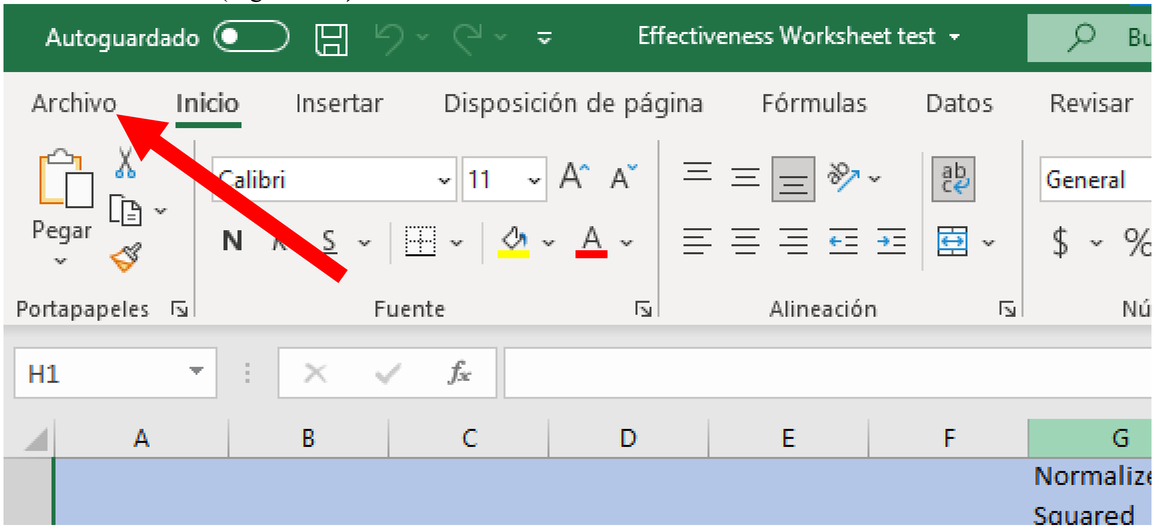

Figure S18. "Archivo" button localization (Click on "Archivo")

STEP 2:

Click on "Opciones" (Figure S19)

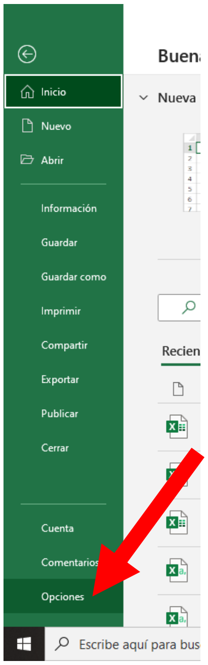

Figure S19. Options menu from the section named: "Archivo."

STEP 3:

After clicking on "Opciones," the window in Figure S20 will appear. Then, click "Complementos" (see the arrow in Figure S20).

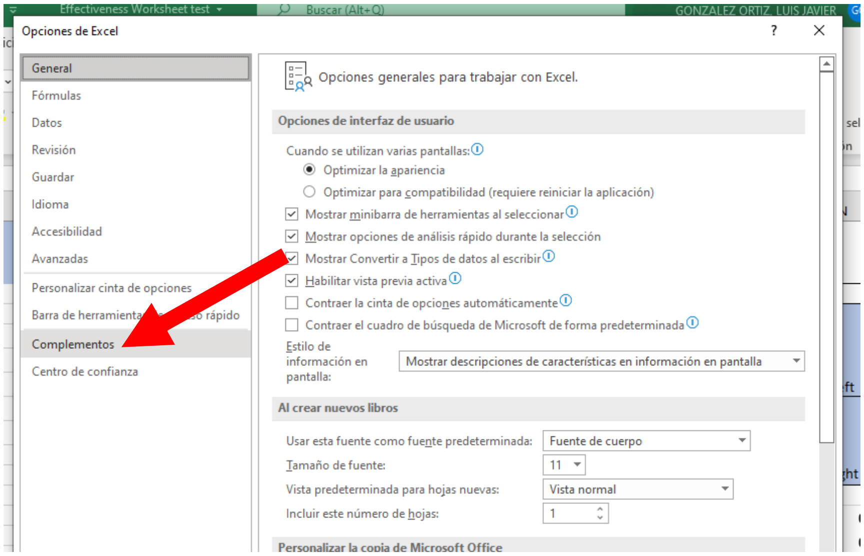

Figure S20. Options menu

STEP 4:

After clicking on "Complementos," it will appear the window presented in Figure S21. Then, click on "Solver" and later, click on "Ir," as shown in Figure S21.

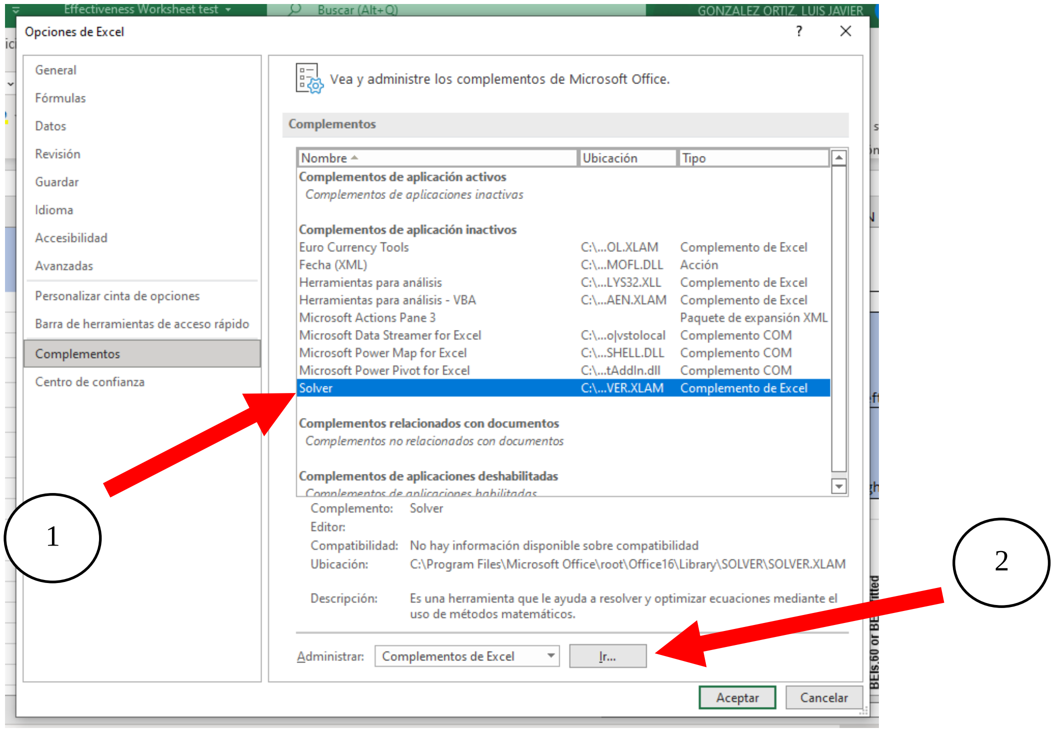

Figure S21. Solver component selection

STEP 5:

After clicking, it will appear a panel as shown in Figure S22

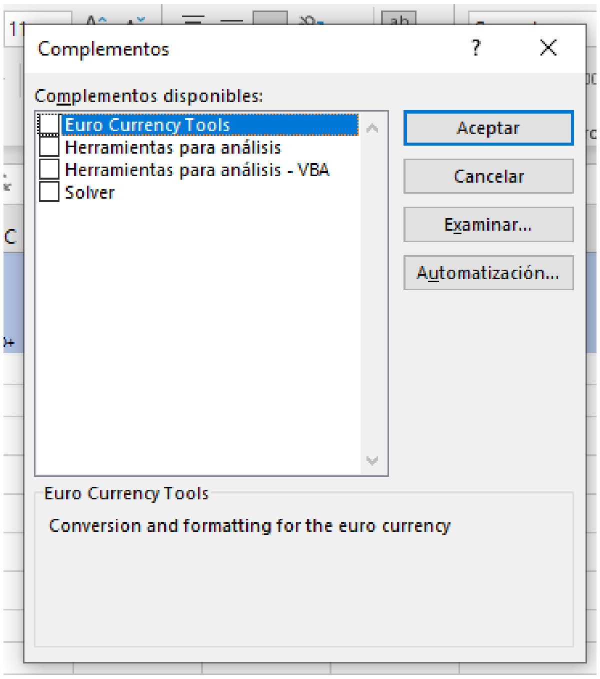

Figure S22. Component selection panel

STEP 6: Check "Solver" and later click on "Aceptar," as shown in Figure S23

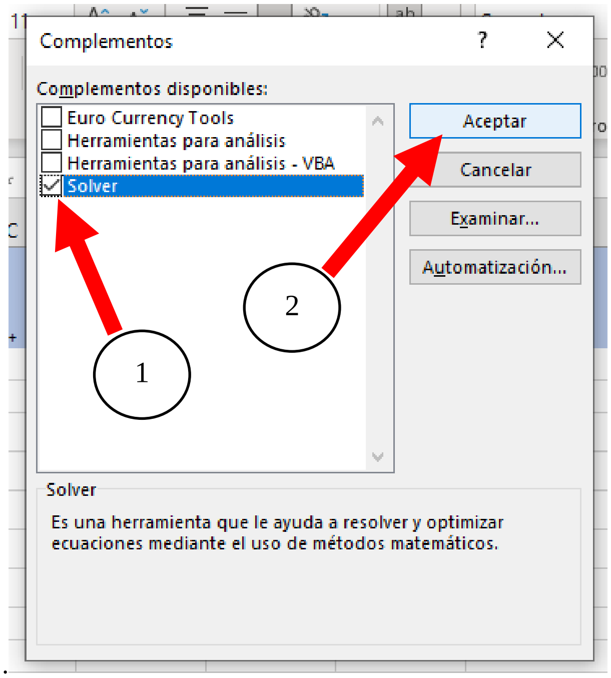

Figure S23. Solver complement enabling.

SECTION 7: USING "SOLVER" TO OBTAIN BY FITTING THE OBJECTIVE FUNCTION

STEP 1:

Click on "Datos" and later click on "Solver," as shown in Figure S24.

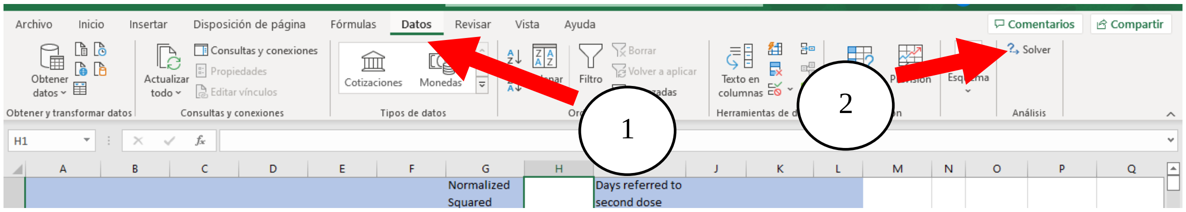

Figure S24. Solver button location. After clicking on "Solver," a window like in Figure S25 will appear.

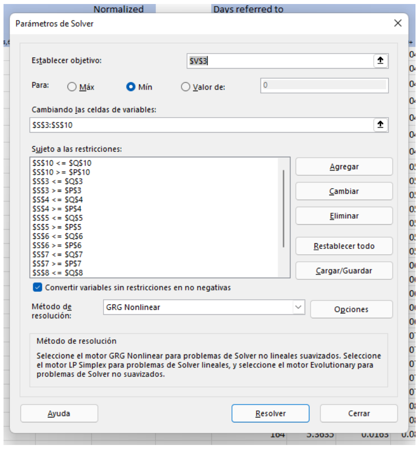

Figure S25. Solver configuration menu

Such a window is already preprogrammed with the search limits available in the cells from column P to Q, rows two to ten. If the **USERS** require other limits, they can be manually specified in such cells of the worksheet, and the system will automatically upload them. It is convenient to verify the preprogrammed limits to prevent eventual problems with the preprogrammed worksheet.

STEP 2:

Click on "Resolver," as shown in Figure S26.

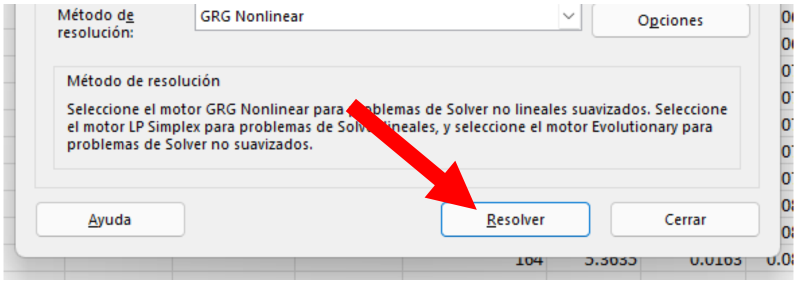

Figure S26. "Resolver" button location

After the click, the **USER's** device will start searching for the best-fitting profile (usually, this process will take less than one minute). Once it finishes the search, a prompt will appear asking if the **USER** wants to conserve the solver's found solution or want to keep the initial one (as shown in Figure S27); there, the **USER** must select "Conservar solución de Solver" and click on "Aceptar."

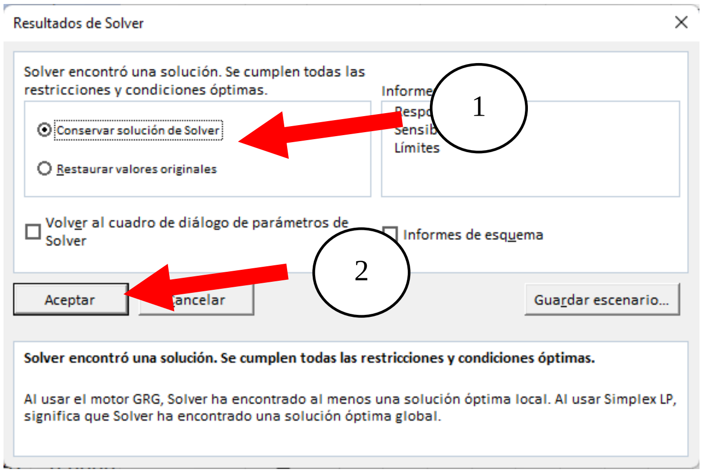

Figure S27. Prompt to accept solver solution

STEP 3:

Adjust the dates' interval of the $BE_{S,G}$ plot to visualize the **USER**'s experimental $BE_{S,G}$ profile and the $BE_{S,G,fitted}$ profile, as shown in Figure S28.

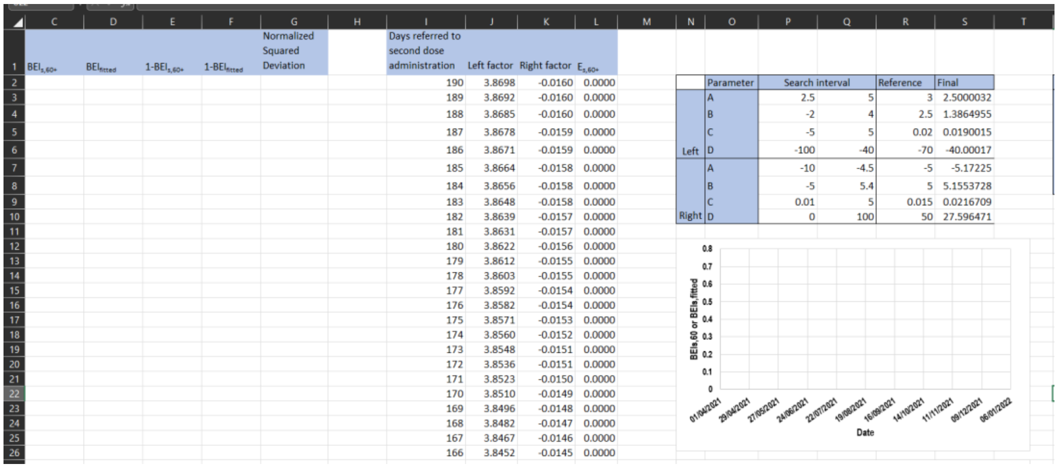

	C	D	E	F	G	H	I	J	K	L
1	$BEI_{s,60+}$	BEI_{fitted}	$1\text{-}BEI_{s,60+}$	$1\text{-}BEI_{fitted}$	Normalized Squared Deviation		Days referred to second dose administration	Left factor	Right factor	$E_{s,60+}$
2							190	3.8698	-0.0160	0.0000
3							189	3.8692	-0.0160	0.0000
4							188	3.8685	-0.0160	0.0000
5							187	3.8678	-0.0159	0.0000
6							186	3.8671	-0.0159	0.0000
7							185	3.8664	-0.0158	0.0000
8							184	3.8656	-0.0158	0.0000
9							183	3.8648	-0.0158	0.0000
10							182	3.8639	-0.0157	0.0000
11							181	3.8631	-0.0157	0.0000
12							180	3.8622	-0.0156	0.0000
13							179	3.8612	-0.0155	0.0000
14							178	3.8603	-0.0155	0.0000
15							177	3.8592	-0.0154	0.0000
16							176	3.8582	-0.0154	0.0000
17							175	3.8571	-0.0153	0.0000
18							174	3.8560	-0.0152	0.0000
19							173	3.8548	-0.0151	0.0000
20							172	3.8536	-0.0151	0.0000
21							171	3.8523	-0.0150	0.0000
22							170	3.8510	-0.0149	0.0000
23							169	3.8496	-0.0148	0.0000
24							168	3.8482	-0.0147	0.0000
25							167	3.8467	-0.0146	0.0000
26							166	3.8452	-0.0145	0.0000

	Parameter	Search interval		Reference	Final
Left	A	2.5	5	3	2.5000032
	B	-2	4	2.5	1.3864955
	C	-5	5	0.02	0.0190015
	D	-100	-40	-70	-40.00017
Right	A	-10	-4.5	-5	-5.17225
	B	-5	5.4	5	5.1553728
	C	0.01	5	0.015	0.0216709
	D	0	100	50	27.596471

BEFORE

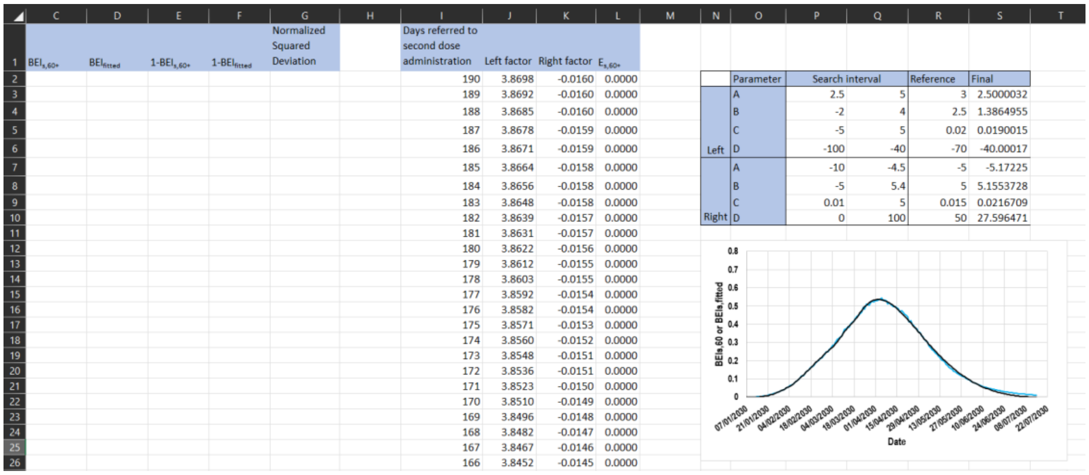

	C	D	E	F	G	H	I	J	K	L
1	$BEI_{s,60+}$	BEI_{fitted}	$1\text{-}BEI_{s,60+}$	$1\text{-}BEI_{fitted}$	Normalized Squared Deviation		Days referred to second dose administration	Left factor	Right factor	$E_{s,60+}$
2							190	3.8698	-0.0160	0.0000
3							189	3.8692	-0.0160	0.0000
4							188	3.8685	-0.0160	0.0000
5							187	3.8678	-0.0159	0.0000
6							186	3.8671	-0.0159	0.0000
7							185	3.8664	-0.0158	0.0000
8							184	3.8656	-0.0158	0.0000
9							183	3.8648	-0.0158	0.0000
10							182	3.8639	-0.0157	0.0000
11							181	3.8631	-0.0157	0.0000
12							180	3.8622	-0.0156	0.0000
13							179	3.8612	-0.0155	0.0000
14							178	3.8603	-0.0155	0.0000
15							177	3.8592	-0.0154	0.0000
16							176	3.8582	-0.0154	0.0000
17							175	3.8571	-0.0153	0.0000
18							174	3.8560	-0.0152	0.0000
19							173	3.8548	-0.0151	0.0000
20							172	3.8536	-0.0151	0.0000
21							171	3.8523	-0.0150	0.0000
22							170	3.8510	-0.0149	0.0000
23							169	3.8496	-0.0148	0.0000
24							168	3.8482	-0.0147	0.0000
25							167	3.8467	-0.0146	0.0000
26							166	3.8452	-0.0145	0.0000

	Parameter	Search interval		Reference	Final
Left	A	2.5	5	3	2.5000032
	B	-2	4	2.5	1.3864955
	C	-5	5	0.02	0.0190015
	D	-100	-40	-70	-40.00017
Right	A	-10	-4.5	-5	-5.17225
	B	-5	5.4	5	5.1553728
	C	0.01	5	0.015	0.0216709
	D	0	100	50	27.596471

AFTER

Figure S28. Adjustment of the dates' interval of the $BE_{S,G}$ plot

SECTION 8: RESULTS

After this procedure, the **USER**'s worksheet must look like the one shown in Figure S29, where the following information can be appreciated.

A: Effectiveness profile tabulated for every day in the defined effectivity interval (column L).
B: $BE_{S,G}$ and $BE_{S,G,fitted}$ profiles (superior plot).
C: Effectiveness profile estimated for the interest group (inferior plot).
D: Eight mathematical parameters, which are required to plot the estimated effectiveness profile (column S).
E: Characteristic parameters of the effectiveness profile (column V).
F: Root mean square deviation for the considered case (expressed in percentage; cell $V3$); for this case: 1.0%

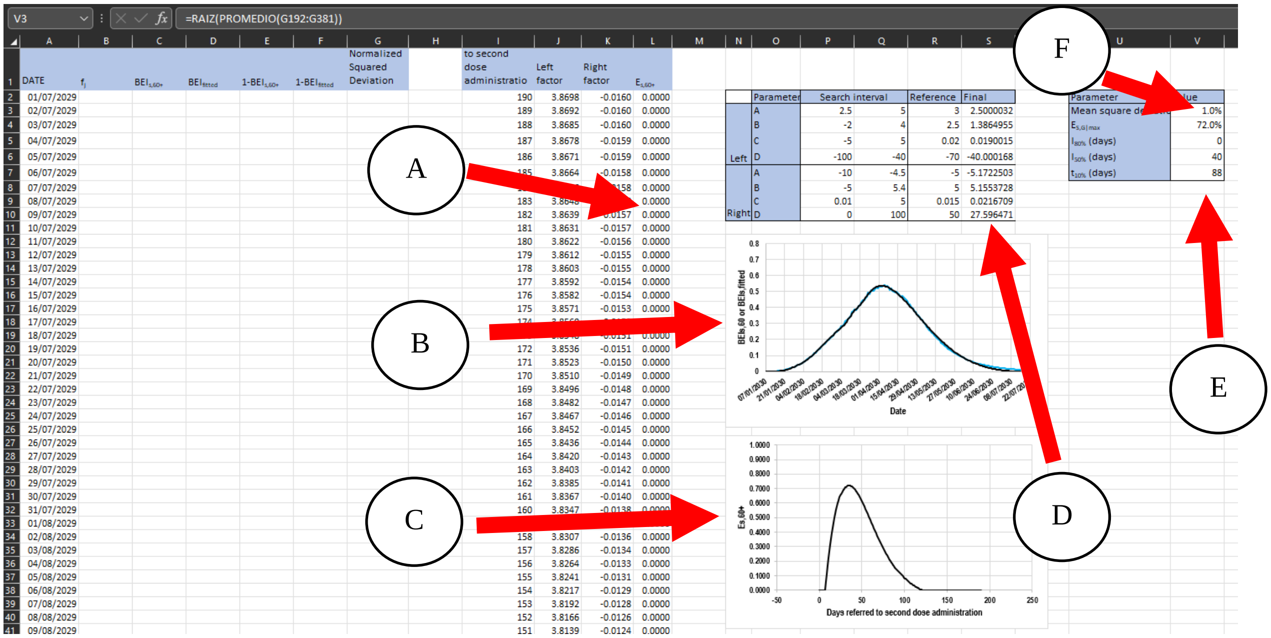

Figure S29. Obtained results

Note: Occasionally, refining the obtained result (shown in cells S2 to S10) could be convenient by repeating the fitting process but using the previously obtained result (close to the correct result) as the new starting point.

After the whole procedure, the worksheet will appear as shown in file Supplementary Material # 7, specifically into the worksheet named “Final fitting.”
